# Effect of cardioplegic arrest and reperfusion on left and right ventricular proteome/phosphoproteome in patients undergoing surgery for coronary or aortic valve disease

**DOI:** 10.3892/ijmm.2022.5133

**Published:** 2022-04-14

**Authors:** Safa Abdul-Ghani, Katie L. Skeffington, Minjoo Kim, Marco Moscarelli, Philip A. Lewis, Kate Heesom, Francesca Fiorentino, Costanza Emanueli, Barnaby C. Reeves, Prakash P. Punjabi, Gianni D. Angelini, M-Saadeh Suleiman

**Affiliations:** 1 Bristol Heart Institute and Bristol Medical School, University of Bristol, Bristol BS2 8HW, UK; 2 Department of Physiology, Faculty of Medicine, Al-Quds University, Abu-Dis, Palestine; 3 National Heart and Lung Institute, Imperial College, London SW3 6LY, UK; 4 GVM Care and Research, Anthea Hospital, I-70124 Bari, Italy; 5 University of Bristol Proteomics/Bioinformatics Facility, University of Bristol, Bristol BS8 1TD, UK

**Keywords:** proteomics, human, cardiac, coronary artery disease, aortic valve stenosis, cardioplegic arrest, ischemia, ventricular biopsies, TMT tag, mass spectrometry

## Abstract

Our earlier work has shown inter-disease and intra-disease differences in the cardiac proteome between right (RV) and left (LV) ventricles of patients with aortic valve stenosis (AVS) or coronary artery disease (CAD). Whether disease remodeling also affects acute changes occuring in the proteome during surgical intervention is unknown. This study investigated the effects of cardioplegic arrest on cardiac proteins/phosphoproteins in LV and RV of CAD (n=6) and AVS (n=6) patients undergoing cardiac surgery. LV and RV biopsies were collected during surgery before ischemic cold blood cardioplegic arrest (pre) and 20 min after reperfusion (post). Tissues were snap frozen, proteins extracted, and the extracts were used for proteomic and phosphoproteomic analysis using Tandem Mass Tag (TMT) analysis. The results were analysed using QuickGO and Ingenuity Pathway Analysis softwares. For each comparision, our proteomic analysis identified more than 3,000 proteins which could be detected in both the pre and Post samples. Cardioplegic arrest and reperfusion were associated with significant differential expression of 24 (LV) and 120 (RV) proteins in the CAD patients, which were linked to mitochondrial function, inflammation and cardiac contraction. By contrast, AVS patients showed differential expression of only 3 LV proteins and 2 RV proteins, despite a significantly longer duration of ischaemic cardioplegic arrest. The relative expression of 41 phosphoproteins was significantly altered in CAD patients, with 18 phosphoproteins showing altered expression in AVS patients. Inflammatory pathways were implicated in the changes in phosphoprotein expression in both groups. Inter-disease comparison for the same ventricular chamber at both timepoints revealed differences relating to inflammation and adrenergic and calcium signalling. In conclusion, the present study found that ischemic arrest and reperfusion trigger different changes in the proteomes and phosphoproteomes of LV and RV of CAD and AVS patients undergoing surgery, with markedly more changes in CAD patients despite a significantly shorter ischaemic period.

## Introduction

Experimental models have been used to study the molecular changes associated with cardiac diseases. However, these models have significant drawbacks and do not always reflect the clinical setting. For example, the commonly used nontransgenic models of coronary artery disease involve acute ischemia rather than progressive atherosclerotic coronary disease which triggers slow progressive cardiac remodeling (1). It is highly informative to study human hearts directly; however, the collection of samples is challenging. Studies using human cardiac ventricular tissues for molecular investigations are few and have largely focused on global gene expression in failing and non-failing donor hearts and comparing atrial with ventricular tissue (2–5). For example, a study comparing gene expression in right atrial appendage and left ventricle of patients undergoing two types of cardiac surgery (coronary artery bypass graft and aortic valve replacement) identified ~2% of the detected genes as differentially expressed (6). Although gene expression profiling is informative, it is not conclusive as it may not always reflect changes in protein levels associated with disease progression (7). For this reason, we have carried out comparative studies investigating disease-induced changes in protein expression in congenital hearts (8–10) and in adult patients with either aortic valve stenosis (AVS) or coronary artery disease (CAD) (11). In the latter, we have shown that different cardiac diseases trigger different proteomic remodeling in the diseased left (LV) and relatively less diseased right ventricles (RV) and that the extent of remodeling varies with disease type.

Investigating the effect of disease on cardiac molecular changes provides critical information about signaling pathways to improve our understanding of disease-induced cardiac remodeling (11). However, information is also required concerning the acute molecular changes associated with cardiopulmonary bypass and cardioplegic ischemic arrest during open-heart surgery which are likely to have implications for postoperative morbidity. Distinguishing such changes from molecular changes due to chronic disease requires information gathered from pre- and post-operative levels. In the present study, we collected pre- and post-cardioplegic arrest biopsies from diseased LVs and relatively normal RVs of patients undergoing surgery for CAD or AVS in order to investigate the acute changes in protein expression during open heart surgery. We also compared differences in protein expression between CAD and AVS patients at each timepoint. This part of the study provides an update to our previous work (11); the data are improved due to the use of the latest proteomic technology which allows detection of a greater number of proteins as well as the analysis of relative phosphoprotein expression, a larger n number of available samples and the inclusion of a comparison of post-reperfusion samples. The information collected will help in the design of cardioprotective interventions taking into consideration the involvement of pathology and ventricular chambers.

## Materials and methods

### Patients and tissue collection

The data presented in this work are a sub-study of a two-centre randomized controlled trial investigating the effect of the upper limb remote ischemic preconditioning (RIPC) in patients undergoing isolated coronary artery bypass grafting and/or aortic valve replacement on cardiac injury, metabolic stress and inflammatory response (12,13). The trial was conducted in accordance with the Declaration of Helsinki at the Hammersmith Hospital and the Bristol Royal Infirmary. It was sponsored by the Imperial College of London, approved by the London-Harrow Research Ethics Committee (REC number 12/LO/1361) and registered to the International Standard Randomized Controlled Trial Number (ISRCTN) registry (ID 33084113).

Inclusion, exclusion criteria and trial conduct were previously published (12,13). A group of patients who had either CAD (n=6) or AVS (n=6) were randomly selected from the trial and used for this sub-study. All 6 CAD patients had 3 grafts for diseases in the following vessels: left anterior descending, obtuse marginal branch and the right coronary artery. One patient had an additional left main disease. Both CAD and AVS patients were control patients; no patients who received RIPC intervention were included. Frozen biopsies were obtained using a Trucut needle from the left and the right ventricles pre and Post cardioplegic arrest during coronary artery bypass graft or aortic valve surgery. However, a few biopsies did not yield enough protein for the proteomic analysis and were therefore not included. In total 40 biopsies were processed (n=20 from CAD and n=20 from AVS patients).

### Anesthesia, surgery and cardioplegia management

Anesthetic management, cardiopulmonary bypass (CPB), cardioplegia, surgical techniques and any other aspect of pre- and post-operative management were in accordance with existing protocols at both centers. Surgery proceeded as per routine practice in each center. Cardioplegic ischemic arrest was induced using cold cardioplegia (4 parts blood:1 part cardioplegia ratio) given at a temperature of approximately 4°C as described in detail elsewhere (12,13). In agreement with reported studies and clinical practice, the cross-clamp time was significantly longer in AVS patients (Table I).

### Sample preparation

Proteins were extracted in radioimmunoprecipitation assay (RIPA) buffer (1% NP-40, 0.5% sodium deoxycholate, 0.1% SDS, in PBS) containing phosphatase and protease inhibitors, and quantified using the Bradford assay. Aliquots of 100 *µ*g of 10 samples (including a reference sample prepared as a mixture from all samples) per experiment were digested with trypsin (2.5 *µ*g trypsin per 100 *µ*g protein; 37°C, overnight), labelled with Tandem Mass Tag (TMT) 10Plex reagents according to the manufacturer's protocol (Thermo Fisher Scientific, Inc.), and all the labelled samples were pooled. For the total proteome analysis, aliquots of 50 *µ*g of the pooled sample were evaporated to dryness and re-suspended in buffer A (20 mM ammonium hydroxide, pH 10.0) prior to fractionation by high pH reversed-phase chromatography using an Ultimate 3000 liquid chromatography system (Thermo Fisher Scientific, Inc.). The samples were processed in randomly selected groups of 9, with the same pooled sample included in each run to allow comparison between runs. The samples were loaded onto an XBridge BEH C18 Column (130 Å, 3.5 *µ*m, 2.1×150 mm, Waters) in buffer A and peptides eluted with an increasing gradient of buffer B (20 mM ammonium hydroxide in acetonitrile, pH 10.0) from 0–95% over 60 min. The resulting fractions were evaporated to dryness and re-suspended in 1% formic acid prior to analysis by nano-LC MSMS using an Orbitrap Fusion Tribrid mass spectrometer (Thermo Fisher Scientific, Inc.).

For the phosphoproteome analysis, the remainder of the TMT-labelled pooled sample was evaporated to dryness and subjected to TiO_2_-based phosphopeptide enrichment according to the manufacturer's instructions (Pierce/Thermo Fisher Scientific, Inc.). The phospho-enriched sample was evaporated to dryness and then re-suspended in 1% formic acid prior to analysis by nano-LC MSMS using an Orbitrap Fusion Tribrid mass spectrometer (Thermo Fisher Scientific, Inc.).

### Nano-LC mass spectrometry

High pH RP fractions (total proteome analysis) or the phospho-enriched fraction (phospho-proteome analysis) were further fractionated using an Ultimate 3000 nano-HPLC system in line with an Orbitrap Fusion Tribrid mass spectrometer (Thermo Fisher Scientific, Inc.). In brief, peptides in 1% (vol/vol) formic acid were injected onto an Acclaim PepMap C18 nano-trap column (Thermo Fisher Scientific, Inc.). After washing with 0.5% (vol/vol) acetonitrile 0.1% (vol/vol) formic acid, peptides were resolved on a 250 mm × 75 *µ*m Acclaim PepMap C18 reverse phase analytical column (Thermo Fisher Scientific, Inc.) over a 150 min organic gradient, using seven gradient segments (1–6% solvent B over 1 min, 6–15% B over 58 min, 15–32% B over 58 min, 32–40% B over 5 min, 40–90% B over 1 min, held at 90% B for 6 min and then reduced to 1% B over 1 min) with a flow rate of 300 nl·min^−1^. Solvent A was 0.1% formic acid and Solvent B was aqueous 80% acetonitrile in 0.1% formic acid. Peptides were ionized by nano-electrospray ionization at 2.0 kV using a stainless-steel emitter with an internal diameter of 30 *µ*m (Thermo Fisher Scientific, Inc.) and a capillary temperature of 275°C.

All spectra were acquired using an Orbitrap Fusion Tribrid mass spectrometer controlled by Xcalibur 3.0 software (Thermo Fisher Scientific, Inc.) and operated in data-dependent acquisition mode using an SPS-MS3 workflow. FTMS1 spectra were collected at a resolution of 120,000, with an automatic gain control (AGC) target of 200,000 and a maximum injection time of 50 msec. The Top N most intense ions were selected for MS/MS. Precursors were filtered according to charge state (to include charge states 2–7) and with mono-isotopic precursor selection. Previously interrogated precursors were excluded using a dynamic window (40 sec +/-10 ppm). The MS2 precursors were isolated with a quadrupole mass filter set to a width of 1.2 m/z. ITMS2 spectra were collected with an AGC target of 5,000, max injection time of 120 msec and CID collision energy of 35%.

For FTMS3 analysis, the Orbitrap was operated at 60,000 resolution with an AGC target of 50,000 and a max injection time of 120 msec. Precursors were fragmented by high-energy collision dissociation (HCD) at normalized collision energy of 55% to ensure maximal TMT reporter ion yield. Synchronous Precursor Selection (SPS) was enabled to include up to five MS2 fragment ions in the FTMS3 scan.

### Data processing and analysis

The raw data files were processed and quantified using Proteome Discoverer software version 1.4 (Thermo Fisher Scientific, Inc.) and peptide sequences searched against the Uniprot Human database (134,169 sequences) using the SEQUEST algorithm (https://www.uniprot.org/)Peptide precursor mass tolerance was set at 10 ppm, and MS/MS tolerance was set at 0.6 Da. Search criteria included oxidation of methionine (+15.9949) as a variable modification and carbamido-methylation of cysteine (+57.0214) and the addition of the TMT mass tag (+229.163) to peptide N-termini and lysine as fixed modifications. For the phosphoproteome analysis, phosphorylation of serine, threonine and tyrosine (+79.966) were also included as variable modifications. Searches were performed with full tryptic digestion and a maximum of one missed cleavage was allowed. The reverse database search option was enabled and all peptide data were filtered to satisfy a false discovery rate (FDR) of 5%.

Only proteins or phosphoproteins that were detected in all biopsies for each comparison were included in the analysis (14). Values are presented as a ratio to the internal standard (a pool of all samples) and represent the median of the measured peptide(s) for each protein. Values for phosphoproteins are expressed relative to the total protein expression-this allows determination of genuine changes in levels of phosphorylation rather than changes in the overall abundance of a protein whose phosphorylation level remains unchanged. Fold changes (Post reperfusion/pre ischemic cardioplegic arrest and AVS/CAD) in biopsies were calculated, and log_2_(fold change) was plotted against-log_10_(P-value) on a volcano plot. A change in protein expression greater or less than 1.3× and with P<0.05 (Student's unpaired, two-tailed, t-test) was considered significant. These cut-offs were chosen in accordance with previous studies (9,11,15). No further correction for multiple testing was made; several features of proteomics studies such as this one (e.g. small n numbers and ratio compression caused by TMT tagging) are known to make it difficult to apply multiple correction techniques effectively, often leading to a significant number of false-positive results (16). Proteins or phosphoproteins with a significant difference in expression between pre and post samples or between AVS and CAD samples (P<0.05) were loaded into Ingenuity Pathway Analysis (IPA) software (QIAGEN Inc., https://www.qiagenbioinformatics.com/products/ingenuitypathway-analysis) to determine the significantly (P<0.05) enriched canonical pathways (P-value of overlap calculated by Fisher's exact test right tailed). For the comparison between pathologies, all proteins were included in the IPA analysis, not only those that were present in every biopsy. The IPA analysis is routinely used, recommended, and was purchased by our Proteomics Centre and our bioinformatics specialist. IPA is a well-known and extensively used software package for proteomics data analysis as confirmed by the large number of peer-reviewed publications reporting its use. Quick Go software (https://www.ebi.ac.uk/QuickGO/) was used to analyze gene ontology (GO) enrichment and heatmaps were plotted using R software (https://www.r-project.org/; version 3.5.1). In an earlier study, we carried out western blotting analysis on selected proteins extracted from human cardiac biopsies and showed strong correlation with our proteomic analysis thus providing some support for the validity of our analytical methods (11).

## Results

### Effect of ischemic cardioplegic arrest on cardiac proteome/phosphoproteome in LV and RV of patients with coronary artery disease (CAD)

Approximately 7,000 proteins were detected over all the samples in this study. However, only 3,371 proteins were detected in all pre and post ischemic biopsies collected from the LV of CAD patients (n=6 and n=5 for pre and post samples respectively) and 3,120 proteins were detected in all pre and post biopsies collected from the RV of CAD patients (n=6 and n=3). Only these proteins were used in our analysis (see exclusions in Materials and methods).

**Figure 1:**
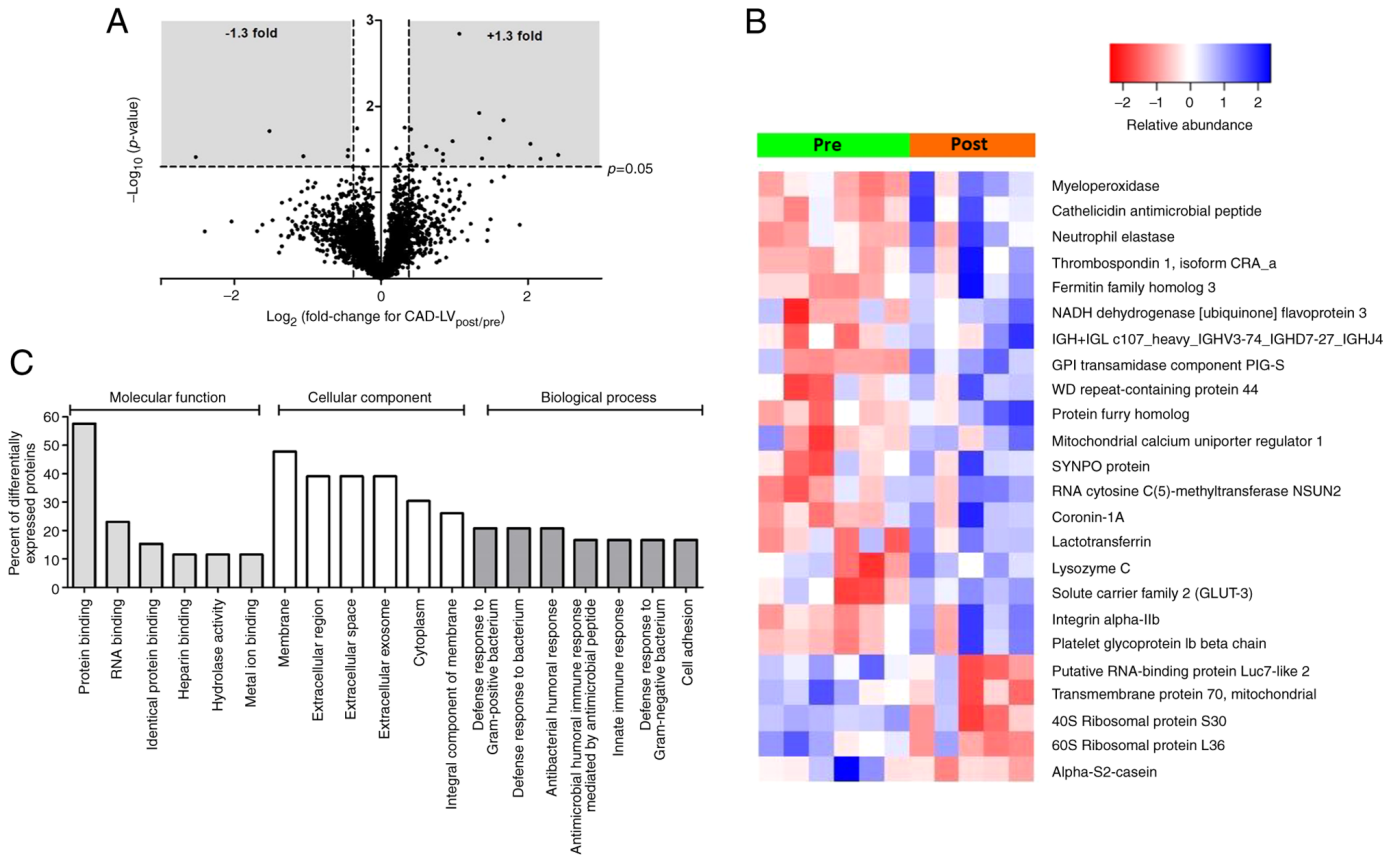
Proteomic changes in LV of CAD patients. (A) Volcano plot of the entire set of proteins quantified before ischemic cardioplegic arrest (pre) and after reperfusion (post) in LV of CAD patients. Each point represents the log2(fold change) between pre and post protein levels, plotted against the associated level of statistical significance for the fold change. Proteins in the shaded area (>1.3 or <0.769-fold change, P<0.05) are considered to be differentially expressed. (B) Heat map showing individual protein levels in each biopsy for the differentially expressed proteins from the LV of CAD patients. (C) The summary of the functional categorization by Gene Ontology (GO) analysis of the proteins differentially expressed between pre and post sample in the LV of CAD patients. The top 6 or 7 GO terms in each of the three main categories of GO classification (molecular function, cellular component and biological process) are displayed. The y-axis represents the percentage of a specific category of proteins within the main category. LV, left ventricle; CAD, coronary artery disease.

Fig. 1A shows a volcano plot for the changes (post/pre ischemia) in protein expression found in the LV of CAD patients as a result of ischemic cardioplegic arrest and reperfusion. The expression of 24 proteins were significantly altered, of which 19 demonstrated increased expression and 5 decreased expression (Table SI). A heat map (Fig. 1B) shows the changes in individual biopsies. Several of the proteins with the greatest increase in expression are likely to have emanated from components of the systemic circulation (e.g. platelet glycoprotein Iβ, integrin α-IIb, neutrophil elastase and myeloperoxidase). Other proteins which were differentially expressed are of cardiac origin, such as the mitochondrial calcium uniporter regulator 1, NADH dehydrogenase flavoprotein 3 and GLUT3. Gene Ontology (GO) analysis of these proteins indicates involvement of biological processes that are associated with changes in immune responses (Fig. 1C) while IPA canonical pathway analysis highlighted significant enrichment of pathways involved in protein translation (eukaryotic initiation factors 2 and 4, Table II).

Phosphoprotein analysis for the LV of CAD patients revealed 28 phosphoproteins differentially expressed in pre vs. post samples when adjusted for total protein content (Table SII). A total of 23 phosphoproteins showed increased expression while 5 showed decreased expression. A volcano plot can be found in Fig. S1A. IPA canonical pathway analysis (Table III) highlighted enrichment of signalling pathways relating to several proinflammatory cytokines [interleukin (IL)-6 and IL-22].

**Figure 2:**
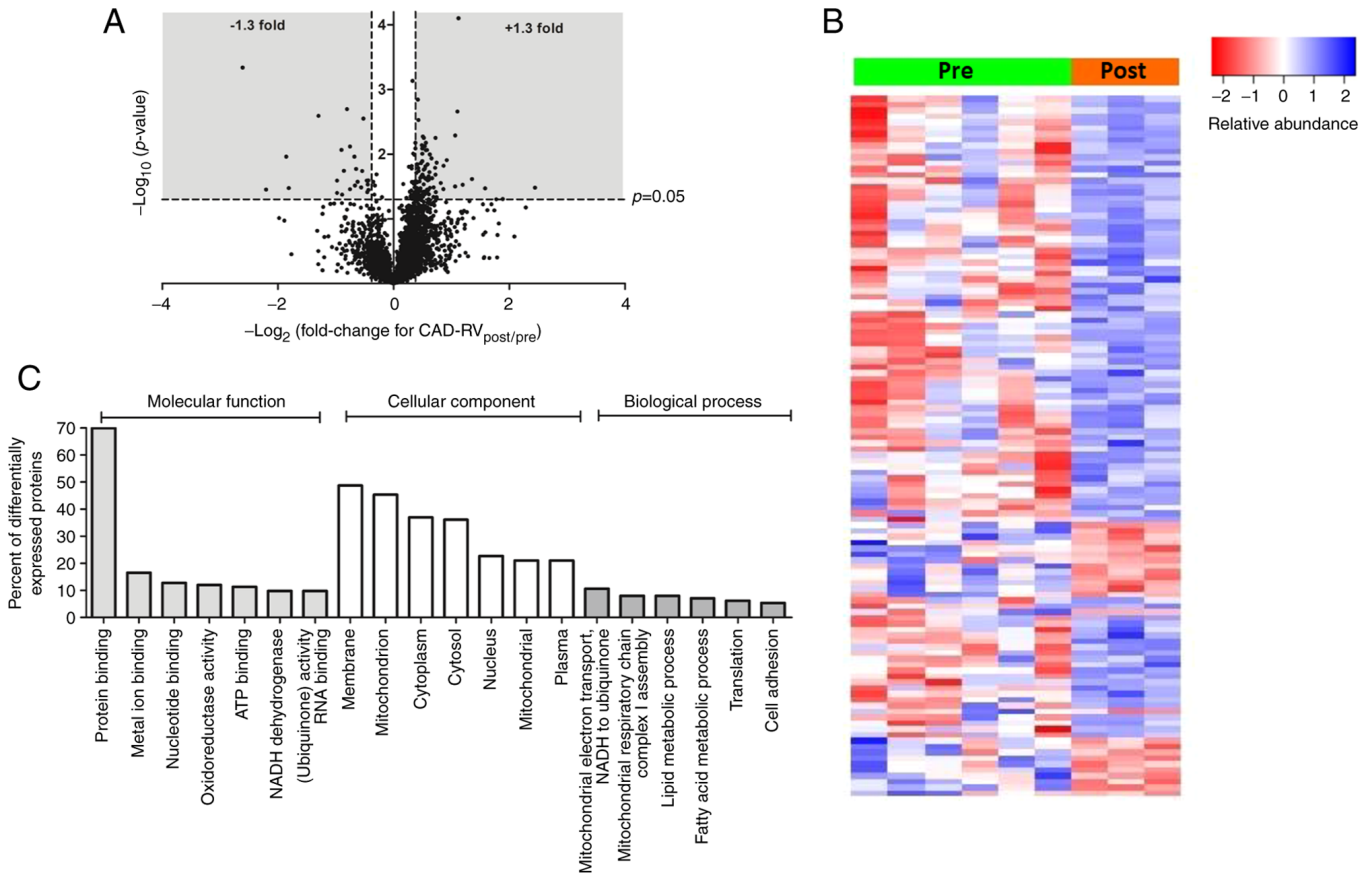
Proteomic changes in RV of CAD patients. (A) Volcano plot of the entire set of proteins quantified before ischemic cardioplegic arrest (pre) and after reperfusion (post) in RV of CAD patients. Each point represents the log2(fold change) between pre and post protein levels, plotted against the associated level of statistical significance for the fold change. Proteins in the shaded area (>1.3 or <0.769-fold change, P<0.05) are considered to be differentially expressed. (B) Heat map showing individual protein levels in each biopsy for the differentially expressed proteins from the RV of CAD patients. (C) The summary of the functional categorization by Gene Ontology (GO) analysis of the proteins differentially expressed between pre and post sample in the LV of CAD patients. The top 6 or 7 GO terms in each of the three main categories of GO classification (molecular function, cellular component and biological process) are displayed. The y axis represents the percentage of a specific category of proteins within the main category. RV, right ventricle; CAD, coronary artery disease.

In the RV of CAD patients, 120 proteins showed a significant change in expression between pre and post samples, with the majority (95 proteins) showing an increase in expression (Fig. 2A and Table SIII). Many of these are mitochondrial proteins, including several proteins associated with NADH dehydrogenase or the TCA cycle. As with the LV, there was also elevation of several proteins known to originate in the circulation (e.g. blood components). Several of the proteins that decreased in expression as a result of surgery were related to the contractile myofilaments. A heat map analysis shows the fold change (FC) levels for individual biopsies and clearly indicate that despite the smaller number of post biopsies, the effects were marked (Fig. 2B). Further ontology analysis of these proteins indicated involvement of biological processes that are associated with changes in metabolic/energetic processes, several of which relate to mitochondria (Fig. 2C). Similarly, IPA canonical pathway analysis (Table II) revealed mitochondrial dysfunction to be a highly enriched pathway (P-value of overlap 1.0×10^−11^).

Phosphoprotein analysis for the RV of CAD patients revealed 13 phosphoproteins differentially expressed in pre vs. post samples when adjusted for total protein content (9 with increased expression and 4 with decreased expression; Table SIV and Fig. S1B). Similarily for the LV, phosphoprotein analysis for this group of patients, IPA canonical pathway analysis of the RV phosphoproteome highlighted several inflammatory pathways as being significantly enriched, including IL-6 signalling, IL-22 signalling and acute phase response signalling (Table III).

### Effect of ischemic cardioplegic arrest on cardiac proteome/phosphoproteome in LV and RV of patients with aortic valve stenosis (AVS)

For AVS patients, 3,220 proteins were detected in all pre and post ischemic biopsies collected from the LV (n=6 and n=5 for pre and post samples respectively) and 3,208proteins were detected in all pre and post biopsies collected from the RV (n=5 and n=4). Only these proteins were used in our analysis (see exclusions in Materials and methods).

**Figure 3:**
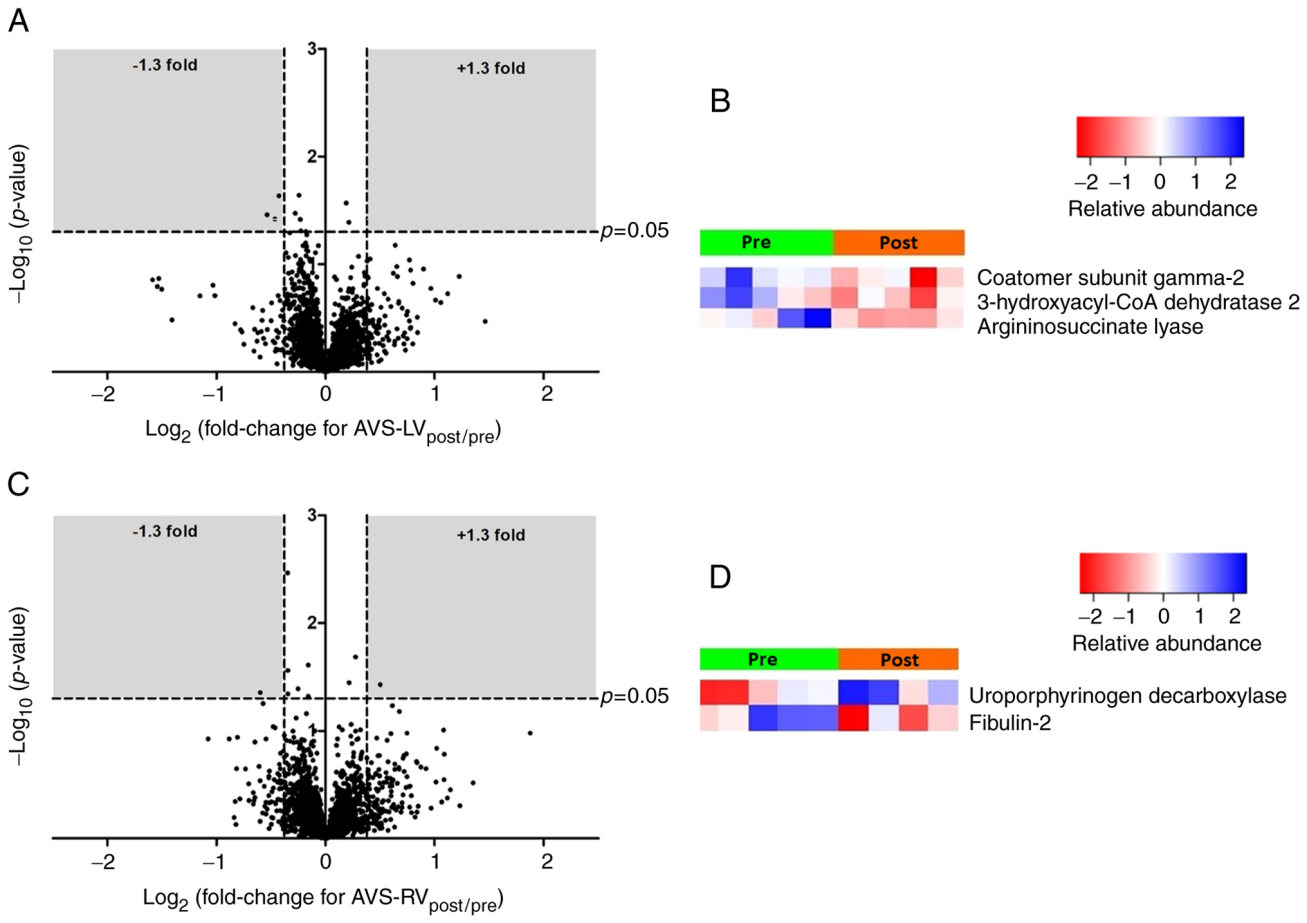
Proteomic changes in AVS patients. Volcano plots of the entire set of proteins quantified before ischemic cardioplegic arrest (pre) and after reperfusion (post) in LV (A) or RV (C) of AVS patients. Each point represents the log2(fold change) between pre and post protein levels, plotted against the associated level of statistical significance for the fold change. Proteins in the shaded area (>1.3 or <0.769-fold change, P<0.05) are considered to be differentially expressed. Also shown are heat maps showing individual protein levels in each biopsy for the differentially expressed proteins from the LV (B) and RV (D) of the AVS patients. LV, left ventricle; RV, right ventricle; AVS, aortic valve stenosis.

Fig. 3 shows volcano plots and heatmaps for the LV (Fig. 3A and B) and RV (Fig. 3C and D) of AVS patients. The differentially expressed proteins for both ventricles are listed in Table SV. In the LV, the expression of three proteins [coatomer subunit gamma-2, very-long-chain (3R)-3-hydroxyacyl-CoA dehydratase 2 and argininosuccinate lyase] was significantly decreased. In the RV, one protein showed increased expression (uroporphyrin decarboxylase) and one protein showed decreased expression (fibulin-2). IPA canonical pathway analysis (Table SVI) was uninformative given the small number of proteins involved.

Analysis of phosphoprotein expression (relative to total protein expression) in AVS patients (Table SVII) found 14 phosphoproteins with altered expression in the LV (11 increased and 3 decreased, Fig. S1C) and 4 proteins with altered expression in the RV (all with increased expression, Fig. S1D). Canonical pathway analysis (Table IV) found enrichment of several pro-inflammatory pathways, including IL-6 in both ventricles and IL-17 in the LV.

### Comparision of protein/phosphoprotein expression in AVS patients compared to CAD patients pre-ischaemic cardioplegic arrest

**Figure 4:**
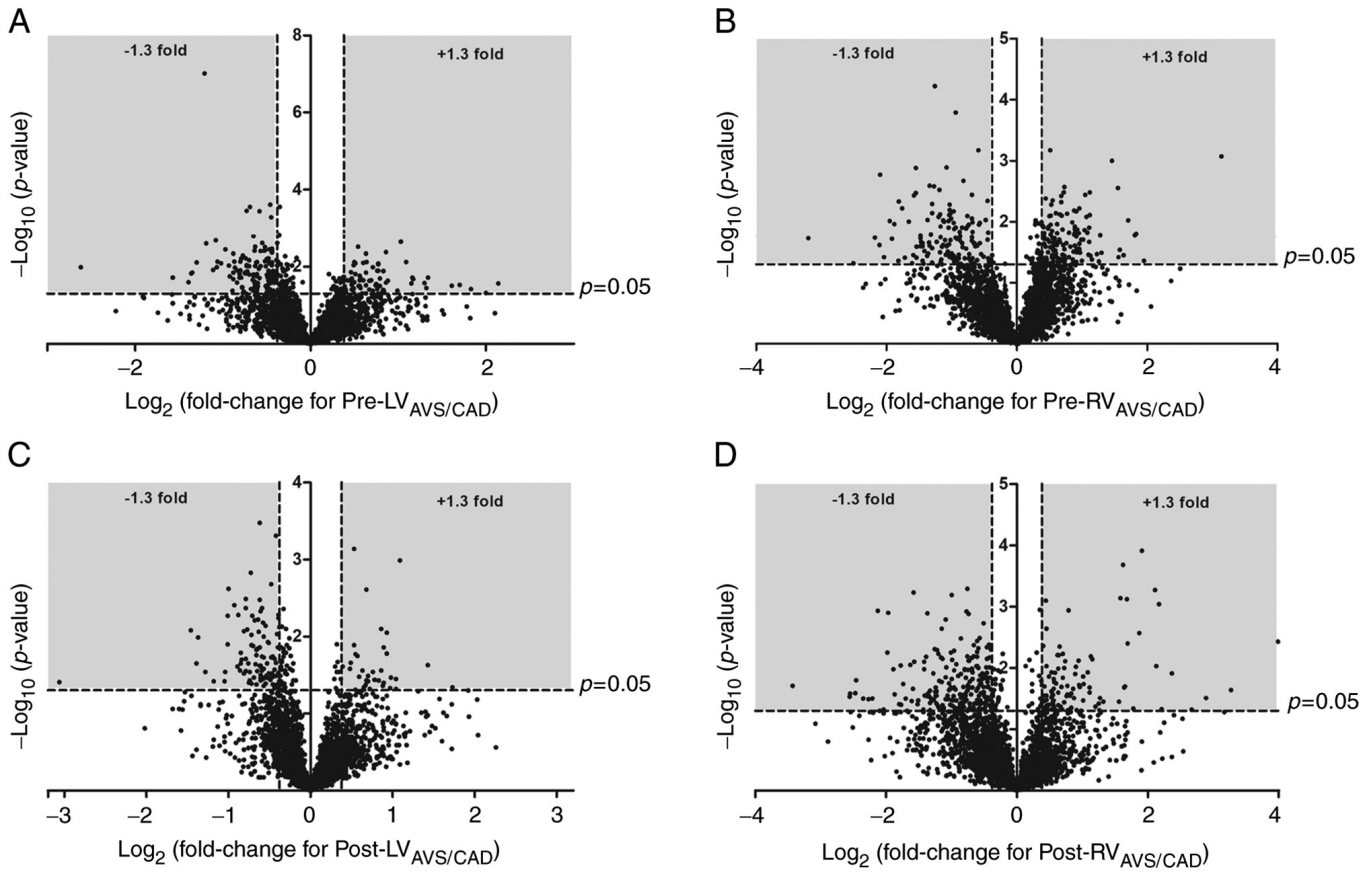
Differences in the proteome of AVS and CAD patients. Volcano plots of the entire sets of proteins quantified before ischaemic cardioplegic arrest (pre) in the LV and RV (A and B, respectively) and after reperfusion (Post) in the LV and RV (C and D, respectively) of AVS patients compared to CAD patients. Each point represents the log2(fold change) between protein levels, plotted against the associated level of statistical significance for the fold change. Proteins in the shaded area (>1.3 or <0.769-fold change, P<0.05) are considered to be differentially expressed. AVS, aortic valve stenosis; CAD, coronary artery disease; RV, right ventricle; LV, left ventricle.

A total of 3,009 proteins were detected in all LV biopsies from AVS (n=6) and CAD (n=6) patients pre-ischaemic cardioplegic arrest while 3,000 were found in all RV biopsies from AVS (n=5) and CAD (n=6) patients pre-ischaemic cardioplegic arrest. In the LV, the expression of 206 proteins were significantly altered (135 with decreased expression in AVS patients relative to CAD patients and 71 with increased expression). The differentially expressed proteins are listed in Table SVIII, and a volcano plot can be found in Fig. 4A. In the RV, the expression of 273 proteins were significantly altered (140 with decreased expression in AVS patients relative to CAD patients and 133 with increased expression; Table SIX and Fig. 4B). This included a significant underexpression of MAP2K1 and MAP2K2 in AVS patients compared to CAD patients. IPA canonical pathway analysis (Table SX) highlighted phospholipase C signalling as the most significantly enriched pathway in the LV (P-value of overlap=4.57×10^−5^); other significantly enriched pathways included Gα_s_ signalling and α-adrenergic signalling. In the RV, several canonical pathways relating to inflammation were enriched, including acute phase response signalling, IL-2 signalling and complement system pathways.

Phosphoproteomic analysis found 24 significantly enriched phosphoproteins in the LV pre-ischaemic cardioplegic arrest comparision (7 with increased expression in AVS patients relative to CAD patients and 17 with decreased expression; Table SXI and Fig. S2A) and 11 significantly enriched phosphoproteins in the RV pre-ischaemic cardioplegic arrest comparision (7 with increased expression in AVS patients relative to CAD patients and 4 with decreased expression; Table SXII and Fig. S2B). Notable differences in the LV include a significant decrease in phosphorylated myosin heavy chains-11 and -9 in the LV of AVS patients compared to CAD patients. IPA canonical signalling pathway analysis (Table SXIII) highlighted several enriched pathways including PKA signalling in both ventricles and cardiac β-adrenergic signalling and calcium signalling in the LV.

### Comparision of protein/phosphoprotein expression in AVS patients compared to CAD patients post-reperfusion

A total of 2,981 proteins were detected in all LV biopsies from AVS (n=5) and CAD (n=5) patients post-reperfusion while 3,218 were found in all RV biopsies from AVS (n=4) and CAD (n=3) patients post-reperfusion. In the LV, the expression of 135 proteins were significantly different between groups (99 with decreased expression in AVS patients relative to CAD patients and 36 with increased expression; Table SXIV and Fig. 4C). In the RV, the expression of 314 proteins were significantly altered (217 with decreased expression in AVS patients relative to CAD patients and 97 with increased expression; Table SXV and Fig. 4D). IPA canonical pathway analysis (Table SXVI) highlighted the significant enrichment of several pathways involved in inflammation (IL-6 and IL-2 signalling in the LV and IL-8 signalling and complement system pathways in the RV) as well as calcium signalling pathways in the RV.

Phosphoproteomic analysis found 19 significantly differentially expressed phosphoproteins in the LV post-reperfusion comparision (4 with increased expression in AVS patients relative to CAD patients and 15 with decreased expression; Table SXVII and Fig. S2C) and 20 significantly differentially expressed phosphoproteins in the RV post-reperfusion comparision (9 with increased expression in AVS patients relative to CAD patients and 11 with decreased expression; Table SXVIII and Fig. S2D). IPA canonical signalling pathway analysis (Table SXIX) highlighted several enriched pathways including PKA signalling, cAMP-mediated signalling and calcium signalling in the LV.

## Discussion

In this study, proteomic analysis involving tandem mass tagging followed by reverse phase nano-liquid chromatography mass spectrometry/mass spectrometry (LC-MS/MS) was utilized to investigate the effect of open heart surgery using cold blood cardioplegic arrest and reperfusion on the changes in protein levels in biopsies collected from left ventricles (LVs) and right venticles (RVs) of patients with either corony artery disease (CAD) or aortic valve stenosis (AVS).

The length of time spent on cross-clamp (ischemic cardioplegic arrest) was significantly longer in AVS patients compared to CAD patients. Surprisingly, despite this difference, CAD patients demonstrated far greater changes in protein expression than AVS patients. This study shows for the first time that the diseased LV of AVS patients shows little change in its proteome during surgery compared to LV of CAD patients. Similarly, the relatively normal RV of AVS patients show little change in its proteome compared to RV proteome of CAD patients which demonstrated the greatest number of changes in protein expression including altered expression of a significant number of mitochondrial proteins.

### Cardioplegic arrest and reperfusion increase proteins of systemic origin in both LV and RV of CAD patients

It is widely assumed that proteins measured in cardiac biopsies are related to the main cell types of the myocardium (e.g. myocytes, smooth muscle cells, fibroblasts, endothelia cells). However, the possibility that some of the proteins have come from blood components (e.g. fragmented platelets, neutrophils) cannot be discounted. This is particularly relevant during reperfusion where neutrophils are known to penetrate the stressed/injured myocardium. Additionally, platelets fragmented by cardiopulmonary bypass would penetrate the vasculature and become attached to the myocardium. We found a significant increase in platelet proteins including integrin α-IIb [which is an important mediator of platelet aggregation via the integrin α(IIb)β(3) complex] and platelet membrane glycoprotein Ib β in the LV of CAD patients post surgery.

It is important to point out that integrins, which are fast turnover focal adhesion proteins, are also found in cardiomyocytes and act to connect the Z-disc with the sarcolemma (17). Mechanical forces including stretching of cardiomyocytes have been shown to markedly increase integrin expression (18). Furthermore β3 integrin has been implicated in cardiac hypertrophy and associated signaling (19–21) and cardiac repair including fibrosis after myocardial infarction (22–24). Unlike β3 integrin, little is known about the role/significance of α2 integrin in cardiomyocytes but it has an important role in cell adhesion and motility, and immune/blood cell regulation when bound to β1 integrin (25).

Other proteins that increase in LV of CAD patients during surgery which are likely to originate outside the myocardium include neutrophil elastase [likely due to activation of neutrophils triggered by cardiopulmonary bypass (26)], coronin-1A [predominantly expressed in leukocytes and important for integrin-mediated leukocyte adhesion (27)] and myeloperoxidase [likely due to its release by neutrophils, and linked to adverse left ventricular remodeling following myocardial ischemia (28)]. Finally, cathelicidin antimicrobial peptide was also increased; cathelicidins are peptides which are expressed at high levels in neutrophils and some epithelia and can act as a natural antibiotic (29). Cathelicidin antimicrobial peptide has been shown to protect cardiomyocytes against ischemia/reperfusion (I/R) injury via activation of survival signaling (30,31).

Similarly, there was also an increase in proteins associated with systemic blood components in the RV of CAD patients during surgery. These included platelet glycoprotein 4, protein disulfide-isomerase A6 (which plays a role in platelet aggregation and activation), and Ras-related protein Ral-A which is abundantly present in human platelets.

### Cardioplegic arrest and reperfusion trigger changes in expression of proteins involved in translation and inflammation in LV and RV of CAD patients

Exposure to CPB and cardioplegic arrest is known to induce inflammation (32), and indeed canonical pathway analysis of the phosphoprotein datasets found a significant enrichment of inflammatory canonical signalling pathways including IL-6, IL-22, IL-17 and acute phase response signalling in the RV and IL-6 and IL-22 in the LV. These changes were due in part to a significant increase in the relative expression of phosphorylated MAPK14 and phosphorylated heat shock protein β-1 (HSPβ1) in both ventricles, and phosphorylated MAPK3 in the LV.

40S ribosomal protein S30 and 60S ribosomal protein L36 were 2 of only 5 proteins to demonstrate decreased expression in the LV of CAD patients following surgery. These are ribonucleoproteins involved in RNA processing and DNA repair (32). Canonical pathway analysis demonstrated that changes in the expression of these ribosomal proteins contributed to significant enrichment of eukaryotic initiation factor (eIF) signalling pathways, which are involved in translation. Protein translation was also affected in the RV, which demonstrated significant increases in the expression of eIF3E, phospho-eIF4B and 60S acidic ribosomal protein P0.

### Cardioplegic arrest and reperfusion trigger changes in mitochondrial proteome consistent with mitochondrial impairment, especially in the RV of CAD patients

The signifcant alteration in mitochondrial proteins in the RV of CAD patients is evident in both the canonical pathway analysis, which lists mitochondrial dysfunction as a highly enriched pathway (Table II), and the Gene Ontology analysis (Fig. 2C). In contrast, the LV of CAD patients shows relatively few changes in the expression of mitochondrial proteins. These differences are unlikley to be due to the disease status as all 6 CAD patients had 3 similar vessel disease (see Materials and methods). Significant dynamic changes occur to the mitochondria during I/R which include swelling and fission which are likely to require additional mitochondrial protein synthesis. Recent research involving paired atrial biopsies collected before and after cardiopulmonary bypass (CPB) with cold cardioplegia for CAD and/or valve surgery has demonstrated that these procedures are associated with mitochondrial DNA damage and biogenesis (33).

A key finding in the present study is the increase in expression of several proteins (NADH dehydrogenase flavoprotein 1, flavoprotein 2, flavoprotein 3, 1 α subcomplex subunit 5, 1 β subcomplex subunit 6, 1 β subcomplex subunit 7) associated with mitochondrial complex I in the RV of CAD patients. In the LV, only flavoprotein 3 demonstrated increased expression. Complex I is the largest and most complicated enzyme of the respiratory chain, known to release deleterious oxygen radicals and whose dysfunction has been linked to a number of hereditary and degenerative diseases (34).

Several proteins increased in the mitochondria in the RV were related to lipid metabolism and the tricarboxylic acid (TCA) cycle. Both delta(3,5)-Delta(2,4)-dienoyl-CoA isomerase and methylmalonyl-CoA epimerase are involved in fatty acid β-oxidation and short-chain fatty acid catabolic process. Proteins associated with the TCA cycle include malate dehydrogenase, isocitrate dehydrogenase and fumarylacetoacetate hydrolase. The latter catalyzes the hydrolysis of 4-fumarylacetoacetate into acetoacetate and fumarate which end up in the TCA cycle, or are used for biosynthetic purposes (35). Signalling relating to the TCA cycle was also highlighted as a significant canonical pathway for the RV (P-value of overlap=4.9×10^−6^).

Other mitochondrial proteins that increased during surgery in the RV include proteins involved in protein biosynthesis (mitochondrial Isoleucine--tRNA ligase), mitochondrial dicarboxylate carrier (solute carrier family 25 member 10) and the optic atrophy 3 protein (OPA3) which is in the inner mitochondrial membrane (36) and implicated in dilated cardiomyopathy (37). The expression of adenylate kinase 2 and ATP synthase protein 8 were also increased in the RV; these proteins play important roles in cellular energy homeostasis and ATP synthesis. In the LV, there was an increase in the expression of mitochondrial calcium uniporter regulator 1 [an activator of the mitochondria Ca^2+^ uniporter that is responsible for mitochondrial calcium uptake (38), a process that is augmented during I/R]. Interestingly, there was also a decrease in the expression of the mitochondrial transmembrane protein 70 (TMEM70) in the LV of CAD patients; TMEM70 facilitates the biogenesis of mammalian F1Fo ATP synthase and knockout of this protein is lethal as it impairs mitochondrial energy production (39).

### Cardioplegic arrest and reperfusion trigger changes in contractile machinary proteins in RV of CAD patients following cardioplegic arrest and reperfusion

There was a decrease in motor proteins (mostly myosin-related) in the RV of CAD patients. These were MYH3 (developmental), MYH7, MYH6 (hypertrophic and dilated cardiomyopathy), myosin light chain 3, unconventional myosin-Ib, F-actin-capping protein subunit β (which plays a role in the regulation of cell morphology and cytoskeletal organization), kinesin-like protein KIF1C (involved in cytoskeleton-dependent intracellular transport) and 39S ribosomal protein L17 (involved in mitochondrial translational elongation and termination). Interestingly, the contractile machinary of the heart appears to be more affected by surgery in the RV compared to the LV. It is difficult to address this issue without having detailed pre- and post-operative functional/structural data using a large number of patients. There have been suggestions that the RV of Coronary artery bypass grafting (CABG) patients selectively sustains more disruptions following CABG surgery (40–42). It is likely that the adaptation to the disease is different between LV and RV and therefore could alter vulnerability to I/R.

### Small number of changes in protein and phosphoprotein expression during cardioplegic arrest and reperfusion in both LV and RV of AVS patients

A major finding in this study was that surgery caused very few changes in the proteome of either ventricle of AVS patients. There was a decrease in the expression of only 3 proteins in the LV (involved in protein cellular transport, fatty acid metabolism and urea synthesis) and altered expression of only 2 proteins in the RV (an extracellular matrix glycoprotein and a protein involved in heme synthesis). The surprising observation that there are far fewer changes in the total protein expression of AVS patients compared to CAD patients suggests that cold ischemic cardioplegic arrest and reperfusion of a heart with hypertrophic LV does not impose significant molecular stress. This is in contrast to the coronary diseased LV and is consistent with proposed strategies to protect the hypertrophic heart (43).

The phosphoproteome showed slightly more significance, with 14 differentially expressed phosphoproteins in the LV and 4 in the RV. Here there are some similarities to the phosphoproteome analysis in CAD patients, with a significant increase in the relative expression of HSPβ1 in both ventricles and a significant increase in the relative expression of MAPK14 in the LV. As with the CAD patients, these findings cause significant enrichment of pro-inflammatory signalling pathways (IL-6 signalling in both ventricles and also IL-17 signialling in the LV).

### Comparison between the proteome/phosphoproteome of the LV and RV of AVS patients compared to CAD patients pre-ischemia and at post reperfusion

The data obtained in this study have been used to update a previous study authored by Littlejohns *et al* (11) in which the pre-ischemic proteomes of the LV and RV of AVS patients were compared with the corresponding ventricles of CAD. In this previous study, approximately 500 proteins were identified in all biopsies. By contrast, in the present study given the advancement of proteomic technology and a larger sample size, we were able to identify approximately 3,000 proteins present in all biopsies for each comparision. Of these detectable proteins, 206 were differentially expressed between AVS and CAD patients in the LV biopsies, and 273 in the RV biopsies taken pre-ischaemic cardioplegic arrest. In the early study only 9 and 73 differentially expressed proteins were detected in the LV and RV biopsies, respectively. Thus, the present study allows us to expore the differences between pathologies in greater detail.

### Differences in adrenergic and calcium signalling pathways between LV and RV of AVS and corresponding LV and RV of CAD patients before ischemia and after reperfusion

IPA canonical pathway analysis of the LV pre-ischaemic cardioplegic arrest samples identified phospholipase C (PLC) signalling as the most significantly enriched canonical pathway. Other enriched pathways included Gα_s_ signalling and α-adrenergic signalling. Similarly, the phosphoprotein analysis also identified several enriched pathways relating to adrenergic signalling (e.g. protein kinase A signalling in both ventricles, and cardiac β-adrenergic signalling in the LV). Calcium signalling was also identified as a significantly enriched canonical pathway in the comparision of the LV phosphoproteome of samples taken pre-ischaemic cardioplegic arrest, driven largely by changes in expression of isoforms of myosin heavy chain such as MYH9 and MYH11, both of which are significantly underexpressed in AVS patients compared to CAD patients. PLC and α- and β-adrenergic signalling pathways are all known to be affected by or implicated in cardiac hypertrophy (44–46), while the expression of myosin heavy chains is also known to be altered in hypertrophic or failing hearts (47,48). It is likely therefore that differences in adrenergic and calcium signalling pathways pre-ischaemic cardioplegic arrest are the result of the different effects of the two pathology types on the basal physiological state of the hearts.

Post-reperfusion, there were also differences in adrenergic and calcium signalling pathways; PKA signalling and cAMP-mediated signalling were identified as significantly enriched phosphoprotein canonical pathways in the post-reperfusion LV samples and calcium signalling was a significantly enriched canonical pathway in both the RV total proteome and the LV phosphoproteome post-reperfusion analysis. These differences may represent a continuation of the basal differences already noted, but may also be affected by different responses of the two types of heart to exposure to cardiopulmonary bypass and cardioplegic arrest.

### Differences in inflammatory pathways between LV and RV of AVS and corresponding LV and RV of CAD patients before ischemia and after reperfusion

Differences in inflammatory pathways also exist between the two pathologies. In the RV pre-ischaemic cardioplegic arrest biopsies the most significantly enriched canonical pathway was acute phase response signalling (a group of proteins which respond to inflamation); IL-2 signalling (a pro-inflammatory cytokine) and complement system pathways were also significantly enriched. These effects were in part due to increased expression of MAP2K1 and MAP2K2 in the RV pre-ischaemic cardioplegic arrest samples from CAD patients compared to AVS patients. Differences in basal levels of inflammation are likely to exist between CAD and AVS hearts-for example one study demonstrated increased systemic levels of pro-inflammatory IL-3 in patients with CAD (49).

There are also differences in inflammatory pathways between AVS and CAD hearts post-reperfusion. In the LV, there was significant enrichment of IL-6 and IL-2 canonical signalling pathways while in the RV there was significant enrichment of IL-8 signalling as well as complement system pathways. As previously mentioned, exposure to CPB and cardioplegic arrest is known to induce inflammation (32); our results suggest that the degree of inflammation was different between patients with different pathologies. This may relate to the fact that the cross-clamp time was longer in the AVS group, but may also reflect an influence of the underlying pathology on the inflammatory response to surgery.

In conclusion, the study presented in this manuscript demonstrates for the first time that LVs and RVs of patients with either AVS or CAD respond differently at the molecular level to cold ischemic cardioplegic arrest and reperfusion. These molecular changes are disease-dependent, with CAD patients showing a larger number of proteins changing during the surgery despite having shorter cardioplegic arrest; notable changes include a higher number of differentially expressed mitochondrial proteins and lower number of contractile proteins in the RV of CAD patients. Enrichment analysis indicates involvement of canonical pathways associated with inflammation, mitochondria and contractility. In addition to intra-disease changes, each ventriclular chamber showed inter-disease differences measured before and after cardioplegic arrest. It is important to note that this is largely a descriptive study designed to report the changes in the proteome/phosphoproteome in different ventricular chambers of two groups of patients with different pathologies. Further research is needed to identify the mechanisms involved and to investigate the significance of these changes. In particular, future research could investigate the relevant functional impairment and differences between sub-groups of patients, like those with differing degrees of CAD, sex differences and the presence of co-morbidities.

## Supplementary Data



## Figures and Tables

**Table I:** Key patient characteristics and intraoperative data.

Characteristics	CAD (n=6)	AVS (n=6)
Mean age, years (range)	72±3 (60–80)	72±2 (65–77)
Sex (M/F)	4/2	4/2
NYHA	I: 3	II: 3
	II: 3	III: 3
Diabetes	4	2
Hypertension	5	4
Cross clamp time (min)	47.5±5.3	75.5±7.1^a^
Bypass time (min)	86.8±7.1	112.3±9.2
		(P=0.053)

a Represents a statistically significant difference (P<0.05, Student's unpaired t-test). CAD, coronary artery disease; AVS, aortic valve stenosis.

**Table II:** Enriched canonical pathways for the total protein analysis of the LV and RV of CAD patients.

Ventricle	Ingenuity canonical pathway	P-value of overlap	Molecules
LV	Airway pathology in chronic obstructive pulmonary disease	0.004	ELANE, MPO
	Melatonin degradation III	0.005	MPO
	Regulation of eIF4 and p70S6K signalling	0.026	AGO1, FAU, ITGA2B
	Airway inflammation in asthma	0.030	ELANE
	EIF2 signalling	0.045	AGO1, FAU, RPL36
RV	Mitochondrial dysfunction	1.0×10^−11^	ATP5PD, COX7A2L, CYB5A, CYC1, MAOA, MAOB, MAPK9, NDUFA10, NDUFA5, NDUFA7, NDUFB1, NDUFB10, NDUFB2, NDUFB6, NDUFB7, NDUFS8, NDUFV1, NDUFV2, NDUFV3, UQCRQ
	Oxidative phosphorylation	1.3×10^−11^	ATP5PD, COX7A2L, CYB5A, CYC1, NDUFA10, NDUFA5, NDUFA7, NDUFB1, NDUFB10, NDUFB2, NDUFB6, NDUFB7, NDUFS8, NDUFV1, NDUFV2, NDUFV3, UQCRQ
	Sirtuin signalling pathway	1.4×10^−6^	CYC1, NDUFA10, NDUFA5, NDUFA7, NDUFB1, NDUFB10, NDUFB2, NDUFB6, NDUFB7, NDUFS8, NDUFV1, NDUFV2, NDUFV3, PARP1, PPIF, SIRT2
	Estrogen receptor signalling	4.4×10^−6^	CYC1, MYL3, MYL4, MYL7, NDUFA10, NDUFA5, NDUFA7, NDUFB1, NDUFB10, NDUFB2, NDUFB6, NDUFB7, NDUFS8, NDUFV1, NDUFV2, NDUFV3, RALA
	TCA cycle II (eukaryotic)	4.9×10^−6^	CS, DLST, FH, IDH3A, IDH3G, SUCLG1
	Serotonin receptor signalling	1.6×10^−5^	MAOA, MAOB, QDPR, SPR
	Glucocorticoid receptor signalling	2.2×10^−5^	CYC1, FGG, HSPA5, MAPK9, NDUFA10, NDUFA5, NDUFA7, NDUFB1, NDUFB10, NDUFB2, NDUFB6, NDUFB7, NDUFS8, NDUFV1, NDUFV2, NDUFV3, RALA
	Agranulocyte adhesion and diapedesis	4.7×10^−5^	MYH3, MYH6, MYH7, MYH9, MYL3, MYL4, MYL7, PODXL
	Calcium signalling	1.6×10^−4^	MYH3, MYH6, MYH7, MYH9, MYL3, MYL4, MYL7, TNNI1, TRDN
	Melatonin degradation II	5.1×10^−4^	MAOA, MAOB
	Hepatic fibrosis/hepatic stellate cell activation	9.5×10^−4^	MYH3, MYH6, MYH7, MYH9, MYL3, MYL4, MYL7
	Dilated cardiomyopathy signalling pathway	1.0×10^−3^	MYH3, MYH6, MYH7, MYH9, MYL3, MYL4, MYL7, TNNI1
	Cellular effects of sildenafil (Viagra)	1.4×10^−3^	MYH3, MYH6, MYH7, MYH9, MYL3, MYL4, MYL7
	ILK signalling	1.5×10^−3^	MAPK9, MYH3, MYH6, MYH7, MYH9, MYL3, MYL4, MYL7, RHOT1
	Dopamine receptor signalling	1.9×10^−3^	MAOA, MAOB, PPP1R14C, QDPR, SPR
	Tight junction signalling	2.0×10^−3^	MYH3, MYH6, MYH7, MYH9, MYL3, MYL4, MYL7, VAPA
	Gα12/13 signalling	2.6×10^−3^	F2, MAPK9, MYL3, MYL4, MYL7, RALA
	Actin cytoskeleton signalling	6.3×10^−3^	F2, MYH3, MYH6, MYH7, MYH9, MYL3, MYL4, MYL7, RALA
	RHOGDI signalling	6.8×10^−3^	MYH3, MYH6, MYH7, MYH9, MYL3, MYL4, MYL7, RHOT1
	Phenylalanine degradation IV (mammalian, via side chain)	1.3×10^−2^	MAOA, MAOB

All significant pathways are shown for LV while for the RV only the top 20 most significant are shown. CAD, coronary artery disease; RV, right ventricle; LV, left ventricle.

**Table III:** Enriched canonical pathways for the relative phosphoprotein analysis of the LV and RV of CAD patients.

Ventricle	Ingenuity canonical pathway	P-value of overlap	Molecules
LV	Endocannabinoid cancer inhibition pathway	7.08E-06	MAPK14, MAPK3, PRKAR1A, VIM
	Amyloid processing	1.23E-05	MAPK14, MAPK3, PRKAR1A
	Sertoli cell-sertoli cell junction signalling	3.02E-05	MAPK14, MAPK3, PRKAR1A, SPTBN1
	Melatonin signalling	3.47E-05	MAPK3, PRKAR1A, SLC2A4
	Apelin adipocyte signalling pathway	5.89E-05	MAPK14, MAPK3, PRKAR1A
	BMP signalling pathway	6.17E-05	MAPK14, MAPK3, PRKAR1A
	Cardiac hypertrophy signalling	7.24E-05	HSPB1, MAPK14, MAPK3, PRKAR1A
	Insulin secretion signalling pathway	8.32E-05	MAPK14, MAPK3, PRKAR1A, SLC2A4
	Antioxidant action of vitamin C	1.26E-04	MAPK14, MAPK3, SLC2A4
	CDK5 signalling	1.29E-04	MAPK14, MAPK3, PRKAR1A
	Renin-angiotensin signalling	1.58E-04	MAPK14, MAPK3, PRKAR1A
	Gαs signalling	1.62E-04	ADD1, MAPK3, PRKAR1A
	Endocannabinoid developing neuron pathway	1.70E-04	MAPK14, MAPK3, PRKAR1A
	IL-6 signalling	1.95E-04	HSPB1, MAPK14, MAPK3
	IL-22 signalling	2.04E-04	MAPK14, MAPK3
	Role of JAK family kinases in IL-6-type cyto kine signalling	2.24E-04	MAPK14, MAPK3
	IL-17A signalling in gastric cells	2.40E-04	MAPK14, MAPK3
	Insulin receptor signalling	2.51E-04	MAPK3, PRKAR1A, SLC2A4
	Dilated cardiomyopathy signalling pathway	2.82E-04	MAPK14, MAPK3, PRKAR1A
	Endocannabinoid neuronal synapse pathway	2.88E-04	MAPK14, MAPK3, PRKAR1A
RV	p38 MAPK signalling	0.001	HSPB1, MAPK14
	IL-6 signalling	0.001	HSPB1, MAPK14
	Acute phase response signalling	0.003	HNRNPK, MAPK14
	Cardiac hypertrophy signalling	0.005	HSPB1, MAPK14
	Parkinson's signalling	0.007	MAPK14
	IL-22 signalling	0.010	MAPK14
	Role of JAK family kinases in IL-6-type cytokine signalling	0.010	MAPK14
	IL-17A signalling in gastric cells	0.011	MAPK14
	4-1BB signalling in T lymphocytes	0.014	MAPK14
	Inhibition of angiogenesis by TSP1	0.014	MAPK14
	IL-17A signalling in fibroblasts	0.016	MAPK14
	April mediated signalling	0.018	MAPK14
	B cell activating factor signalling	0.018	MAPK14
	nNOS signalling in skeletal muscle cells	0.020	SNTA1
	iNOS signalling	0.020	MAPK14
	Cardiac hypertrophy signalling (enhanced)	0.021	HSPB1, MAPK14
	Amyloid processing	0.021	MAPK14
	UVC-induced MAPK signalling	0.021	MAPK14
	UVB-induced MAPK signalling	0.022	MAPK14
	EGF signalling	0.023	MAPK14

Only the top 20 most significant pathways are shown for each ventricle. CAD, coronary artery disease; LV, left ventricle; RV, right ventricle.

**Table IV:** Enriched canonical pathways for the relative phosphoprotein analysis of the LV and RV of AVS patients.

Ventricle	Ingenuity canonical pathway	P-value of overlap	Molecules
LV	Dilated cardiomyopathy signalling pathway	4.90E-05	GSK3B, MAPK14, TTN
	Adrenomedullin signalling pathway	1.23E-04	GSK3B, MAPK14, TTN
	IL-17A signalling in fibroblasts	1.66E-04	GSK3B, MAPK14
	Cardiac hypertrophy signalling	2.63E-04	GSK3B, HSPB1, MAPK14
	Amyloid processing	2.95E-04	GSK3B, MAPK14
	IL-17A signalling in airway cells	5.13E-04	GSK3B, MAPK14
	IL-7 signalling pathway	6.92E-04	GSK3B, MAPK14
	ERBB signalling	1.00E-03	GSK3B, MAPK14
	ATM signalling	1.07E-03	HP1BP3, MAPK14
	p53 signalling	1.10E-03	GSK3B, MAPK14
	Hepatic fibrosis signalling pathway	1.10E-03	GSK3B, MAPK14, TTN
	Mouse embryonic stem cell pluripotency	1.23E-03	GSK3B, MAPK14
	p38 MAPK signalling	1.58E-03	HSPB1, MAPK14
	Endocannabinoid developing neuron pathway	1.74E-03	GSK3B, MAPK14
	IL-6 signalling	1.86E-03	HSPB1, MAPK14
	Cardiac hypertrophy signalling (enhanced)	2.24E-03	GSK3B, HSPB1, MAPK14
	Endocannabinoid cancer inhibition pathway	2.29E-03	GSK3B, MAPK14
	Factors promoting cardiogenesis in vertebrates	2.57E-03	GSK3B, MAPK14
	WNT/β-catenin signalling	3.39E-03	APPL1, GSK3B
	IL-17 signalling	3.89E-03	GSK3B, MAPK14
RV	Aldosterone signalling in epithelial cells	0.000	CRYAB, HSPB1
	Protein ubiquitination pathway	0.001	CRYAB, HSPB1
	Death receptor signalling	0.020	HSPB1
	p38 MAPK signalling	0.025	HSPB1
	Ferroptosis signalling pathway	0.026	HSPB1
	IL-6 signalling	0.027	HSPB1
	Aryl hydrocarbon receptor signalling	0.033	HSPB1
	ERK/MAPK signalling	0.045	HSPB1

Only the top 20 most significant pathways are shown for the LV while for the RV all significant pathways are shown. AVS, aortic valve stenosis; LV, left ventricle; RV, right ventricle.

## Data Availability

The datasets used and/or analysed during the current study are publically available at Open Science Framework (https://doi.org/10.17605/OSF.IO/BPJGX).

## Abbreviations

AVS: aortic valve stenosis
CAD: coronary artery disease
FC: fold change
GO: Gene Ontology
IPA: Ingenuity Pathway Analysis
LC-MS/MS: liquid chromatography mass spectrometry/mass spectrometry
LV: left ventricle
RV: right ventricle
TMT: tandem mass tags
TBS: Tris-buffered saline
SEM: standard error of the mean
