# Supplementary material for: Effect of cardioplegic arrest and reperfusion on left and right ventricular proteome/phosphoproteome in patients undergoing surgery for coronary or aortic valve disease
Source: Int J Mol Med. 2022 Apr 14;49(6):77. doi: 10.3892/ijmm.2022.5133 (PMC9083849; doi:10.3892/ijmm.2022.5133)

Figure S1. Volcano plots for relative phosphoprotein expression (post vs. pre). Volcano plots of the relative phosphoprotein expression, quantified before ischemic cardioplegic arrest (pre) and after reperfusion (post) in LV and RV of CAD patients (A and B, respectively) and LV and RV of AVS patients (C and D, respectively). Each point represents the log2(fold change) between pre and post phosphoprotein levels, plotted against the associated level of statistical significance for the fold change. Proteins in the shaded area (>1.3 or <0.769-fold change, P<0.05) are considered to be differentially expressed. LV, left ventricle; RV, right ventricle; CAD, coronary artery disease; AVS, aortic artery stenosis.

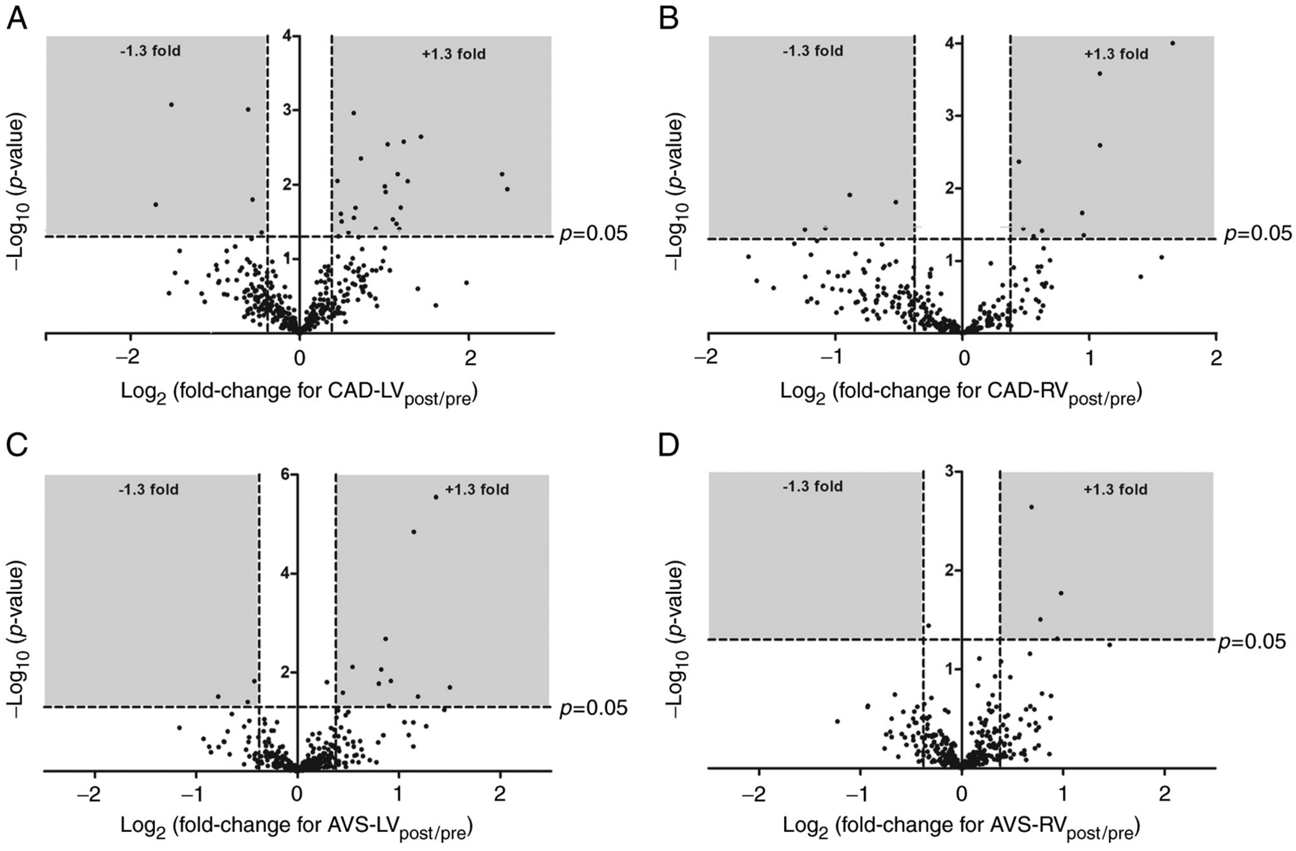

Figure S2. Volcano plots for relative phosphoprotein expression (AVS vs. CAD). Volcano plots of the differences in relative phosphoprotein expression between AVS and CAD patients before ischaemic cardioplegic arrest (pre) in the LV and RV (A and B, respectively) and post ischaemic cardioplegic arrest (post) in the LV and RV (C and D, respectively). Each point represents the log2(fold change) between relative phosphoprotein levels, plotted against the associated level of statistical significance for the fold change. Proteins in the shaded area (>1.3 or <0.769-fold change, P<0.05) are considered to be differentially expressed. LV, left ventricle; RV, right ventricle; CAD, coronary artery disease; AVS, aortic artery stenosis.

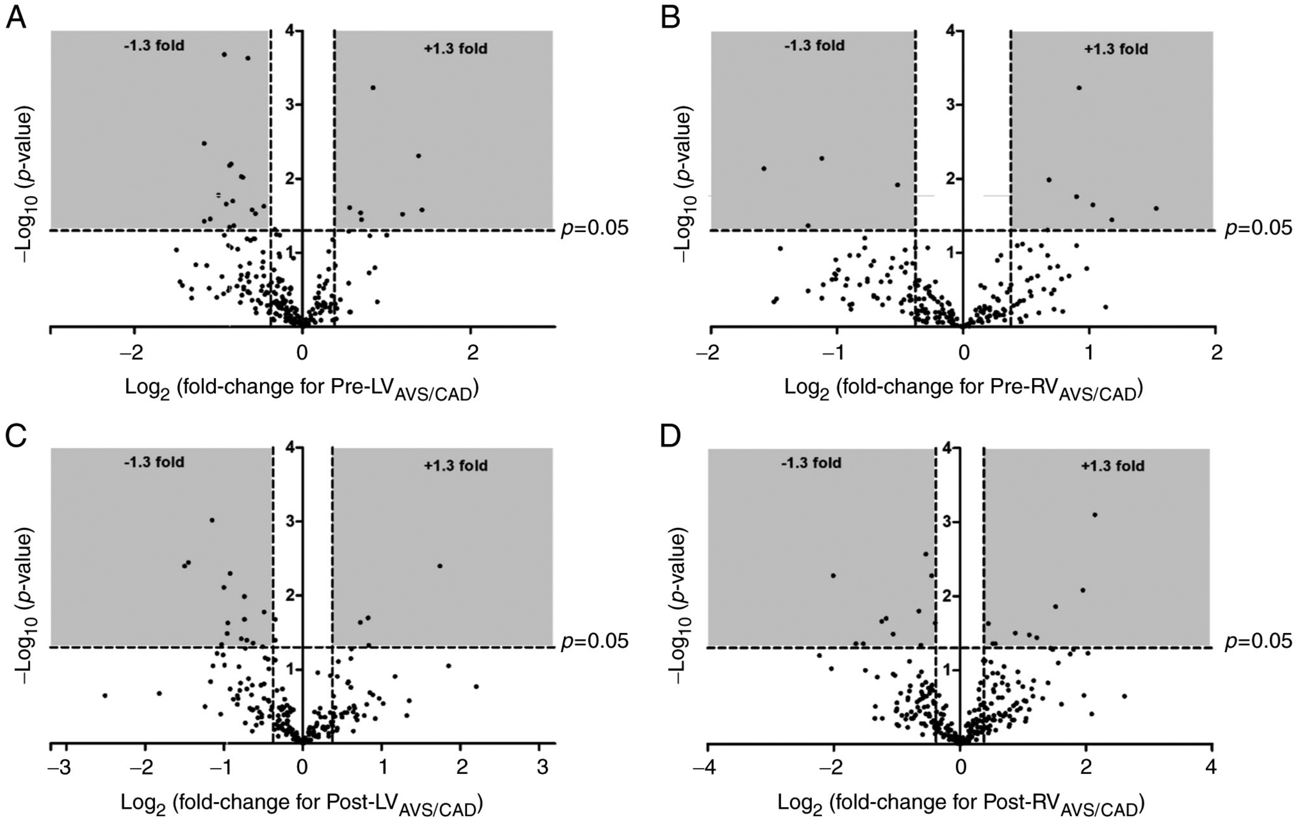

Supplement: Supplementary file 1 [file Supplementary_Data1.pdf]
