# Supplementary material for: Effect of cardioplegic arrest and reperfusion on left and right ventricular proteome/phosphoproteome in patients undergoing surgery for coronary or aortic valve disease
Source: Int J Mol Med. 2022 Apr 14;49(6):77. doi: 10.3892/ijmm.2022.5133 (PMC9083849; doi:10.3892/ijmm.2022.5133)
Supplement: Supplementary file 2 [file Supplementary_Data2.pdf]

Table SI. Differentially expressed proteins from LV of CAD patients.

Accession no.	Gene name	Description	Fold change	P-value
Q96S52	*PIGS*	GPI transamidase component PIG-S (Phosphatidylinositol-glycan biosynthesis class S protein)	2.09	0.001
P05164	*MPO*	Myeloperoxidase (MPO) (EC 1.11.2.2) [Cleaved into: Myeloperoxidase; 89 kDa myeloperoxidase; 84 kDa myeloperoxidase; Myeloperoxidase light chain; Myeloperoxidase heavy chain]	2.53	0.012
P11169	*SLC2A3*	Solute carrier family 2, facilitated glucose transporter member 3 (Glucose transporter type 3, brain) (GLUT-3)	3.17	0.014
Q5TBA9	*FRY*	Protein furry homolog	1.33	0.018
P62861	*FAU*	40S ribosomal protein S30 (Small ribosomal subunit protein eS30)	0.35	0.019
P49913	*CAMP*	Cathelicidin antimicrobial peptide (18 kDa cationic antimicrobial protein) (CAP-18) (hCAP-18) [Cleaved into: Antibacterial peptide FALL-39 (FALL-39 peptide antibiotic); Antibacterial peptide LL-37]	2.78	0.023
W8QEY1		Lactotransferrin	1.96	0.025
Q86UX7	*FERMT3*	Fermitin family homolog 3 (Kindlin-3) (MIG2-like protein) (Unc-112-related protein 2)	4.09	0.027
Q08J23	*NSUN2*	RNA cytosine C(5)-methyltransferase NSUN2 (EC 2.1.1.-) (Myc-induced SUN domain-containing protein) (Misu) (NOL1/NOP2/Sun domain family member 2) (Substrate of AIM1/Aurora kinase B) (mRNA cytosine C(5)-methyltransferase) (EC 2.1.1.-) (tRNA cytosine C(5)-methyltransferase) (EC 2.1.1.-) (EC 2.1.1.203) (tRNA methyltransferase 4 homolog) (hTrm4)	1.53	0.029
P31146	*CORO1A*	Coronin-1A (Coronin-like protein A) (Clipin-A) (Coronin-like protein p57) (Tryptophan aspartate-containing coat protein) (TACO)	1.69	0.032
Q9Y383	*LUC7L2*	Putative RNA-binding protein Luc7-like 2	0.73	0.032
P56181	*NDUFV3*	NADH dehydrogenase [ubiquinone] flavoprotein 3, mitochondrial (Complex I-9kD) (CI-9kD) (NADH-ubiquinone oxidoreductase 9 kDa subunit) (Renal carcinoma antigen NY-REN-4)	1.35	0.035
P61626	*LYZ*	Lysozyme C (EC 3.2.1.17) (1,4-beta-N-acetylmuramidase C)	1.79	0.036
P08514	*ITGA2B*	Integrin alpha-IIb (GPalpha IIb) (GPIIb) (Platelet membrane glycoprotein IIb) (CD antigen CD41) [Cleaved into: Integrin alpha-IIb heavy chain; Integrin alpha-IIb light chain, form 1; Integrin alpha-IIb light chain, form 2]	5.32	0.037

Q9Y3U8	*RPL36*	60S ribosomal protein L36 (Large ribosomal subunit protein eL36)	0.48	0.038
Q9BUB7	*TMEM70*	Transmembrane protein 70, mitochondrial	0.73	0.038
P02663	*CSN1S2*	Alpha-S2-casein [Cleaved into: Casocidin-1 (Casocidin-I)]	0.17	0.039
A0A024R9Q1	*THBS1*	Thrombospondin 1, isoform CRA_a	2.59	0.040
P13224	*GP1BB*	Platelet glycoprotein Ib beta chain (GP-Ib beta) (GPIb-beta) (GPIbB) (Antigen CD42b-beta) (CD antigen CD42c)	4.51	0.041
Q5JSH3	*WDR44*	WD repeat-containing protein 44 (Rabphilin-11)	1.79	0.042
Q96AQ8	*MCUR1*	Mitochondrial calcium uniporter regulator 1 (MCU regulator 1) (Coiled-coil domain-containing protein 90A, mitochondrial)	1.32	0.043
A7MD96	*SYNPO*	SYNPO protein (Fragment)	1.40	0.048
A0A5C2GDN1		IGH + IGL c107_heavy_IGHV3-74_IGHD7-27_IGHJ4 (Fragment)	1.33	0.049
P08246	*ELANE*	Neutrophil elastase (EC 3.4.21.37) (Bone marrow serine protease) (Elastase-2) (Human leukocyte elastase) (HLE) (Medullasin) (PMN elastase)	3.34	0.049

Proteins differentially (fold change >1.3 or <0.769) and significantly (P<0.05) expressed in post vs. pre samples in the LV of CAD patients. LV, left ventricle; CAD, coronary artery disease.

Table SII. Differentially expressed phosphoproteins from LV of CAD patients.					
Accession no.	*Gene name*	Description	Phosphosite	Fold change	P-value
Q6P5Q4	*LMOD2*	Leiomodin-2 (Cardiac leiomodin) (C-LMOD) (Leiomodin)	S10(Phospho) T2(Phospho)	0.35	0.001
B4DUQ1		Heterogeneous nuclear ribonucleoprotein K	S6(Phospho)	0.65	0.001
Q13424	*SNTA1*	Alpha-1-syntrophin (59 kDa dystrophin-associated protein A1 acidic component 1) (Pro-TGF-alpha cytoplasmic domain-interacting protein 1) (TACIP1) (Syntrophin-1)	S3(Phospho) S4(Phospho)	1.56	0.001
P04792	*HSPB1*	Heat shock protein beta-1 (HspB1) (28 kDa heat shock protein) (Estrogen-regulated 24 kDa protein) (Heat shock 27 kDa protein) (HSP 27) (Stress-responsive protein 27) (SRP27)	S3(Phospho)	2.70	0.002
P14672	*SLC2A4*	Solute carrier family 2, facilitated glucose transporter member 4 (Glucose transporter type 4, insulin-responsive) (GLUT-4)	Ambiguous	2.35	0.003
E9PAV3	*NACA*	Nascent polypeptide-associated complex subunit alpha, muscle-specific form (Alpha-NAC, muscle-specific form) (skNAC)	S12(Phospho)	2.05	0.003
P08670	*VIM*	Vimentin	S6(Phospho)	1.65	0.004
P27361	*MAPK3*	Mitogen-activated protein kinase 3 (MAP kinase 3) (MAPK 3) (EC 2.7.11.24) (ERT2) (Extracellular signal-regulated kinase 1) (ERK-1) (Insulin-stimulated MAP2 kinase) (MAP kinase isoform p44) (p44-MAPK) (Microtubule-associated protein 2 kinase) (p44-ERK1)	T13(Phospho) Y15(Phospho)	5.25	0.007
Q16539	*MAPK14*	Mitogen-activated protein kinase 14 (MAP kinase 14) (MAPK 14) (EC 2.7.11.24) (Cytokine suppressive anti-inflammatory drug-binding protein) (CSAID-binding protein) (CSBP) (MAP	T7(Phospho) Y9(Phospho)	2.23	0.007

		kinase MXI2) (MAX-interacting protein 2) (Mitogen-activated protein kinase p38 alpha) (MAP kinase p38 alpha) (Stress-activated protein kinase 2a) (SAPK2a)			
Q86TC9	*MYPN*	Myopalladin (145 kDa sarcomeric protein)	S3(Phospho) Ambiguous	1.36	0.009
E9PAV3	*NACA*	Nascent polypeptide-associated complex subunit alpha, muscle-specific form (Alpha-NAC, muscle-specific form) (skNAC)	S12(Phospho)	2.42	0.009
Q9H987	*SYNPO2L*	Synaptopodin 2-like protein	S14(Phospho)	2.01	0.010
P02511	*CRYAB*	Alpha-crystallin B chain (Alpha(B)-crystallin) (Heat shock protein beta-5) (HspB5) (Renal carcinoma antigen NY-REN-27) (Rosenthal fiber component)	S3(Phospho)	5.48	0.012
Q09666	*AHNAK*	Neuroblast differentiation-associated protein AHNAK (Desmoyokin)	S3(Phospho)	2.02	0.013
Q9UKG1	*APPL1*	DCC-interacting protein 13-alpha (Dip13-alpha) (Adapter protein containing PH domain, PTB domain and leucine zipper motif 1)	S14(Phospho)	0.68	0.016
Q6P5Q4	*LMOD2*	Leiomodin-2 (Cardiac leiomodin) (C-LMOD) (Leiomodin)	S10(Phospho) Y8(Phospho)	0.31	0.019
E9PAV3	*NACA*	Nascent polypeptide-associated complex subunit alpha, muscle-specific form (Alpha-NAC, muscle-specific form) (skNAC)	S13(Phospho)	2.29	0.020
E9PAV3	*NACA*	Nascent polypeptide-associated complex subunit alpha, muscle-specific form (Alpha-NAC, muscle-specific form) (skNAC)	S12(Phospho)	1.58	0.020
P35611	*ADD1*	Alpha-adducin (Erythrocyte adducin subunit alpha)	S4(Phospho)	1.40	0.025
A0A0A0MRL6	*ABLIM1*	Actin-binding LIM protein 1	T1(Phospho)	1.56	0.028
Q14247	*CTTN*	Src substrate cortactin (Amplaxin) (Oncogene EMS1)	S9(Phospho) T5(Phospho)	2.14	0.029
E9PAV3	*NACA*	Nascent polypeptide-associated complex subunit alpha, muscle-specific form (Alpha-NAC, muscle-specific form) (skNAC)	S12(Phospho)	1.41	0.031

P10644	*PRKAR1A*	cAMP-dependent protein kinase type I-alpha regulatory subunit (Tissue-specific extinguisher 1) (TSE1)	S13(Phospho) S7(Phospho)	2.21	0.034
A0A0S2Z530	*LDB3*	LIM domain binding 3 isoform 1 (LIM domain binding 3, isoform CRA_h) (Fragment)	S5(Phospho)	1.86	0.039
B4DVR4		cDNA FLJ60912, highly similar to Vinexin	S3(Phospho) S4(Phospho) Ambiguous	2.26	0.040
E9PAV3	*NACA*	Nascent polypeptide-associated complex subunit alpha, muscle-specific form (Alpha-NAC, muscle-specific form) (skNAC)	S4(Phospho)	1.49	0.044
O00505	*KPNA3*	Importin subunit alpha-4 (Importin alpha Q2) (Qip2) (Karyopherin subunit alpha-3) (SRP1-gamma)	S11(Phospho)	0.73	0.044
Q01082	*SPTBN1*	Spectrin beta chain, non-erythrocytic 1 (Beta-II spectrin) (Fodrin beta chain) (Spectrin, non-erythroid beta chain 1)	S3(Phospho) S7(Phospho)	1.37	0.049

Phosphoproteins whose expression relative to total protein expression is differentially (fold change >1.3 or <0.769) and significantly (P<0.05) expressed in post vs pre samples from the LV of CAD patients. LV, left ventricle; CAD, coronary artery disease.

Table SIII. Differentially expressed proteins from RV of CAD patients.				
Accession no.	Gene name	Description	Fold change	P-value
Q9P0P8	*MTRES1*	Mitochondrial transcription rescue factor 1	2.17	0.000
P11055	*MYH3*	Myosin-3 (Muscle embryonic myosin heavy chain) (Myosin heavy chain 3) (Myosin heavy chain, fast skeletal muscle, embryonic) (SMHCE)	0.16	0.000
P60228	*EIF3E*	Eukaryotic translation initiation factor 3 subunit E (eIF3e) (Eukaryotic translation initiation factor 3 subunit 6) (Viral integration site protein INT-6 homolog) (eIF-3 p48)	1.34	0.001
P02679	*FGG*	Fibrinogen gamma chain	0.57	0.002
Q4VC31	*MIX23*	Protein MIX23 (Coiled-coil domain-containing protein 58)	2.15	0.002
P12883	*MYH7*	Myosin-7 (Myosin heavy chain 7) (Myosin heavy chain slow isoform) (MyHC-slow) (Myosin heavy chain, cardiac muscle beta isoform) (MyHC-beta)	0.41	0.003
P47756	*CAPZB*	F-actin-capping protein subunit beta (CapZ beta)	0.70	0.003
O60669	*SLC16A7*	Monocarboxylate transporter 2 (MCT 2) (Solute carrier family 16 member 7)	1.35	0.003
P56181	*NDUFV3*	NADH dehydrogenase [ubiquinone] flavoprotein 3, mitochondrial (Complex I-9kD) (CI-9kD) (NADH-ubiquinone oxidoreductase 9 kDa subunit) (Renal carcinoma antigen NY-REN-4)	2.09	0.005
Q02487	*DSC2*	Desmocollin-2 (Cadherin family member 2) (Desmocollin-3) (Desmosomal glycoprotein II) (Desmosomal glycoprotein III)	1.42	0.005
Q9H1E3	*NUCKS1*	Nuclear ubiquitous casein and cyclin-dependent kinase substrate 1 (P1)	1.65	0.006
Q15084	*PDIA6*	Protein disulfide-isomerase A6 (EC 5.3.4.1) (Endoplasmic reticulum protein 5) (ER protein 5) (ERp5) (Protein disulfide isomerase P5) (Thioredoxin domain-containing protein 7)	1.44	0.006
Q9H6K4	*OPA3*	Optic atrophy 3 protein	1.47	0.007
P17568	*NDUFB7*	NADH dehydrogenase [ubiquinone] 1 beta subcomplex subunit 7 (Cell adhesion protein SQM1) (Complex I-B18) (CI-B18) (NADH-ubiquinone oxidoreductase B18 subunit)	1.40	0.007

P54819	*AK2*	Adenylate kinase 2, mitochondrial (AK 2) (EC 2.7.4.3) (ATP-AMP transphosphorylase 2) (ATP:AMP phosphotransferase) (Adenylate monophosphate kinase) [Cleaved into: Adenylate kinase 2, mitochondrial, N-terminally processed]	1.46	0.008
Q12908	*SLC10A2*	Ileal sodium/bile acid cotransporter (Apical sodium-dependent bile acid transporter) (ASBT) (Ileal Na(+)/bile acid cotransporter) (Ileal sodium-dependent bile acid transporter) (IBAT) (ISBT) (Na(+)-dependent ileal bile acid transporter) (Sodium/taurocholate cotransporting polypeptide, ileal) (Solute carrier family 10 member 2)	0.59	0.008
Q96BQ5	*CCDC127*	Coiled-coil domain-containing protein 127	1.41	0.008
P08590	*MYL3*	Myosin light chain 3 (Cardiac myosin light chain 1) (CMLC1) (Myosin light chain 1, slow-twitch muscle B/ventricular isoform) (MLC1SB) (Ventricular myosin alkali light chain) (Ventricular myosin light chain 1) (VLCl) (Ventricular/slow twitch myosin alkali light chain) (MLC-lV/sb)	0.53	0.009
Q08426	*EHHADH*	Peroxisomal bifunctional enzyme (PBE) (PBFE) (L-bifunctional protein) (LBP) (Multifunctional enzyme 1) (MFE1) [Includes: Enoyl-CoA hydratase/3,2-trans-enoyl-CoA isomerase (EC 4.2.1.17) (EC 5.3.3.8); 3-hydroxyacyl-CoA dehydrogenase (EC 1.1.1.35)]	1.49	0.009
P50213	*IDH3A*	Isocitrate dehydrogenase [NAD] subunit alpha, mitochondrial (EC 1.1.1.41) (Isocitric dehydrogenase subunit alpha) (NAD(+)-specific ICDH subunit alpha)	1.47	0.009
P40926	*MDH2*	Malate dehydrogenase, mitochondrial (EC 1.1.1.37)	1.41	0.010
P52948	*NUP98*	Nuclear pore complex protein Nup98-Nup96 (EC 3.4.21.-) [Cleaved into: Nuclear pore complex protein Nup98 (98 kDa nucleoporin) (Nucleoporin Nup98) (Nup98); Nuclear pore complex protein Nup96 (96 kDa nucleoporin) (Nucleoporin Nup96) (Nup96)]	1.54	0.010
B1AK88	*CAPZB*	F-actin-capping protein subunit beta	0.62	0.011
O00763	*ACACB*	Acetyl-CoA carboxylase 2 (EC 6.4.1.2) (ACC-beta)	0.28	0.011
O95139	*NDUFB6*	NADH dehydrogenase [ubiquinone] 1 beta subcomplex subunit 6 (Complex I-B17) (CI-B17) (NADH-ubiquinone oxidoreductase B17 subunit)	1.57	0.012
C9JWC3	*SORBS2*	Sorbin and SH3 domain-containing protein 2 (Fragment)	1.89	0.012

O43768	*ENSA*	Alpha-endosulfine (ARPP-19e)	1.41	0.013
Q9BUR5	*APOO*	MICOS complex subunit MIC26 (Apolipoprotein O) (MICOS complex subunit MIC23) (Protein FAM121B)	1.68	0.014
K7ELL7	*PRKCSH*	Glucosidase 2 subunit beta (Glucosidase II subunit beta)	1.39	0.014
Q14254	*FLOT2*	Flotillin-2 (Epidermal surface antigen) (ESA) (Membrane component chromosome 17 surface marker 1)	1.33	0.014
Q14766	*LTBP1*	Latent-transforming growth factor beta-binding protein 1 (LTBP-1) (Transforming growth factor beta-1-binding protein 1) (TGF-beta1-BP-1)	1.50	0.014
Q6PUJ7	*HEL-215*	Prohibitin	1.33	0.014
Q9UBX3	*SLC25A10*	Mitochondrial dicarboxylate carrier (Solute carrier family 25 member 10)	1.82	0.015
P16671	*CD36*	Platelet glycoprotein 4 (Fatty acid translocase) (FAT) (Glycoprotein IIIb) (GPIIIB) (Leukocyte differentiation antigen CD36) (PAS IV) (PAS-4) (Platelet collagen receptor) (Platelet glycoprotein IV) (GPIV) (Thrombospondin receptor) (CD antigen CD36)	1.46	0.015
A0A096WXL7	*ATP8*	ATP synthase protein 8	1.65	0.016
P09874	*PARP1*	Poly [ADP-ribose] polymerase 1 (PARP-1) (EC 2.4.2.30) (ADP-ribosyltransferase diphtheria toxin-like 1) (ARTD1) (DNA ADP-ribosyltransferase PARP1) (EC 2.4.2.-) (NAD(+) ADP-ribosyltransferase 1) (ADPRT 1) (Poly[ADP-ribose] synthase 1) (Protein poly-ADP-ribosyltransferase PARP1) (EC 2.4.2.-)	1.34	0.016
Q8NBX0	*SCCPDH*	Saccharopine dehydrogenase-like oxidoreductase (EC 1.-.-.-)	1.46	0.017
O43896	*KIF1C*	Kinesin-like protein KIF1C	0.64	0.017
O95881	*TXNDC12*	Thioredoxin domain-containing protein 12 (EC 1.8.4.2) (Endoplasmic reticulum resident protein 18) (ER protein 18) (ERp18) (Endoplasmic reticulum resident protein 19) (ER protein 19) (ERp19) (Thioredoxin-like protein p19) (hTLP19)	1.44	0.017
O43795	*MYO1B*	Unconventional myosin-Ib (MYH-1c) (Myosin I alpha) (MMI-alpha) (MMIa)	0.55	0.018
Q9NSE4	*IARS2*	Isoleucine--tRNA ligase, mitochondrial (EC 6.1.1.5) (Isoleucyl-tRNA synthetase) (IleRS)	1.35	0.018

O00159	*MYO1C*	Unconventional myosin-Ic (Myosin I beta) (MMI-beta) (MMIb)	0.69	0.018
O95299	*NDUFA10*	NADH dehydrogenase [ubiquinone] 1 alpha subcomplex subunit 10, mitochondrial (Complex I-42kD) (CI-42kD) (NADH-ubiquinone oxidoreductase 42 kDa subunit)	1.54	0.019
P30405	*PPIF*	Peptidyl-prolyl *cis-trans* isomerase F, mitochondrial (PPIase F) (EC 5.2.1.8) (Cyclophilin D) (CyP-D) (CypD) (Cyclophilin F) (Mitochondrial cyclophilin) (CyP-M) (Rotamase F)	1.31	0.019
Q93062	*RBPMS*	RNA-binding protein with multiple splicing (RBP-MS) (Heart and RRM expressed sequence) (Hermes)	1.65	0.021
Q8NBU5	*ATAD1*	Outer mitochondrial transmembrane helix translocase (EC 7.4.2.-) (ATPase family AAA domain-containing protein 1) (hATAD1) (Thorase)	1.41	0.021
Q01449	*MYL7*	Myosin regulatory light chain 2, atrial isoform (MLC-2a) (MLC2a) (Myosin light chain 2a) (Myosin regulatory light chain 7)	1.52	0.022
Q96PE7	*MCEE*	Methylmalonyl-CoA epimerase, mitochondrial (EC 5.1.99.1) (DL-methylmalonyl-CoA racemase)	1.39	0.022
O96000	*NDUFB10*	NADH dehydrogenase [ubiquinone] 1 beta subcomplex subunit 10 (Complex I-PDSW) (CI-PDSW) (NADH-ubiquinone oxidoreductase PDSW subunit)	1.44	0.023
Q96EY1	*DNAJA3*	DnaJ homolog subfamily A member 3, mitochondrial (DnaJ protein Tid-1) (hTid-1) (Hepatocellular carcinoma-associated antigen 57) (Tumorous imaginal discs protein Tid56 homolog)	1.52	0.023
Q13011	*ECH1*	Delta(3,5)-Delta(2,4)-dienoyl-CoA isomerase, mitochondrial (EC 5.3.3.-)	1.38	0.023
P50453	*SERPINB9*	Serpin B9 (Cytoplasmic antiproteinase 3) (CAP-3) (CAP3) (Peptidase inhibitor 9) (PI-9)	1.59	0.024
P19404	*NDUFV2*	NADH dehydrogenase [ubiquinone] flavoprotein 2, mitochondrial (EC 7.1.1.2) (NADH-ubiquinone oxidoreductase 24 kDa subunit)	1.46	0.024
P19367	*HK1*	Hexokinase-1 (EC 2.7.1.1) (Brain form hexokinase) (Hexokinase type I) (HK I) (Hexokinase-A)	1.47	0.024
Q13061	*TRDN*	Triadin	2.56	0.024
P49821	*NDUFV1*	NADH dehydrogenase [ubiquinone] flavoprotein 1, mitochondrial (EC 7.1.1.2) (Complex I-51kD) (CI-51kD) (NADH dehydrogenase flavoprotein 1) (NADH-ubiquinone oxidoreductase 51 kDa subunit)	1.52	0.025

D3DRP5	*C9orf19*	Chromosome 9 open reading frame 19, isoform CRA_a (Fragment)	1.51	0.025
O95155	*UBE4B*	Ubiquitin conjugation factor E4 B (EC 2.3.2.27) (Homozygously deleted in neuroblastoma 1) (RING-type E3 ubiquitin transferase E4 B) (Ubiquitin fusion degradation protein 2)	0.74	0.025
P05546	*SERPIND1*	Heparin cofactor 2 (Heparin cofactor II) (HC-II) (Protease inhibitor leuserpin-2) (HLS2) (Serpin D1)	0.73	0.026
P13533	*MYH6*	Myosin-6 (Myosin heavy chain 6) (Myosin heavy chain, cardiac muscle alpha isoform) (MyHC-alpha)	0.51	0.026
Q07065	*CKAP4*	Cytoskeleton-associated protein 4 (63-kDa cytoskeleton-linking membrane protein) (Climp-63) (p63)	1.30	0.026
P55196	*AFDN*	Afadin (ALL1-fused gene from chromosome 6 protein) (Protein AF-6) (Afadin adherens junction formation factor)	1.36	0.026
P02794	*FTH1*	Ferritin heavy chain (Ferritin H subunit) (EC 1.16.3.1) (Cell proliferation-inducing gene 15 protein) [Cleaved into: Ferritin heavy chain, N-terminally processed]	0.65	0.027
P45984	*MAPK9*	Mitogen-activated protein kinase 9 (MAP kinase 9) (MAPK 9) (EC 2.7.11.24) (JNK-55) (Stress-activated protein kinase 1a) (SAPK1a) (Stress-activated protein kinase JNK2) (c-Jun N-terminal kinase 2)	1.32	0.027
Q6UWP7	*LCLAT1*	Lysocardiolipin acyltransferase 1 (EC 2.3.1.-) (1-acylglycerol-3-phosphate O-acyltransferase 8) (1-AGP acyltransferase 8) (1-AGPAT 8) (EC 2.3.1.51) (Acyl-CoA:lysocardiolipin acyltransferase 1)	1.76	0.028
O75438	*NDUFB1*	NADH dehydrogenase [ubiquinone] 1 beta subcomplex subunit 1 (Complex I-MNLL) (CI-MNLL) (NADH-ubiquinone oxidoreductase MNLL subunit)	1.43	0.029
A0A5C2GW15		IG c1457_light_IGKV3-20_IGKJ1 (Fragment)	0.73	0.029
A0A024R9Q1	*THBS1*	Thrombospondin 1, isoform CRA_a	2.29	0.030
B7Z6P1		cDNA FLJ53662, highly similar to Actin, alpha skeletal muscle	0.63	0.030
Q9NUP9	*LIN7C*	Protein lin-7 homolog C (Lin-7C) (Mammalian lin-seven protein 3) (MALS-3) (Vertebrate lin-7 homolog 3) (Veli-3)	2.09	0.032

Q16718	*NDUFA5*	NADH dehydrogenase [ubiquinone] 1 alpha subcomplex subunit 5 (Complex I subunit B13) (Complex I-13kD-B) (CI-13kD-B) (NADH-ubiquinone oxidoreductase 13 kDa-B subunit)	1.62	0.033
Q8N6H7	*ARFGAP2*	ADP-ribosylation factor GTPase-activating protein 2 (ARF GAP 2) (GTPase-activating protein ZNF289) (Zinc finger protein 289)	0.68	0.033
Q9BWU5	*HBB*	Mutant hemoglobin beta chain (Fragment)	5.44	0.033
P09417	*QDPR*	Dihydropteridine reductase (EC 1.5.1.34) (HDHPR) (Quinoid dihydropteridine reductase) (Short chain dehydrogenase/reductase family 33C member 1)	1.55	0.033
Q9Y4F5	*CEP170B*	Centrosomal protein of 170 kDa protein B (Centrosomal protein 170B) (Cep170B)	0.28	0.033
D3DPF9	*TTN*	Titin, isoform CRA_b	1.32	0.033
P06132	*UROD*	Uroporphyrinogen decarboxylase (UPD) (URO-D) (EC 4.1.1.37)	1.38	0.033
B4DR48		Arginyl-tRNA--protein transferase 1 (Arginyltransferase 1) (R-transferase 1) (EC 2.3.2.8) (Arginine-tRNA--protein transferase 1)	3.00	0.034
Q8TCC3	*MRPL30*	39S ribosomal protein L30, mitochondrial (L30mt) (MRP-L30) (39S ribosomal protein L28, mitochondrial) (L28mt) (MRP-L28) (Mitochondrial large ribosomal subunit protein uL30m)	1.38	0.034
P16219	*ACADS*	Short-chain specific acyl-CoA dehydrogenase, mitochondrial (SCAD) (EC 1.3.8.1) (Butyryl-CoA dehydrogenase)	1.82	0.035
P02746	*C1QB*	Complement C1q subcomponent subunit B	0.59	0.035
Q96S95	*CAMK2N2*	Calcium/calmodulin-dependent protein kinase II inhibitor 2 (CaM-KII inhibitory protein) (CaM-KIIN)	0.22	0.035
O95178	*NDUFB2*	NADH dehydrogenase [ubiquinone] 1 beta subcomplex subunit 2, mitochondrial (Complex I-AGGG) (CI-AGGG) (NADH-ubiquinone oxidoreductase AGGG subunit)	1.49	0.035
Q9NRX2	*MRPL17*	39S ribosomal protein L17, mitochondrial (L17mt) (MRP-L17) (LYST-interacting protein 2) (Mitochondrial large ribosomal subunit protein bL17m)	0.74	0.036
Q9HB00	*DSC1*	Desmocollin 1, isoform CRA_b (Desmocollin 1b)	1.51	0.036

E5KND7	*GFM1*	Elongation factor G, mitochondrial (EF-Gmt) (Elongation factor G 1, mitochondrial) (mEF-G 1) (Elongation factor G1)	1.48	0.036
Q5U5X0	*LYRM7*	Complex III assembly factor LYRM7 (LYR motif-containing protein 7)	1.38	0.037
Q9BV79	*MECR*	Enoyl-[acyl-carrier-protein] reductase, mitochondrial (EC 1.3.1.104) (2-enoyl thioester reductase) (Nuclear receptor-binding factor 1) (HsNrbf-1) (NRBF-1)	1.33	0.037
Q9Y4E8	*USP15*	Ubiquitin carboxyl-terminal hydrolase 15 (EC 3.4.19.12) (Deubiquitinating enzyme 15) (Ubiquitin thioesterase 15) (Ubiquitin-specific-processing protease 15) (Unph-2) (Unph4)	1.34	0.038
P13804	*ETFA*	Electron transfer flavoprotein subunit alpha, mitochondrial (Alpha-ETF)	1.34	0.038
Q9NWU1	*OXSM*	3-Oxoacyl-[acyl-carrier-protein] synthase, mitochondrial (EC 2.3.1.41) (Beta-ketoacyl-ACP synthase)	1.44	0.038
O95870	*ABHD16A*	Phosphatidylserine lipase ABHD16A (EC 3.1.-.-) (Alpha/beta hydrolase domain-containing protein 16A) (Abhydrolase domain-containing protein 16A) (HLA-B-associated transcript 5) (hBAT5) (Monoacylglycerol lipase ABHD16A) (EC 3.1.1.23) (Protein G5)	1.31	0.038
B9EEN6	*NADH5*	NADH-ubiquinone oxidoreductase chain 5 (EC 7.1.1.2)	1.49	0.038
Q96DP0		Complex I-9kD (NADH dehydrogenase [ubiquinone] flavoprotein 3, mitochondrial) (NADH-ubiquinone oxidoreductase 9 kDa subunit)	1.43	0.039
Q92599	*SEPTIN8*	Septin-8	0.73	0.039
P61619	*SEC61A1*	Protein transport protein Sec61 subunit alpha isoform 1 (Sec61 alpha-1)	1.66	0.039
P12829	*MYL4*	Myosin light chain 4 (Myosin light chain 1, embryonic muscle/atrial isoform) (Myosin light chain alkali GT-1 isoform)	0.51	0.040
Q9Y697	*NFS1*	Cysteine desulfurase, mitochondrial (EC 2.8.1.7)	1.34	0.040
O14548	*COX7A2L*	Cytochrome *c* oxidase subunit 7A-related protein, mitochondrial (COX7a-related protein) (Cytochrome *c* oxidase subunit VIIa-related protein) (EB1)	1.42	0.040

Q969Z3	*MTARC2*	Mitochondrial amidoxime reducing component 2 (mARC2) (EC 1.7.-.-) (Molybdenum cofactor sulfurase C-terminal domain-containing protein 2) (MOSC domain-containing protein 2) (Moco sulfurase C-terminal domain-containing protein 2)	1.35	0.040
Q9GZY4	*COA1*	Cytochrome *c* oxidase assembly factor 1 homolog (Mitochondrial translation regulation assembly intermediate of cytochrome c oxidase protein of 15 kDa)	1.53	0.042
P06213	*INSR*	Insulin receptor (IR) (EC 2.7.10.1) (CD antigen CD220) [Cleaved into: Insulin receptor subunit alpha; Insulin receptor subunit beta]	1.45	0.042
Q7Z2W9	*MRPL21*	39S ribosomal protein L21, mitochondrial (L21mt) (MRP-L21) (Mitochondrial large ribosomal subunit protein bL21m)	0.54	0.042
P11233	*RALA*	Ras-related protein Ral-A (EC 3.6.5.2)	1.40	0.043
P08574	*CYC1*	Cytochrome c1, heme protein, mitochondrial (EC 7.1.1.8) (Complex III subunit 4) (Complex III subunit IV) (Cytochrome b-c1 complex subunit 4) (Ubiquinol-cytochrome-*c* reductase complex cytochrome c1 subunit) (Cytochrome *c*-1)	1.34	0.044
O00217	*NDUFS8*	NADH dehydrogenase [ubiquinone] iron-sulfur protein 8, mitochondrial (EC 7.1.1.2) (Complex I-23kD) (CI-23kD) (NADH-ubiquinone oxidoreductase 23 kDa subunit) (TYKY subunit)	1.35	0.046
Q96GK7	*FAHD2A*	Fumarylacetoacetate hydrolase domain-containing protein 2A (EC 3.-.-.-)	1.42	0.046
O14949	*UQCRQ*	Cytochrome b-c1 complex subunit 8 (Complex III subunit 8) (Complex III subunit VIII) (Ubiquinol-cytochrome c reductase complex 9.5 kDa protein) (Ubiquinol-cytochrome c reductase complex ubiquinone-binding protein QP-C)	1.36	0.046
P35270	*SPR*	Sepiapterin reductase (SPR) (EC 1.1.1.153)	1.61	0.046
P00734	*F2*	Prothrombin (EC 3.4.21.5) (Coagulation factor II) [Cleaved into: Activation peptide fragment 1; Activation peptide fragment 2; Thrombin light chain; Thrombin heavy chain]	1.67	0.046
P26885	*FKBP2*	Peptidyl-prolyl cis-trans isomerase FKBP2 (PPIase FKBP2) (EC 5.2.1.8) (13 kDa FK506-binding protein) (13 kDa FKBP) (FKBP-13) (FK506-binding protein 2) (FKBP-2) (Immunophilin FKBP13) (Rotamase)	1.32	0.047

P51991	*HNRNPA3*	Heterogeneous nuclear ribonucleoprotein A3 (hnRNP A3)	1.38	0.048
O14832	*PHYH*	Phytanoyl-CoA dioxygenase, peroxisomal (EC 1.14.11.18) (Phytanic acid oxidase) (Phytanoyl-CoA alpha-hydroxylase) (PhyH)	1.68	0.048
O95182	*NDUFA7*	NADH dehydrogenase [ubiquinone] 1 alpha subcomplex subunit 7 (Complex I-B14.5a) (CI-B14.5a) (NADH-ubiquinone oxidoreductase subunit B14.5a)	1.40	0.048
Q9UIQ6	*LNPEP*	Leucyl-cystinyl aminopeptidase (Cystinyl aminopeptidase) (EC 3.4.11.3) (Insulin-regulated membrane aminopeptidase) (Insulin-responsive aminopeptidase) (IRAP) (Oxytocinase) (OTase) (Placental leucine aminopeptidase) (P-LAP) [Cleaved into: Leucyl-cystinyl aminopeptidase, pregnancy serum form]	1.40	0.049
P19237	*TNNI1*	Troponin I, slow skeletal muscle (Troponin I, slow-twitch isoform)	3.70	0.049
O75947	*ATP5PD*	ATP synthase subunit d, mitochondrial (ATPase subunit d) (ATP synthase peripheral stalk subunit d)	1.34	0.050
Q8TAE6	*PPP1R14C*	Protein phosphatase 1 regulatory subunit 14C (Kinase-enhanced PP1 inhibitor) (PKC-potentiated PP1 inhibitory protein) (Serologically defined breast cancer antigen NY-BR-81)	1.52	0.050
P04156	*PRNP*	Major prion protein (PrP) (ASCR) (PrP27-30) (PrP33-35C) (CD antigen CD230)	3.42	0.050
P05388	*RPLP0*	60S acidic ribosomal protein P0 (60S ribosomal protein L10E) (Large ribosomal subunit protein uL10)	1.30	0.050
Proteins differentially (fold change >1.3 or <0.769) and significantly (P<0.05) expressed in post vs. pre samples in the RV of CAD patients. RV, right ventricle; CAD, coronary artery disease.				

Table SIV. Differentially expressed phosphoproteins from RV of CAD patients.					
Accession no.	Gene name	Description	Phosphosite	Fold change	P-value
P04792	*HSPB1*	Heat shock protein beta-1 (HspB1) (28 kDa heat shock protein) (Estrogen-regulated 24 kDa protein) (Heat shock 27 kDa protein) (HSP 27) (Stress-responsive protein 27) (SRP27)	S3(Phospho)	3.16	9.88E-05
E9PAV3	*NACA*	Nascent polypeptide-associated complex subunit alpha, muscle-specific form (Alpha-NAC, muscle-specific form) (skNAC)	S12(Phospho)	2.12	2.60E-04
E9PAV3	*NACA*	Nascent polypeptide-associated complex subunit alpha, muscle-specific form (Alpha-NAC, muscle-specific form) (skNAC)	S13(Phospho)	2.12	2.54E-03
Q6PKG0	*LARP1*	La-related protein 1 (La ribonucleoprotein domain family member 1)	S9(Phospho)	1.36	4.29E-03
Q2M3C7	*SPHKAP*	A-kinase anchor protein SPHKAP (SPHK1-interactor and AKAP domain-containing protein) (Sphingosine kinase type 1-interacting protein)	S3(Phospho)	0.54	1.24E-02
B4DUQ1		Heterogeneous nuclear ribonucleoprotein K	S6(Phospho)	0.70	1.55E-02
Q16539	*MAPK14*	Mitogen-activated protein kinase 14 (MAP kinase 14) (MAPK 14) (EC 2.7.11.24) (Cytokine suppressive anti-inflammatory drug-binding protein) (CSAID-binding protein) (CSBP) (MAP kinase MXI2) (MAX-interacting protein 2) (Mitogen-activated protein kinase p38 alpha) (MAP kinase p38 alpha) (Stress-activated protein kinase 2a) (SAPK2a)	T7(Phospho) Y9(Phospho)	1.92	2.18E-02
Q5VWP3	*MLIP*	Muscular LMNA-interacting protein (Cardiac Isl1-interacting protein) (CIP) (Muscular-enriched A-type laminin-interacting protein)	S9(Phospho)	0.47	3.55E-02
P40123	*CAP2*	Adenylyl cyclase-associated protein 2 (CAP 2)	S8(Phospho)	1.39	3.58E-02

Q13424	*SNTA1*	Alpha-1-syntrophin (59 kDa dystrophin-associated protein A1 acidic component 1) (Pro-TGF-alpha cytoplasmic domain-interacting protein 1) (TACIP1) (Syntrophin-1)	S4(Phospho)	0.42	3.70E-02
P23588	*EIF4B*	Eukaryotic translation initiation factor 4B (eIF-4B)	S10(Phospho) T6(Phospho)	1.54	3.84E-02
E9PAV3	*NACA*	Nascent polypeptide-associated complex subunit alpha, muscle-specific form (Alpha-NAC, muscle-specific form) (skNAC)	S4(Phospho)	1.94	4.43E-02
Q13424	*SNTA1*	Alpha-1-syntrophin (59 kDa dystrophin-associated protein A1 acidic component 1) (Pro-TGF-alpha cytoplasmic domain-interacting protein 1) (TACIP1) (Syntrophin-1)	S3(Phospho) S4(Phospho)	1.47	4.57E-02

Phosphoproteins whose expression relative to total protein expression is differentially (fold change >1.3 or <0.769) and significantly ($P<0.05$) expressed in post vs pre samples from the RV of CAD patients. RV, right ventricle; CAD, coronary artery disease.

Table SV. Differentially expressed proteins from LV and RV of AVS patients.					
	Accession no.	Gene name	Description	Fold change	P-value
LV	Q6Y1H2	*HACD2*	Very-long-chain (3R)-3-hydroxyacyl-CoA dehydratase 2 (EC 4.2.1.134) (3-hydroxyacyl-CoA dehydratase 2) (HACD2) (Protein-tyrosine phosphatase-like member B)	0.74	0.023
	Q9UBF2	*COPG2*	Coatomer subunit gamma-2 (Gamma-2-coat protein) (Gamma-2-COP)	0.69	0.035
	P04424	*ASL*	Argininosuccinate lyase (ASAL) (EC 4.3.2.1) (Arginosuccinase)	0.72	0.038
RV	P06132	*UROD*	Uroporphyrinogen decarboxylase (UPD) (URO-D) (EC 4.1.1.37)	1.42	0.037
	P98095	*FBLN2*	Fibulin-2 (FIBL-2)	0.66	0.044

Proteins differentially (fold change >1.3 or <0.769) and significantly (P<0.05) expressed in post vs pre samples in the LV and RV of AVS patients. LV, left ventricle; RV, right ventricle; AVS, aortic valve stenosis.

Table SVI. Enriched canonical pathways for the total protein analysis of the LV and RV of AVS patients.			
	Ingenuity canonical pathway	P-value of overlap	Molecules
LV	Citrulline-Nitric Oxide Cycle	0.005	ASL
	Arginine Biosynthesis IV	0.006	ASL
	Urea Cycle	0.006	ASL
	Superpathway of Citrulline Metabolism	0.014	ASL
	Coronavirus Replication Pathway	0.042	COPG2
RV	Reelin Signalling in Neurons	0.006	PAFAH1B1, RAC1
	Heme Biosynthesis from Uroporphyrinogen-III I	0.006	UROD
	Heme Biosynthesis II	0.013	UROD
	Role of p14/p19ARF in Tumor Suppression	0.017	RAC1
	Synaptogenesis Signalling Pathway	0.021	PAFAH1B1, RAC1
	PCP (Planar Cell Polarity) Pathway	0.023	RAC1
	Ephrin A Signalling	0.034	RAC1
	CSDE1 Signalling Pathway	0.046	RAC1
All significant pathways are shown for both ventricles. LV, left ventricle; RV, right ventricle; AVS, aortic valve stenosis.			

Table SVII. Differentially expressed phosphoproteins from LV and RV of AVS patients.						
	Accession no.	Gene name	Description	Phosphosite	Fold change	P-value
LV	P04792	*HSPB1*	Heat shock protein beta-1 (HspB1) (28 kDa heat shock protein) (Estrogen-regulated 24 kDa protein) (Heat shock 27 kDa protein) (HSP 27) (Stress-responsive protein 27) (SRP27)	S3(Phospho)	2.58	2.83E-06
	Q09666	*AHNAK*	Neuroblast differentiation-associated protein AHNAK (Desmoyokin)	S3(Phospho)	2.21	1.43E-05
	Q09666	*AHNAK*	Neuroblast differentiation-associated protein AHNAK (Desmoyokin)	S3(Phospho)	1.82	2.08E-03
	B7ZA42		cDNA, FLJ79056 (Fragment)	S11(Phospho)	1.45	7.67E-03
	Q7Z3B7	*DKFZp451N061*	Uncharacterized protein DKFZp451N061 (Fragment)	T6(Phospho)	1.77	8.70E-03
	P37802	*TAGLN2*	Transgelin-2 (Epididymis tissue protein Li 7e) (SM22-alpha homolog)	S3(Phospho)	1.89	1.48E-02
	Q09666	*AHNAK*	Neuroblast differentiation-associated protein AHNAK (Desmoyokin)	S9(Phospho)	0.74	1.50E-02
	Q9H987	*SYNPO2L*	Synaptopodin 2-like protein	S3(Phospho)	1.74	1.68E-02
	Q8WX93	*PALLD*	Palladin (SIH002) (Sarcoma antigen NY-SAR-77)	S3(Phospho)	2.83	2.00E-02
	Q13424	*SNTA1*	Alpha-1-syntrophin (59 kDa dystrophin-associated protein A1 acidic component 1) (Pro-TGF-alpha cytoplasmic domain-interacting protein 1) (TACIP1) (Syntrophin-1)	S3(Phospho)<br>S4(Phospho)	1.36	2.57E-02
	Q9UKG1	*APPL1*	DCC-interacting protein 13-alpha (Dip13-alpha) (Adapter protein containing PH domain, PTB domain and leucine zipper motif 1)	S14(Phospho)	0.58	3.07E-02
	A0A024RD15	*MAPK14*	Mitogen-activated protein kinase 14 (MAP kinase 14) (MAPK 14) (EC 2.7.11.24) (Cytokine suppressive anti-inflammatory drug-binding protein) (CSAID-binding protein) (CSBP) (MAP kinase MXI2) (MAX-interacting protein 2) (Mitogen-activated protein kinase p38 alpha) (MAP kinase p38 alpha) (Stress-activated protein kinase 2a) (SAPK2a)	T7(Phospho)<br>Y9(Phospho)	2.28	3.07E-02
	Q14157	*UBAP2L*	Ubiquitin-associated protein 2-like (Protein NICE-4)	S18(Phospho)	0.71	3.92E-02

	Q5SSJ5	*HP1BP3*	Heterochromatin protein 1-binding protein 3 (Protein HP1-BP74)	S16(Phospho)<br>S17(Phospho)<br>S21(Phospho)	1.87	4.73E-02
RV	P04792	*HSPB1*	Heat shock protein beta-1 (HspB1) (28 kDa heat shock protein) (Estrogen-regulated 24 kDa protein) (Heat shock 27 kDa protein) (HSP 27) (Stress-responsive protein 27) (SRP27)	S3(Phospho)	1.61	0.002
	Q8NE79	*BVES*	Blood vessel epicardial substance (hBVES) (Popeye domain-containing protein 1) (Popeye protein 1)	S19(Phospho)	1.97	0.017
	Q09666	*AHNAK*	Neuroblast differentiation-associated protein AHNAK (Desmoyokin)	S3(Phospho)	1.71	0.031
	P02511	*CRYAB*	Alpha-crystallin B chain (Alpha(B)-crystallin) (Heat shock protein beta-5) (HspB5) (Renal carcinoma antigen NY-REN-27) (Rosenthal fiber component)	Ambiguous	1.92	0.049

Phosphoproteins whose expression relative to total protein expression is differentially (fold change >1.3 or <0.769) and significantly (P<0.05) expressed in post vs pre samples from the LV and RV of AVS patients. LV, left ventricle; RV, right ventricle; AVS, aortic valve stenosis.

Table SVIII. Differentially expressed proteins from LV pre-ischaemic cardioplegic arrest samples between AVS and CAD patients.				
Accession no.	Gene name	Description	Fold change	P-value
P62993	*GRB2*	Growth factor receptor-bound protein 2 (Adapter protein GRB2) (Protein Ash) (SH2/SH3 adapter GRB2)	0.43	0.000
Q99439	*CNN2*	Calponin-2 (Calponin H2, smooth muscle) (Neutral calponin)	0.73	0.000
O43815	*STRN*	Striatin	0.62	0.000
A0A384NYH5	*ANKRD1*	Ankyrin repeat domain 1 (Cardiac muscle) (Epididymis secretory sperm binding protein)	0.60	0.000
A0A0A6YYL6	*RPL17-C18orf32*	60S ribosomal protein L17	0.67	0.000
Q99747	*NAPG*	Gamma-soluble NSF attachment protein (SNAP-gamma) (N-ethylmaleimide-sensitive factor attachment protein gamma)	0.73	0.001
Q9Y2D4	*EXOC6B*	Exocyst complex component 6B (Exocyst complex component Sec15B) (SEC15-like protein 2)	0.64	0.002
Q9NTZ6	*RBM12*	RNA-binding protein 12 (RNA-binding motif protein 12) (SH3/WW domain anchor protein in the nucleus) (SWAN)	0.47	0.002
Q96A35	*MRPL24*	39S ribosomal protein L24, mitochondrial (L24mt) (MRP-L24) (Mitochondrial large ribosomal subunit protein uL24m)	2.04	0.002
A0A0S2Z530	*LDB3*	LIM domain binding 3 isoform 1 (LIM domain binding 3, isoform CRA_h) (Fragment)	0.68	0.002
Q92688	*ANP32B*	Acidic leucine-rich nuclear phosphoprotein 32 family member B (Acidic protein rich in leucines) (Putative HLA-DR-associated protein I-2) (PHAPI2) (Silver-stainable protein SSP29)	0.44	0.002
Q8IYQ7	*THNSL1*	Threonine synthase-like 1 (TSH1)	1.46	0.003
Q86YM7	*HOMER1*	Homer protein homolog 1 (Homer-1)	0.65	0.003
O60506	*SYNCRIP*	Heterogeneous nuclear ribonucleoprotein Q (hnRNP Q) (Glycine- and tyrosine-rich RNA-binding protein) (GRY-RBP) (NS1-associated protein 1) (Synaptotagmin-binding, cytoplasmic RNA-interacting protein)	0.70	0.003

O95881	*TXNDC12*	Thioredoxin domain-containing protein 12 (EC 1.8.4.2) (Endoplasmic reticulum resident protein 18) (ER protein 18) (ERp18) (Endoplasmic reticulum resident protein 19) (ER protein 19) (ERp19) (Thioredoxin-like protein p19) (hTLP19)	0.74	0.003
A0A3S6RG84	*HLA-C*	MHC class I antigen	0.51	0.004
P42677	*RPS27*	40S ribosomal protein S27 (Metallopan-stimulin 1) (MPS-1) (Small ribosomal subunit protein eS27)	0.72	0.004
P13591	*NCAM1*	Neural cell adhesion molecule 1 (N-CAM-1) (NCAM-1) (CD antigen CD56)	0.70	0.004
P24043	*LAMA2*	Laminin subunit alpha-2 (Laminin M chain) (Laminin-12 subunit alpha) (Laminin-2 subunit alpha) (Laminin-4 subunit alpha) (Merosin heavy chain)	0.69	0.004
P08648	*ITGA5*	Integrin alpha-5 (CD49 antigen-like family member E) (Fibronectin receptor subunit alpha) (Integrin alpha-F) (VLA-5) (CD antigen CD49e) [Cleaved into: Integrin alpha-5 heavy chain; Integrin alpha-5 light chain]	0.64	0.004
Q96PE2	*ARHGEF17*	Rho guanine nucleotide exchange factor 17 (164 kDa Rho-specific guanine-nucleotide exchange factor) (p164-RhoGEF) (p164RhoGEF) (Tumor endothelial marker 4)	0.67	0.004
A8K3M3	*PTPN1*	Tyrosine-protein phosphatase non-receptor type (EC 3.1.3.48)	0.73	0.004
Q96K76	*USP47*	Ubiquitin carboxyl-terminal hydrolase 47 (EC 3.4.19.12) (Deubiquitinating enzyme 47) (Ubiquitin thioesterase 47) (Ubiquitin-specific-processing protease 47)	1.82	0.004
P54289	*CACNA2D1*	Voltage-dependent calcium channel subunit alpha-2/delta-1 (Voltage-gated calcium channel subunit alpha-2/delta-1) [Cleaved into: Voltage-dependent calcium channel subunit alpha-2-1; Voltage-dependent calcium channel subunit delta-1]	0.74	0.004
A0A0S2Z4Q2	*TGFBI*	Transforming growth factor-beta-induced protein ig-h3 (Fragment)	0.54	0.005
P17050	*NAGA*	Alpha-N-acetylgalactosaminidase (EC 3.2.1.49) (Alpha-galactosidase B)	1.55	0.005
Q02750	*MAP2K1*	Dual specificity mitogen-activated protein kinase kinase 1 (MAP kinase kinase 1) (MAPKK 1) (MKK1) (EC 2.7.12.2) (ERK activator kinase 1) (MAPK/ERK kinase 1) (MEK 1)	0.58	0.005
Q5JPE7	*NOMO2*	Nodal modulator 2 (pM5 protein 2)	0.60	0.006

P62834	*RAP1A*	Ras-related protein Rap-1A (EC 3.6.5.2) (C21KG) (G-22K) (GTP-binding protein smg p21A) (Ras-related protein Krev-1)	0.72	0.006
Q9BZL4	*PPP1R12C*	Protein phosphatase 1 regulatory subunit 12C (Protein phosphatase 1 myosin-binding subunit of 85 kDa) (Protein phosphatase 1 myosin-binding subunit p85)	0.58	0.006
A0A024R324	*RHOA*	Epididymis secretory sperm binding protein (Ras homolog gene family, member A, isoform CRA_a)	0.71	0.006
Q9NR31	*SAR1A*	GTP-binding protein SAR1a (COPII-associated small GTPase)	0.76	0.007
P30626	*SRI*	Sorcin (22 kDa protein) (CP-22) (CP22) (V19)	0.62	0.007
Q96BN8	*OTULIN*	Ubiquitin thioesterase otulin (EC 3.4.19.12) (Deubiquitinating enzyme otulin) (OTU domain-containing deubiquitinase with linear linkage specificity) (Ubiquitin thioesterase Gumby)	0.70	0.007
O95671	*ASMTL*	Probable bifunctional dTTP/UTP pyrophosphatase/methyltransferase protein [Includes: dTTP/UTP pyrophosphatase (dTTPase/UTPase) (EC 3.6.1.9) (Nucleoside triphosphate pyrophosphatase) (Nucleotide pyrophosphatase) (Nucleotide PPase); N-acetylserotonin O-methyltransferase-like protein (ASMTL) (EC 2.1.1.-)]	0.48	0.007
P29966	*MARCKS*	Myristoylated alanine-rich C-kinase substrate (MARCKS) (Protein kinase C substrate, 80 kDa protein, light chain) (80K-L protein) (PKCSL)	0.58	0.007
P26368	*U2AF2*	Splicing factor U2AF 65 kDa subunit (U2 auxiliary factor 65 kDa subunit) (hU2AF(65)) (hU2AF65) (U2 snRNP auxiliary factor large subunit)	0.55	0.007
A0A384NPH9		Glypican-1 (Secreted glypican-1)	0.72	0.008
Q96I59	*NARS2*	Probable asparagine--tRNA ligase, mitochondrial (EC 6.1.1.22) (Asparaginyl-tRNA synthetase) (AsnRS)	1.41	0.008
Q15056	*EIF4H*	Eukaryotic translation initiation factor 4H (eIF-4H) (Williams-Beuren syndrome chromosomal region 1 protein)	0.65	0.008
Q5TC12	*ATPAF1*	ATP synthase mitochondrial F1 complex assembly factor 1 (ATP11 homolog)	1.56	0.008
Q99541	*PLIN2*	Perilipin-2 (Adipophilin) (Adipose differentiation-related protein) (ADRP)	2.12	0.008

Q9NVT9	*ARMC1*	Armadillo repeat-containing protein 1	0.65	0.008
A0A024R977	*ARL8A*	ADP-ribosylation factor-like 8A, isoform CRA_a	0.41	0.008
Q8NDI1	*EHBP1*	EH domain-binding protein 1	0.74	0.008
A1L172	*ACOT1*	Acyl-CoA thioesterase 1	1.51	0.008
O95487	*SEC24B*	Protein transport protein Sec24B (SEC24-related protein B)	0.49	0.008
P07737	*PFN1*	Profilin-1 (Epididymis tissue protein Li 184a) (Profilin I)	0.72	0.008
Q5T160	*RARS2*	Probable arginine--tRNA ligase, mitochondrial (EC 6.1.1.19) (Arginyl-tRNA synthetase) (ArgRS)	1.42	0.008
Q6IPR1	*ETFRF1*	Electron transfer flavoprotein regulatory factor 1 (LYR motif-containing protein 5)	1.70	0.008
P46379	*BAG6*	Large proline-rich protein BAG6 (BAG family molecular chaperone regulator 6) (BCL2-associated athanogene 6) (BAG-6) (HLA-B-associated transcript 3) (Protein G3) (Protein Scythe)	1.71	0.009
P63172	*DYNLT1*	Dynein light chain Tctex-type 1 (Protein CW-1) (T-complex testis-specific protein 1 homolog)	0.74	0.009
P08910	*ABHD2*	Monoacylglycerol lipase ABHD2 (EC 3.1.1.23) (2-arachidonoylglycerol hydrolase) (Abhydrolase domain-containing protein 2) (Acetylesterase) (EC 3.1.1.6) (Lung alpha/beta hydrolase 2) (Progesterone-sensitive lipase) (EC 3.1.1.79) (Protein PHPS1-2)	0.60	0.009
F6VCX5	*PHKG1*	Phosphorylase kinase (EC 2.7.11.19)	1.40	0.009
Q9UBR2	*CTSZ*	Cathepsin Z (EC 3.4.18.1) (Cathepsin P) (Cathepsin X)	0.52	0.009
A0A0F7KYT8	*FXR1*	Fragile X mental retardation autosomal homolog variant p2K (Fragile X mental retardation, autosomal homolog 1, isoform CRA_g)	0.65	0.010
A0A024R648	*TIMM9*	Translocase of inner mitochondrial membrane 9 homolog (Yeast), isoform CRA_a	0.60	0.010
Q96S66	*CLCC1*	Chloride channel CLIC-like protein 1 (Mid-1-related chloride channel protein 1)	0.67	0.010
Q6IB54	*ATP5J*	ATP synthase-coupling factor 6, mitochondrial (ATPase subunit F6)	0.70	0.010
O15144	*ARPC2*	Actin-related protein 2/3 complex subunit 2 (Arp2/3 complex 34 kDa subunit) (p34-ARC)	0.61	0.010
Q96HE7	*ERO1A*	ERO1-like protein alpha (ERO1-L) (ERO1-L-alpha) (EC 1.8.4.-) (Endoplasmic oxidoreductin-1-like protein) (Endoplasmic reticulum oxidoreductase alpha) (Oxidoreductin-1-L-alpha)	0.48	0.010
A0A024R5K1	*CORO1B*	Coronin	1.64	0.010

Q9BXX0	*EMILIN2*	EMILIN-2 (Elastin microfibril interface-located protein 2) (Elastin microfibril interfacer 2) (Protein FOAP-10)	0.16	0.010
Q96N66	*MBOAT7*	Lysophospholipid acyltransferase 7 (LPLAT 7) (EC 2.3.1.-) (1-acylglycerophosphatidylinositol O-acyltransferase) (Bladder and breast carcinoma-overexpressed gene 1 protein) (Leukocyte receptor cluster member 4) (Lysophosphatidylinositol acyltransferase) (LPIAT) (Lyso-PI acyltransferase) (Membrane-bound O-acyltransferase domain-containing protein 7) (O-acyltransferase domain-containing protein 7) (h-mboa-7)	0.55	0.011
P62304	*SNRPE*	Small nuclear ribonucleoprotein E (snRNP-E) (Sm protein E) (Sm-E) (SmE)	0.66	0.011
Q9Y3U8	*RPL36*	60S ribosomal protein L36 (Large ribosomal subunit protein eL36)	0.46	0.012
Q567U6	*CCDC93*	Coiled-coil domain-containing protein 93	0.56	0.012
P29558	*RBMS1*	RNA-binding motif, single-stranded-interacting protein 1 (Single-stranded DNA-binding protein MSSP-1) (Suppressor of CDC2 with RNA-binding motif 2)	0.58	0.012
P07738	*BPGM*	Bisphosphoglycerate mutase (BPGM) (EC 5.4.2.4) (2,3-bisphosphoglycerate mutase, erythrocyte) (2,3-bisphosphoglycerate synthase) (EC 5.4.2.11) (2,3-diphosphoglycerate mutase) (DPGM) (BPG-dependent PGAM)	1.70	0.012
O60551	*NMT2*	Glycylpeptide N-tetradecanoyltransferase 2 (EC 2.3.1.97) (Myristoyl-CoA:protein N-myristoyltransferase 2) (NMT 2) (Peptide N-myristoyltransferase 2) (Type II N-myristoyltransferase)	1.69	0.013
Q12905	*ILF2*	Interleukin enhancer-binding factor 2 (Nuclear factor of activated T-cells 45 kDa)	0.72	0.013
A0A5C2G7C2		IGH c467_heavy__IGHV5-51_IGHD5-12_IGHJ4 (Fragment)	1.78	0.013
P56199	*ITGA1*	Integrin alpha-1 (CD49 antigen-like family member A) (Laminin and collagen receptor) (VLA-1) (CD antigen CD49a)	0.74	0.013
O43399	*TPD52L2*	Tumor protein D54 (hD54) (Tumor protein D52-like 2)	0.76	0.013
P05164	*MPO*	Myeloperoxidase (MPO) (EC 1.11.2.2) [Cleaved into: Myeloperoxidase; 89 kDa myeloperoxidase; 84 kDa myeloperoxidase; Myeloperoxidase light chain; Myeloperoxidase heavy chain]	0.61	0.013

P31946	*YWHAB*	14-3-3 protein beta/alpha (Protein 1054) (Protein kinase C inhibitor protein 1) (KCIP-1) [Cleaved into: 14-3-3 protein beta/alpha, N-terminally processed]	0.75	0.013
O95197	*RTN3*	Reticulon-3 (Homolog of ASY protein) (HAP) (Neuroendocrine-specific protein-like 2) (NSP-like protein 2) (Neuroendocrine-specific protein-like II) (NSP-like protein II) (NSPLII)	0.61	0.013
V9HW41	*HEL-S-71*	Epididymis secretory protein Li 71 (Ubiquitin-conjugating enzyme E2N (UBC13 homolog, yeast), isoform CRA_a)	0.75	0.014
Q9C0C2	*TNKS1BP1*	182 kDa tankyrase-1-binding protein	1.38	0.014
B7Z4K1		cDNA FLJ50104, highly similar to Alpha-actinin-2	0.60	0.014
Q9HAV0	*GNB4*	Guanine nucleotide-binding protein subunit beta-4 (Transducin beta chain 4)	0.54	0.014
H0YJ34	*FERMT2*	Fermitin family homolog 2 (Fragment)	1.55	0.014
Q9H008	*LHPP*	Phospholysine phosphohistidine inorganic pyrophosphate phosphatase (hLHPP) (EC 3.1.3.-) (EC 3.6.1.1)	0.39	0.014
P23368	*ME2*	NAD-dependent malic enzyme, mitochondrial (NAD-ME) (EC 1.1.1.38) (Malic enzyme 2)	0.59	0.014
Q9NQE9	*HINT3*	Histidine triad nucleotide-binding protein 3 (HINT-3) (EC 3.-.-.-)	0.60	0.015
A0A140VJK7		5'-deoxynucleotidase HDDC2 (EC 3.1.3.89) (HD domain-containing protein 2)	0.39	0.015
O75396	*SEC22B*	Vesicle-trafficking protein SEC22b (ER-Golgi SNARE of 24 kDa) (ERS-24) (ERS24) (SEC22 vesicle-trafficking protein homolog B) (SEC22 vesicle-trafficking protein-like 1)	0.65	0.015
Q14515	*SPARCL1*	SPARC-like protein 1 (High endothelial venule protein) (Hevin) (MAST 9)	0.57	0.015
O00764	*PDXK*	Pyridoxal kinase (EC 2.7.1.35) (Pyridoxine kinase)	1.42	0.016
P14866	*HNRNPL*	Heterogeneous nuclear ribonucleoprotein L (hnRNP L)	0.74	0.016
F6S8M0	*GNS*	N-acetylglucosamine-6-sulfatase	0.73	0.016
Q16363	*LAMA4*	Laminin subunit alpha-4 (Laminin-14 subunit alpha) (Laminin-8 subunit alpha) (Laminin-9 subunit alpha)	0.74	0.016
O60488	*ACSL4*	Long-chain-fatty-acid--CoA ligase 4 (EC 6.2.1.3) (Arachidonate--CoA ligase) (EC 6.2.1.15) (Long-chain acyl-CoA synthetase 4) (LACS 4)	0.64	0.016

A0A024R4M0	*RPS9*	40S ribosomal protein S9	0.75	0.016
Q9NVA2	*SEPTIN11*	Septin-11	0.62	0.016
P10301	*RRAS*	Ras-related protein R-Ras (EC 3.6.5.-) (p23)	0.77	0.016
P27361	*MAPK3*	Mitogen-activated protein kinase 3 (MAP kinase 3) (MAPK 3) (EC 2.7.11.24) (ERT2) (Extracellular signal-regulated kinase 1) (ERK-1) (Insulin-stimulated MAP2 kinase) (MAP kinase isoform p44) (p44-MAPK) (Microtubule-associated protein 2 kinase) (p44-ERK1)	0.71	0.016
Q9BQE3	*TUBA1C*	Tubulin alpha-1C chain (Alpha-tubulin 6) (Tubulin alpha-6 chain) [Cleaved into: Detyrosinated tubulin alpha-1C chain]	0.72	0.017
A0A024RBY9	*HCCS*	Cytochrome c heme lyase (EC 4.4.1.17)	1.47	0.017
Q9NY65	*TUBA8*	Tubulin alpha-8 chain (Alpha-tubulin 8) (Tubulin alpha chain-like 2)	0.73	0.017
A0A024R9G4	*FAM49B*	Family with sequence similarity 49, member B, isoform CRA_a	0.64	0.017
O15173	*PGRMC2*	Membrane-associated progesterone receptor component 2 (Progesterone membrane-binding protein) (Steroid receptor protein DG6)	0.65	0.017
P05556	*ITGB1*	Integrin beta-1 (Fibronectin receptor subunit beta) (Glycoprotein IIa) (GPIIA) (VLA-4 subunit beta) (CD antigen CD29)	0.76	0.017
Q15172	*PPP2R5A*	Serine/threonine-protein phosphatase 2A 56 kDa regulatory subunit alpha isoform (PP2A B subunit isoform B'-alpha) (PP2A B subunit isoform B56-alpha) (PP2A B subunit isoform PR61-alpha) (PR61alpha) (PP2A B subunit isoform R5-alpha)	0.72	0.018
Q8TAE8	*GADD45GIP1*	Growth arrest and DNA damage-inducible proteins-interacting protein 1 (39S ribosomal protein L59, mitochondrial) (MRP-L59) (CKII beta-associating protein) (CR6-interacting factor 1) (CRIF1) (Mitochondrial large ribosomal subunit protein mL64) (Papillomavirus L2-interacting nuclear protein 1) (PLINP) (PLINP-1) (p53-responsive gene 6 protein)	0.53	0.018
Q6MZY5	*DKFZp686N08224*	Uncharacterized protein DKFZp686N08224	0.74	0.018
Q9BRT2	*UQCC2*	Ubiquinol-cytochrome-c reductase complex assembly factor 2 (Breast cancer-associated protein SGA-81M) (Mitochondrial nucleoid factor 1) (Mitochondrial protein M19)	1.30	0.018

P98196	*ATP11A*	Probable phospholipid-transporting ATPase IH (EC 7.6.2.1) (ATPase IS) (ATPase class VI type 11A) (P4-ATPase flippase complex alpha subunit ATP11A)	0.71	0.018
P62873	*GNB1*	Guanine nucleotide-binding protein G(I)/G(S)/G(T) subunit beta-1 (Transducin beta chain 1)	0.58	0.019
Q4U2R6	*MRPL51*	39S ribosomal protein L51, mitochondrial (L51mt) (MRP-L51) (Mitochondrial large ribosomal subunit protein mL51) (bMRP-64) (bMRP64)	2.23	0.019
K7ERC8	*KDSR*	3-ketodihydrosphingosine reductase	0.34	0.019
D3DNI2	*PFN2*	Profilin (Fragment)	0.57	0.019
P02654	*APOC1*	Apolipoprotein C-I (Apo-CI) (ApoC-I) (Apolipoprotein C1) [Cleaved into: Truncated apolipoprotein C-I]	2.53	0.019
A0A140VK64		E3 ubiquitin-protein ligase subunit KPC2 (Kip1 ubiquitination-promoting complex protein 2) (Ubiquitin-associated domain-containing protein 1)	1.49	0.019
Q9P2B2	*PTGFRN*	Prostaglandin F2 receptor negative regulator (CD9 partner 1) (CD9P-1) (Glu-Trp-Ile EWI motif-containing protein F) (EWI-F) (Prostaglandin F2-alpha receptor regulatory protein) (Prostaglandin F2-alpha receptor-associated protein) (CD antigen CD315)	0.67	0.020
Q9Y394	*DHRS7*	Dehydrogenase/reductase SDR family member 7 (EC 1.1.-.-) (Retinal short-chain dehydrogenase/reductase 4) (retSDR4) (Short chain dehydrogenase/reductase family 34C member 1)	0.76	0.020
O95810	*CAVIN2*	Caveolae-associated protein 2 (Cavin-2) (PS-p68) (Phosphatidylserine-binding protein) (Serum deprivation-response protein)	0.73	0.020
Q9NQH7	*XPNPEP3*	Xaa-Pro aminopeptidase 3 (X-Pro aminopeptidase 3) (EC 3.4.11.9) (Aminopeptidase P3) (APP3)	1.39	0.020
P02649	*APOE*	Apolipoprotein E (Apo-E)	1.73	0.020
Q9BQ69	*MACROD1*	ADP-ribose glycohydrolase MACROD1 (MACRO domain-containing protein 1) (O-acetyl-ADP-ribose deacetylase MACROD1) (EC 3.1.1.106) (Protein LRP16) ([Protein ADP-ribosylaspartate] hydrolase MACROD1) (EC 3.2.2.-) ([Protein ADP-ribosylglutamate] hydrolase MACROD1) (EC 3.2.2.-)	2.25	0.021

Q9P0H9	*RER1*	Protein RER1	0.55	0.021
P60953	*CDC42*	Cell division control protein 42 homolog (EC 3.6.5.2) (G25K GTP-binding protein)	0.75	0.021
Q9Y6I9	*TEX264*	Testis-expressed protein 264 (Putative secreted protein Zsig11)	0.63	0.022
P54646	*PRKAA2*	5'-AMP-activated protein kinase catalytic subunit alpha-2 (AMPK subunit alpha-2) (EC 2.7.11.1) (Acetyl-CoA carboxylase kinase) (ACACA kinase) (EC 2.7.11.27) (Hydroxymethylglutaryl-CoA reductase kinase) (HMGCR kinase) (EC 2.7.11.31)	1.47	0.022
Q96EY7	*PTCD3*	Pentatricopeptide repeat domain-containing protein 3, mitochondrial (28S ribosomal protein S39, mitochondrial) (MRP-S39) (Mitochondrial small ribosomal subunit protein mS39) (Transformation-related gene 15 protein) (TRG-15)	1.33	0.022
Q14894	*CRYM*	Ketimine reductase mu-crystallin (EC 1.5.1.25) (NADP-regulated thyroid-hormone-binding protein)	1.52	0.022
Q9H9J2	*MRPL44*	39S ribosomal protein L44, mitochondrial (L44mt) (MRP-L44) (EC 3.1.26.-) (Mitochondrial large ribosomal subunit protein mL44)	1.37	0.022
Q96RF0	*SNX18*	Sorting nexin-18 (SH3 and PX domain-containing protein 3B) (Sorting nexin-associated Golgi protein 1)	1.61	0.023
Q5JSH3	*WDR44*	WD repeat-containing protein 44 (Rabphilin-11)	1.84	0.023
Q5TAL4	*SNRPC*	U1 small nuclear ribonucleoprotein C (U1 snRNP C) (U1-C) (U1C)	0.72	0.023
A0A5C2GF38		IG c403_heavy_IGHV3-74_IGHD4-23_IGHJ6 (IG c67_heavy_IGHV3-74_IGHD4-23_IGHJ6) (Fragment)	1.59	0.023
Q9Y666	*SLC12A7*	Solute carrier family 12 member 7 (Electroneutral potassium-chloride cotransporter 4) (K-Cl cotransporter 4)	0.75	0.023
P48739	*PITPNB*	Phosphatidylinositol transfer protein beta isoform (PI-TP-beta) (PtdIns transfer protein beta) (PtdInsTP beta)	0.64	0.024
Q9UJU6	*DBNL*	Drebrin-like protein (Cervical SH3P7) (Cervical mucin-associated protein) (Drebrin-F) (HPK1-interacting protein of 55 kDa) (HIP-55) (SH3 domain-containing protein 7)	0.73	0.024

A0A024RBH7	*TMPO*	Thymopoietin, isoform CRA_a	0.75	0.025
Q9UDW1	*UQCR10*	Cytochrome b-c1 complex subunit 9 (Complex III subunit 9) (Complex III subunit X) (Cytochrome c1 non-heme 7 kDa protein) (Ubiquinol-cytochrome c reductase complex 7.2 kDa protein)	1.41	0.025
P0DMN0	*SULT1A4*	Sulfotransferase 1A4 (ST1A4) (EC 2.8.2.1) (Aryl sulfotransferase 1A3/1A4) (Sulfotransferase 1A3/1A4)	0.64	0.025
P07711	*CTSL*	Procathepsin L (EC 3.4.22.15) (Cathepsin L1) (Major excreted protein) (MEP) [Cleaved into: Cathepsin L; Cathepsin L heavy chain; Cathepsin L light chain]	0.38	0.025
A0A024R7F4	*DNASE2*	Deoxyribonuclease II (EC 3.1.22.1)	0.72	0.025
Q9UM22	*EPDR1*	Mammalian ependymin-related protein 1 (MERP-1) (Upregulated in colorectal cancer gene 1 protein)	1.81	0.025
O95817	*BAG3*	BAG family molecular chaperone regulator 3 (BAG-3) (Bcl-2-associated athanogene 3) (Bcl-2-binding protein Bis) (Docking protein CAIR-1)	0.76	0.025
O00505	*KPNA3*	Importin subunit alpha-4 (Importin alpha Q2) (Qip2) (Karyopherin subunit alpha-3) (SRP1-gamma)	0.74	0.026
O14880	*MGST3*	Microsomal glutathione S-transferase 3 (Microsomal GST-3) (Glutathione peroxidase MGST3) (EC 1.11.1.-) (LTC4 synthase MGST3) (EC 4.4.1.20) (Microsomal glutathione S-transferase III) (Microsomal GST-III)	0.56	0.026
Q08380	*LGALS3BP*	Galectin-3-binding protein (Basement membrane autoantigen p105) (Lectin galactoside-binding soluble 3-binding protein) (Mac-2-binding protein) (MAC2BP) (Mac-2 BP) (Tumor-associated antigen 90K)	0.66	0.026
O94915	*FRYL*	Protein furry homolog-like (ALL1-fused gene from chromosome 4p12 protein)	0.73	0.026
Q5T765	*IFIT3*	Interferon-induced protein with tetratricopeptide repeats 3, isoform CRA_a (Interferon-induced protein with tetratricopeptide repeats 3, isoform CRA_b) (cDNA FLJ75638, highly similar to Homo sapiens interferon-induced protein with tetratricopeptide repeats 3 (IFIT3), mRNA)	2.46	0.026
Q8N5J2	*MINDY1*	Ubiquitin carboxyl-terminal hydrolase MINDY-1 (EC 3.4.19.12) (Deubiquitinating enzyme MINDY-1) (Protein FAM63A)	2.22	0.027

P13645	*KRT10*	Keratin, type I cytoskeletal 10 (Cytokeratin-10) (CK-10) (Keratin-10) (K10)	4.41	0.027
J3QLS3	*MRPS7*	28S ribosomal protein S7, mitochondrial	1.38	0.028
Q86TI2	*DPP9*	Dipeptidyl peptidase 9 (DP9) (EC 3.4.14.5) (Dipeptidyl peptidase IV-related protein 2) (DPRP-2) (Dipeptidyl peptidase IX) (DPP IX) (Dipeptidyl peptidase-like protein 9) (DPLP9)	1.69	0.028
Q13445	*TMED1*	Transmembrane emp24 domain-containing protein 1 (Interleukin-1 receptor-like 1 ligand) (Putative T1/ST2 receptor-binding protein) (p24 family protein gamma-1) (Tp24) (p24gamma1)	0.65	0.029
P19237	*TNNI1*	Troponin I, slow skeletal muscle (Troponin I, slow-twitch isoform)	3.24	0.029
Q9UJ68	*MSRA*	Mitochondrial peptide methionine sulfoxide reductase (EC 1.8.4.11) (Peptide-methionine (S)-S-oxide reductase) (Peptide Met(O) reductase) (Protein-methionine-S-oxide reductase) (PMSR)	1.38	0.030
Q6IC98	*GRAMD4*	GRAM domain-containing protein 4 (Death-inducing protein)	1.68	0.030
P62263	*RPS14*	40S ribosomal protein S14 (Small ribosomal subunit protein uS11)	1.55	0.030
P43121	*MCAM*	Cell surface glycoprotein MUC18 (Cell surface glycoprotein P1H12) (Melanoma cell adhesion molecule) (Melanoma-associated antigen A32) (Melanoma-associated antigen MUC18) (S-endo 1 endothelial-associated antigen) (CD antigen CD146)	0.71	0.030
O15294	*OGT*	UDP-N-acetylglucosamine--peptide N-acetylglucosaminyltransferase 110 kDa subunit (EC 2.4.1.255) (O-GlcNAc transferase subunit p110) (O-linked N-acetylglucosamine transferase 110 kDa subunit) (OGT)	3.06	0.031
A0A024R4E5	*HDLBP*	High density lipoprotein binding protein (Vigilin), isoform CRA_a (Vigilin)	0.71	0.031
Q9UIA9	*XPO7*	Exportin-7 (Exp7) (Ran-binding protein 16)	1.46	0.031
Q9UNH7	*SNX6*	Sorting nexin-6 (TRAF4-associated factor 2) [Cleaved into: Sorting nexin-6, N-terminally processed]	1.47	0.031
P02753	*RBP4*	Retinol-binding protein 4 (Plasma retinol-binding protein) (PRBP) (RBP) [Cleaved into: Plasma retinol-binding protein(1-182); Plasma retinol-binding protein(1-181); Plasma retinol-binding protein(1-179); Plasma retinol-binding protein(1-176)]	1.73	0.032
P37802	*TAGLN2*	Transgelin-2 (Epididymis tissue protein Li 7e) (SM22-alpha homolog)	0.70	0.032

Q96DH6	*MSI2*	RNA-binding protein Musashi homolog 2 (Musashi-2)	0.66	0.032
Q58WW2	*DCAF6*	DDB1- and CUL4-associated factor 6 (Androgen receptor complex-associated protein) (ARCAP) (IQ motif and WD repeat-containing protein 1) (Nuclear receptor interaction protein) (NRIP)	1.31	0.032
Q5VYK3	*ECPAS*	Proteasome adapter and scaffold protein ECM29 (Ecm29 proteasome adapter and scaffold) (Proteasome-associated protein ECM29 homolog)	1.31	0.034
G3V3D1	*NPC2*	Epididymal secretory protein E1 (NPC intracellular cholesterol transporter 2) (Fragment)	0.64	0.034
Q9BUT1	*BDH2*	3-hydroxybutyrate dehydrogenase type 2 (EC 1.1.1.-) (EC 1.1.1.30) (Dehydrogenase/reductase SDR family member 6) (Oxidoreductase UCPA) (R-beta-hydroxybutyrate dehydrogenase) (Short chain dehydrogenase/reductase family 15C member 1)	0.58	0.034
A3R0T8	*HIST1H1E*	Histone 1, H1e (Histone H1e)	0.58	0.034
A0A5C2G6J9		IGL c3415_light_IGKV1D-13_IGKJ2 (Fragment)	2.50	0.035
P78527	*PRKDC*	DNA-dependent protein kinase catalytic subunit (DNA-PK catalytic subunit) (DNA-PKcs) (EC 2.7.11.1) (DNPK1) (p460)	1.48	0.036
Q6FHG5	*SNCG*	Gamma-synuclein	0.71	0.036
Q93062	*RBPMS*	RNA-binding protein with multiple splicing (RBP-MS) (Heart and RRM expressed sequence) (Hermes)	1.40	0.037
P50336	*PPOX*	Protoporphyrinogen oxidase (PPO) (EC 1.3.3.4)	1.69	0.038
A0A024RDY0	*RANBP5*	RAN binding protein 5, isoform CRA_d	0.65	0.038
A0A024R125	*PRKAG1*	5'-AMP-activated protein kinase subunit gamma-1	0.62	0.038
P31948	*STIP1*	Stress-induced-phosphoprotein 1 (STI1) (Hsc70/Hsp90-organizing protein) (Hop) (Renal carcinoma antigen NY-REN-11) (Transformation-sensitive protein IEF SSP 3521)	0.72	0.038
P13647	*KRT5*	Keratin, type II cytoskeletal 5 (58 kDa cytokeratin) (Cytokeratin-5) (CK-5) (Keratin-5) (K5) (Type-II keratin Kb5)	3.56	0.038
Q08257	*CRYZ*	Quinone oxidoreductase (EC 1.6.5.5) (NADPH:quinone reductase) (Zeta-crystallin)	0.73	0.039

Q9Y3D3	*MRPS16*	28S ribosomal protein S16, mitochondrial (MRP-S16) (S16mt) (Mitochondrial small ribosomal subunit protein bS16m)	1.59	0.039
Q5BKX8	*CAVIN4*	Caveolae-associated protein 4 (Muscle-related coiled-coil protein) (Muscle-restricted coiled-coil protein)	0.65	0.039
P62910	*RPL32*	60S ribosomal protein L32 (Large ribosomal subunit protein eL32)	1.39	0.039
Q9BPW8	*NIPSNAP1*	Protein NipSnap homolog 1 (NipSnap1)	1.78	0.040
Q7L576	*CYFIP1*	Cytoplasmic FMR1-interacting protein 1 (Specifically Rac1-associated protein 1) (Sra-1) (p140sra-1)	1.39	0.040
Q9ULA0	*DNPEP*	Aspartyl aminopeptidase (EC 3.4.11.21)	1.32	0.040
A0A0S2Z4F1	*EFEMP1*	EGF containing fibulin-like extracellular matrix protein 1 isoform 1 (Fragment)	0.54	0.041
Q02221	*COX6A2*	Cytochrome c oxidase subunit 6A2, mitochondrial (Cytochrome c oxidase polypeptide VIa-heart) (COXVIAH) (Cytochrome c oxidase subunit VIA-muscle) (COX VIa-M)	1.98	0.042
Q9NWV4	*CZIB*	CXXC motif containing zinc binding protein (UPF0587 protein C1orf123)	0.75	0.042
O75937	*DNAJC8*	DnaJ homolog subfamily C member 8 (Splicing protein spf31)	0.64	0.042
Q969V3	*NCLN*	Nicalin (Nicastrin-like protein)	1.64	0.043
Q59EK6		TNF receptor-associated protein 1 variant (Fragment)	1.32	0.044
H0YHG0		Uncharacterized protein (Fragment)	0.72	0.044
P14672	*SLC2A4*	Solute carrier family 2, facilitated glucose transporter member 4 (Glucose transporter type 4, insulin-responsive) (GLUT-4)	0.75	0.044
Q15428	*SF3A2*	Splicing factor 3A subunit 2 (SF3a66) (Spliceosome-associated protein 62) (SAP 62)	0.59	0.045
Q8N5N7	*MRPL50*	39S ribosomal protein L50, mitochondrial (L50mt) (MRP-L50) (Mitochondrial large ribosomal subunit protein mL50)	1.79	0.045
P35612	*ADD2*	Beta-adducin (Erythrocyte adducin subunit beta)	1.76	0.045
Q9BVC6	*TMEM109*	Transmembrane protein 109 (Mitsugumin-23) (Mg23)	0.71	0.045
Q5T6L4	*ASS*	Argininosuccinate synthase (EC 6.3.4.5) (Citrulline--aspartate ligase)	1.31	0.047

Q8N8S7	*ENAH*	Protein enabled homolog	0.76	0.047
P19838	*NFKB1*	Nuclear factor NF-kappa-B p105 subunit (DNA-binding factor KBF1) (EBP-1) (Nuclear factor of kappa light polypeptide gene enhancer in B-cells 1) [Cleaved into: Nuclear factor NF-kappa-B p50 subunit]	1.40	0.048
Q9BWU5	*HBB*	Mutant hemoglobin beta chain (Fragment)	3.99	0.048
V9HVY3	*HEL-S-269*	Protein disulfide-isomerase (EC 5.3.4.1)	0.76	0.048
Q9H488	*POFUT1*	GDP-fucose protein O-fucosyltransferase 1 (EC 2.4.1.221) (Peptide-O-fucosyltransferase 1) (O-FucT-1)	0.65	0.048
Q9Y3P9	*RABGAP1*	Rab GTPase-activating protein 1 (GAP and centrosome-associated protein) (Rab6 GTPase-activating protein GAPCenA)	0.58	0.048
Q96F10	*SAT2*	Thialysine N-epsilon-acetyltransferase (EC 2.3.1.-) (Diamine acetyltransferase 2) (EC 2.3.1.57) (Spermidine/spermine N(1)-acetyltransferase 2) (SSAT-2)	1.50	0.049
Q15555	*MAPRE2*	Microtubule-associated protein RP/EB family member 2 (APC-binding protein EB2) (End-binding protein 2) (EB2)	0.72	0.049
Q9H1H9	*KIF13A*	Kinesin-like protein KIF13A (Kinesin-like protein RBKIN)	1.54	0.049

Proteins differentially (fold change >1.3 or <0.769) and significantly (P<0.05) expressed in AVS *vs* CAD pre-ischaemic cardioplegic arrest LV samples. LV, left ventricle; AVS, aortic valve stenosis; CAD, coronary artery disease.

Table SIX. Differentially expressed proteins from RV pre-ischaemic cardioplegic arrest samples between AVS and CAD patients.				
Accession no.	Gene name	Description	Fold change	P-value
A0A5C2GW15		IG c1457_light_IGKV3-20_IGKJ1 (Fragment)	0.42	0.000
Q9NRX2	*MRPL17*	39S ribosomal protein L17, mitochondrial (L17mt) (MRP-L17) (LYST-interacting protein 2) (Mitochondrial large ribosomal subunit protein bL17m)	0.52	0.000
Q6Y7W6	*GIGYF2*	GRB10-interacting GYF protein 2 (PERQ amino acid-rich with GYF domain-containing protein 2) (Trinucleotide repeat-containing gene 15 protein)	0.66	0.001
P56211	*ARPP19*	cAMP-regulated phosphoprotein 19 (ARPP-19)	1.42	0.001
P12829	*MYL4*	Myosin light chain 4 (Myosin light chain 1, embryonic muscle/atrial isoform) (Myosin light chain alkali GT-1 isoform)	8.85	0.001
C9JWC3	*SORBS2*	Sorbin and SH3 domain-containing protein 2 (Fragment)	2.75	0.001
V9HW34	*HEL-213*	Epididymis luminal protein 213	0.47	0.001
A0A5C2G6V3		IGH c144_heavy__IGHV4-39_IGHD6-6_IGHJ4 (IGH c312_heavy__IGHV4-39_IGHD6-6_IGHJ4) (IGH c3_heavy__IGHV4-39_IGHD6-6_IGHJ4) (Fragment)	0.34	0.001
A0A0G2JPR0	*C4A*	C4a anaphylatoxin (Complement C4 gamma chain)	0.23	0.002
Q6N095	*DKFZp686K03196*	Uncharacterized protein	0.57	0.002
E9PFZ2	*CP*	Ceruloplasmin	0.39	0.003
P02765	*AHSG*	Alpha-2-HS-glycoprotein (Alpha-2-Z-globulin) (Ba-alpha-2-glycoprotein) (Fetuin-A) [Cleaved into: Alpha-2-HS-glycoprotein chain A; Alpha-2-HS-glycoprotein chain B]	0.41	0.003
Q86TC9	*MYPN*	Myopalladin (145 kDa sarcomeric protein)	1.65	0.003
Q9BPW8	*NIPSNAP1*	Protein NipSnap homolog 1 (NipSnap1)	2.93	0.003
P04217	*A1BG*	Alpha-1B-glycoprotein (Alpha-1-B glycoprotein)	0.44	0.003
P02794	*FTH1*	Ferritin heavy chain (Ferritin H subunit) (EC 1.16.3.1) (Cell proliferation-inducing gene 15 protein) [Cleaved into: Ferritin heavy chain, N-terminally processed]	2.18	0.003
A0A1B0GWJ6	*MYO18A*	Unconventional myosin-XVIIIa (Fragment)	1.65	0.003

A0A5C2GB45		IGH + IGL c305_light_IGKV2-28_IGKJ1 (Fragment)	0.34	0.003
Q93062	*RBPMS*	RNA-binding protein with multiple splicing (RBP-MS) (Heart and RRM expressed sequence) (Hermes)	2.07	0.004
P49903	*SEPHS1*	Selenide, water dikinase 1 (EC 2.7.9.3) (Selenium donor protein 1) (Selenophosphate synthase 1)	0.62	0.004
A0A5C2GM29		IG c132_light_IGLV1-44_IGLJ2 (Fragment)	0.33	0.004
Q6PKG0	*LARP1*	La-related protein 1 (La ribonucleoprotein domain family member 1)	1.60	0.004
Q10713	*PMPCA*	Mitochondrial-processing peptidase subunit alpha (Alpha-MPP) (Inactive zinc metalloprotease alpha) (P-55)	1.35	0.004
P17568	*NDUFB7*	NADH dehydrogenase [ubiquinone] 1 beta subcomplex subunit 7 (Cell adhesion protein SQM1) (Complex I-B18) (CI-B18) (NADH-ubiquinone oxidoreductase B18 subunit)	1.47	0.005
P48739	*PITPNB*	Phosphatidylinositol transfer protein beta isoform (PI-TP-beta) (PtdIns transfer protein beta) (PtdInsTP beta)	0.73	0.005
P01023	*A2M*	Alpha-2-macroglobulin (Alpha-2-M) (C3 and PZP-like alpha-2-macroglobulin domain-containing protein 5)	0.28	0.005
A0A0S2Z4Z0	*RBM14*	RNA binding motif protein 14 isoform 1 (Fragment)	1.60	0.005
P02748	*C9*	Complement component C9 [Cleaved into: Complement component C9a; Complement component C9b]	0.40	0.005
A0A5C2FX10		IGL c95_light_IGLV4-69_IGLJ2 (Fragment)	0.49	0.005
P20340	*RAB6A*	Ras-related protein Rab-6A (Rab-6)	1.44	0.005
Q7L8C5	*SYT13*	Synaptotagmin-13 (Synaptotagmin XIII) (SytXIII)	0.29	0.006
O14558	*HSPB6*	Heat shock protein beta-6 (HspB6) (Heat shock 20 kDa-like protein p20)	1.95	0.006
Q6GMW4	*IGL@*	IGL@ protein	0.49	0.006
Q96K76	*USP47*	Ubiquitin carboxyl-terminal hydrolase 47 (EC 3.4.19.12) (Deubiquitinating enzyme 47) (Ubiquitin thioesterase 47) (Ubiquitin-specific-processing protease 47)	1.84	0.007

P01024	*C3*	Complement C3 (C3 and PZP-like alpha-2-macroglobulin domain-containing protein 1) [Cleaved into: Complement C3 beta chain; C3-beta-c (C3bc); Complement C3 alpha chain; C3a anaphylatoxin; Acylation stimulating protein (ASP) (C3adesArg); Complement C3b alpha' chain; Complement C3c alpha' chain fragment 1; Complement C3dg fragment; Complement C3g fragment; Complement C3d fragment; Complement C3f fragment; Complement C3c alpha' chain fragment 2]	0.51	0.007
P62263	*RPS14*	40S ribosomal protein S14 (Small ribosomal subunit protein uS11)	1.88	0.007
P0C0L5	*C4B; C4B_2*	Complement C4-B (Basic complement C4) (C3 and PZP-like alpha-2-macroglobulin domain-containing protein 3) [Cleaved into: Complement C4 beta chain; Complement C4-B alpha chain; C4a anaphylatoxin; C4b-B; C4d-B; Complement C4 gamma chain]	0.41	0.007
Q59EK6		TNF receptor-associated protein 1 variant (Fragment)	1.51	0.007
A8K008		Uncharacterized protein	0.49	0.007
P11182	*DBT*	Lipoamide acyltransferase component of branched-chain alpha-keto acid dehydrogenase complex, mitochondrial (EC 2.3.1.168) (52 kDa mitochondrial autoantigen of primary biliary cirrhosis) (Branched chain 2-oxo-acid dehydrogenase complex component E2) (BCOADC-E2) (Branched-chain alpha-keto acid dehydrogenase complex component E2) (BCKAD-E2) (BCKADE2) (Dihydrolipoamide acetyltransferase component of branched-chain alpha-keto acid dehydrogenase complex) (Dihydrolipoamide branched chain transacylase) (Dihydrolipoyllysine-residue (2-methylpropanoyl)transferase)	1.71	0.007
Q9NVA2	*SEPTIN11*	Septin-11	0.44	0.008
O43768	*ENSA*	Alpha-endosulfine (ARPP-19e)	1.59	0.008
Q9C0E8	*LNPK*	Endoplasmic reticulum junction formation protein lunapark (ER junction formation factor lunapark)	2.17	0.008
P00739	*HPR*	Haptoglobin-related protein	0.49	0.008
P62910	*RPL32*	60S ribosomal protein L32 (Large ribosomal subunit protein eL32)	1.61	0.008

E9PAV3	*NACA*	Nascent polypeptide-associated complex subunit alpha, muscle-specific form (Alpha-NAC, muscle-specific form) (skNAC)	1.43	0.008
P15924	*DSP*	Desmoplakin (DP) (250/210 kDa paraneoplastic pemphigus antigen)	1.42	0.008
P62993	*GRB2*	Growth factor receptor-bound protein 2 (Adapter protein GRB2) (Protein Ash) (SH2/SH3 adapter GRB2)	0.59	0.008
Q6IN99	*IGL@*	IGL@ protein	0.43	0.008
Q7Z460	*CLASP1*	CLIP-associating protein 1 (Cytoplasmic linker-associated protein 1) (Multiple asters homolog 1) (Protein Orbit homolog 1) (hOrbit1)	2.09	0.008
P05543	*SERPINA7*	Thyroxine-binding globulin (Serpin A7) (T4-binding globulin)	0.50	0.009
Q96BW5	*PTER*	Phosphotriesterase-related protein (EC 3.1.-.-) (Parathion hydrolase-related protein) (hPHRP)	0.62	0.009
Q86W92	*PPFIBP1*	Liprin-beta-1 (Protein tyrosine phosphatase receptor type f polypeptide-interacting protein-binding protein 1) (PTPRF-interacting protein-binding protein 1) (hSGT2)	0.72	0.009
P13796	*LCP1*	Plastin-2 (L-plastin) (LC64P) (Lymphocyte cytosolic protein 1) (LCP-1)	0.39	0.009
Q5JSH3	*WDR44*	WD repeat-containing protein 44 (Rabphilin-11)	3.28	0.009
O95671	*ASMTL*	Probable bifunctional dTTP/UTP pyrophosphatase/methyltransferase protein [Includes: dTTP/UTP pyrophosphatase (dTTPase/UTPase) (EC 3.6.1.9) (Nucleoside triphosphate pyrophosphatase) (Nucleotide pyrophosphatase) (Nucleotide PPase); N-acetylserotonin O-methyltransferase-like protein (ASMTL) (EC 2.1.1.-)]	0.46	0.010
A0A5C2G1D3		IGL c2784_light_IGKV3D-20_IGKJ3 (Fragment)	0.48	0.010
O00763	*ACACB*	Acetyl-CoA carboxylase 2 (EC 6.4.1.2) (ACC-beta)	0.26	0.010
Q5T619	*ZNF648*	Zinc finger protein 648	0.32	0.010
P08185	*SERPINA6*	Corticosteroid-binding globulin (CBG) (Serpin A6) (Transcortin)	0.46	0.010
P06213	*INSR*	Insulin receptor (IR) (EC 2.7.10.1) (CD antigen CD220) [Cleaved into: Insulin receptor subunit alpha; Insulin receptor subunit beta]	1.81	0.010

A4D2P0	*RAC1*	Ras-related C3 botulinum toxin substrate 1 (Rho family, small GTP binding protein Rac1) (Ras-related C3 botulinum toxin substrate 1 (Rho family, small GTP binding protein Rac1), isoform CRA_e) (cDNA FLJ77333, highly similar to Homo sapiens ras-related C3 botulinum toxin substrate 1 (rho family, small GTP binding protein Rac1) (RAC1), transcript variant Rac1b, mRNA)	0.62	0.010
A0A5C2FV19		IGL c612_light_IGKV3-11_IGKJ4 (Fragment)	0.51	0.010
Q9NZN4	*EHD2*	EH domain-containing protein 2 (PAST homolog 2)	0.67	0.011
Q9UIQ6	*LNPEP*	Leucyl-cystinyl aminopeptidase (Cystinyl aminopeptidase) (EC 3.4.11.3) (Insulin-regulated membrane aminopeptidase) (Insulin-responsive aminopeptidase) (IRAP) (Oxytocinase) (OTase) (Placental leucine aminopeptidase) (P-LAP) [Cleaved into: Leucyl-cystinyl aminopeptidase, pregnancy serum form]	1.54	0.011
P05164	*MPO*	Myeloperoxidase (MPO) (EC 1.11.2.2) [Cleaved into: Myeloperoxidase; 89 kDa myeloperoxidase; 84 kDa myeloperoxidase; Myeloperoxidase light chain; Myeloperoxidase heavy chain]	0.27	0.011
P35659	*DEK*	Protein DEK	1.44	0.011
P23368	*ME2*	NAD-dependent malic enzyme, mitochondrial (NAD-ME) (EC 1.1.1.38) (Malic enzyme 2)	0.60	0.011
Q92945	*KHSRP*	Far upstream element-binding protein 2 (FUSE-binding protein 2) (KH type-splicing regulatory protein) (KSRP) (p75)	0.67	0.011
P40925	*MDH1*	Malate dehydrogenase, cytoplasmic (EC 1.1.1.37) (Cytosolic malate dehydrogenase) (Diiodophenylpyruvate reductase) (EC 1.1.1.96)	1.31	0.011
P46977	*STT3A*	Dolichyl-diphosphooligosaccharide--protein glycosyltransferase subunit STT3A (Oligosaccharyl transferase subunit STT3A) (STT3-A) (EC 2.4.99.18) (B5) (Integral membrane protein 1) (Transmembrane protein TMC)	1.85	0.012
P82912	*MRPS11*	28S ribosomal protein S11, mitochondrial (MRP-S11) (S11mt) (Cervical cancer proto-oncogene 2 protein) (HCC-2) (Mitochondrial small ribosomal subunit protein uS11m)	1.40	0.012
S6BGE0		IgG H chain	0.44	0.012

Q53FA7	*TP53I3*	Quinone oxidoreductase PIG3 (EC 1.-.-.-) (Tumor protein p53-inducible protein 3) (p53-induced gene 3 protein)	0.59	0.012
A0A024R3P9	*ACBD3*	Acyl-Coenzyme A binding domain containing 3, isoform CRA_a	1.32	0.012
Q5QPK2	*DPM1*	Dolichol-phosphate mannosyltransferase subunit 1 (EC 2.4.1.83)	0.48	0.012
P30626	*SRI*	Sorcin (22 kDa protein) (CP-22) (CP22) (V19)	0.56	0.013
G4XXL9	*CYCS*	Cytochrome c	2.16	0.013
Q8IZ83	*ALDH16A1*	Aldehyde dehydrogenase family 16 member A1	0.52	0.013
Q14011	*CIRBP*	Cold-inducible RNA-binding protein (A18 hnRNP) (Glycine-rich RNA-binding protein CIRP)	0.58	0.013
Q0ZGT2	*NEXN*	Nexilin (F-actin-binding protein) (Nelin)	1.39	0.013
C0JYY2	*APOB*	Apolipoprotein B (Including Ag(X) antigen) (Apolipoprotein B (Including Ag(X) antigen), isoform CRA_a)	0.51	0.013
Q5TC12	*ATPAF1*	ATP synthase mitochondrial F1 complex assembly factor 1 (ATP11 homolog)	1.86	0.013
P67775	*PPP2CA*	Serine/threonine-protein phosphatase 2A catalytic subunit alpha isoform (PP2A-alpha) (EC 3.1.3.16) (Replication protein C) (RP-C)	1.62	0.013
P23327	*HRC*	Sarcoplasmic reticulum histidine-rich calcium-binding protein	1.91	0.013
Q86VF7	*NRAP*	Nebulin-related-anchoring protein (N-RAP)	1.62	0.014
A0A024R9Z6	*ANKMY2*	Ankyrin repeat and MYND domain containing 2, isoform CRA_b	2.06	0.014
Q9H2G2	*SLK*	STE20-like serine/threonine-protein kinase (STE20-like kinase) (hSLK) (EC 2.7.11.1) (CTCL tumor antigen se20-9) (STE20-related serine/threonine-protein kinase) (STE20-related kinase) (Serine/threonine-protein kinase 2)	1.45	0.014
Q6FGI7	*COX7A1*	COX7A1 protein (Cytochrome c oxidase subunit VIIa polypeptide 1 (Muscle)) (cDNA, FLJ92372, Homo sapiens cytochrome c oxidase subunit VIIa polypeptide 1(muscle) (COX7A1), mRNA)	0.54	0.015
P05546	*SERPIND1*	Heparin cofactor 2 (Heparin cofactor II) (HC-II) (Protease inhibitor leuserpin-2) (HLS2) (Serpin D1)	0.62	0.015
O75935	*DCTN3*	Dynactin subunit 3 (Dynactin complex subunit 22 kDa subunit) (p22)	0.74	0.015

A0A140VKA9		Testis secretory sperm-binding protein Li 236P	1.85	0.015
A0A024RBY9	*HCCS*	Cytochrome c heme lyase (EC 4.4.1.17)	2.11	0.015
P19784	*CSNK2A2*	Casein kinase II subunit alpha' (CK II alpha') (EC 2.7.11.1)	1.74	0.015
O60841	*EIF5B*	Eukaryotic translation initiation factor 5B (eIF-5B) (EC 3.6.5.3) (Translation initiation factor IF-2)	1.34	0.015
P54646	*PRKAA2*	5'-AMP-activated protein kinase catalytic subunit alpha-2 (AMPK subunit alpha-2) (EC 2.7.11.1) (Acetyl-CoA carboxylase kinase) (ACACA kinase) (EC 2.7.11.27) (Hydroxymethylglutaryl-CoA reductase kinase) (HMGCR kinase) (EC 2.7.11.31)	1.54	0.016
Q96L96	*ALPK3*	Alpha-protein kinase 3 (EC 2.7.11.1) (Muscle alpha-protein kinase)	1.93	0.016
P62861	*FAU*	40S ribosomal protein S30 (Small ribosomal subunit protein eS30)	3.57	0.016
P00738	*HP*	Haptoglobin (Zonulin) [Cleaved into: Haptoglobin alpha chain; Haptoglobin beta chain]	0.40	0.016
H0YJ34	*FERMT2*	Fermitin family homolog 2 (Fragment)	1.83	0.016
A0A024QZN9	*VDAC2*	Outer mitochondrial membrane protein porin 2 (Voltage-dependent anion-selective channel protein 2)	1.43	0.016
B4DR48		Arginyl-tRNA--protein transferase 1 (Arginyltransferase 1) (R-transferase 1) (EC 2.3.2.8) (Arginine-tRNA--protein transferase 1)	3.51	0.017
A0A1B0GUN5	*PLEKHA6*	Pleckstrin homology domain-containing family A member 6	2.43	0.017
Q16853	*AOC3*	Membrane primary amine oxidase (EC 1.4.3.21) (Copper amine oxidase) (HPAO) (Semicarbazide-sensitive amine oxidase) (SSAO) (Vascular adhesion protein 1) (VAP-1)	0.36	0.017
Q9BUR5	*APOO*	MICOS complex subunit MIC26 (Apolipoprotein O) (MICOS complex subunit MIC23) (Protein FAM121B)	1.87	0.017
Q9NX62	*BPNT2*	Golgi-resident adenosine 3',5'-bisphosphate 3'-phosphatase (Golgi-resident PAP phosphatase) (gPAPP) (EC 3.1.3.7) (3'(2'), 5'-bisphosphate nucleotidase 2) (Inositol monophosphatase domain-containing protein 1) (Myo-inositol monophosphatase A3) (Phosphoadenosine phosphate 3'-nucleotidase)	0.55	0.017
S6AWF4		IgG L chain	0.62	0.018

Q9NQP4	*PFDN4*	Prefoldin subunit 4 (Protein C-1)	1.34	0.018
A0A5C2GXF4		IG c1927_light_IGKV3-20_IGKJ4 (Fragment)	0.44	0.018
P01031	*C5*	Complement C5 (C3 and PZP-like alpha-2-macroglobulin domain-containing protein 4) [Cleaved into: Complement C5 beta chain; Complement C5 alpha chain; C5a anaphylatoxin; Complement C5 alpha' chain]	0.52	0.018
Q4ZGM8		Hemoglobin alpha-2 globin mutant (Fragment)	0.22	0.018
Q16647	*PTGIS*	Prostacyclin synthase (EC 5.3.99.4) (Hydroperoxy icosatetraenoate dehydratase) (EC 4.2.1.152) (Prostaglandin I2 synthase)	0.36	0.018
Q3MHU6	*NDUFB1*	Complex I-MNLL (NADH dehydrogenase [ubiquinone] 1 beta subcomplex subunit 1) (NADH-ubiquinone oxidoreductase MNLL subunit) (Fragment)	1.50	0.019
Q9BYV2	*TRIM54*	Tripartite motif-containing protein 54 (Muscle-specific RING finger protein) (MuRF) (Muscle-specific RING finger protein 3) (MuRF-3) (MuRF3) (RING finger protein 30)	1.46	0.019
O60240	*PLIN1*	Perilipin-1 (Lipid droplet-associated protein)	0.11	0.019
Q14624	*ITIH4*	Inter-alpha-trypsin inhibitor heavy chain H4 (ITI heavy chain H4) (ITI-HC4) (Inter-alpha-inhibitor heavy chain 4) (Inter-alpha-trypsin inhibitor family heavy chain-related protein) (IHRP) (Plasma kallikrein sensitive glycoprotein 120) (Gp120) (PK-120) [Cleaved into: 70 kDa inter-alpha-trypsin inhibitor heavy chain H4; 35 kDa inter-alpha-trypsin inhibitor heavy chain H4]	0.54	0.019
A0A024R4N0		HCG1640809, isoform CRA_b	0.27	0.019
Q92688	*ANP32B*	Acidic leucine-rich nuclear phosphoprotein 32 family member B (Acidic protein rich in leucines) (Putative HLA-DR-associated protein I-2) (PHAPI2) (Silver-stainable protein SSP29)	0.53	0.019
Q8N3K9	*CMYA5*	Cardiomyopathy-associated protein 5 (Dystrobrevin-binding protein 2) (Genethonin-3) (Myospryn) (SPRY domain-containing protein 2) (Tripartite motif-containing protein 76)	1.34	0.019
P13929	*ENO3*	Beta-enolase (EC 4.2.1.11) (2-phospho-D-glycerate hydro-lyase) (Enolase 3) (Muscle-specific enolase) (MSE) (Skeletal muscle enolase)	2.02	0.019

P08758	*ANXA5*	Annexin A5 (Anchorin CII) (Annexin V) (Annexin-5) (Calphobindin I) (CBP-I) (Endonexin II) (Lipocortin V) (Placental anticoagulant protein 4) (PP4) (Placental anticoagulant protein I) (PAP-I) (Thromboplastin inhibitor) (Vascular anticoagulant-alpha) (VAC-alpha)	0.73	0.019
Q9H9J2	*MRPL44*	39S ribosomal protein L44, mitochondrial (L44mt) (MRP-L44) (EC 3.1.26.-) (Mitochondrial large ribosomal subunit protein mL44)	1.57	0.020
Q5TZN6	*NOL3*	Nucleolar protein 3 (Apoptosis repressor with CARD domain) (Nucleolar protein 3 (Apoptosis repressor with CARD domain), isoform CRA_b) (cDNA, FLJ95803, Homo sapiens nucleolar protein 3 (apoptosis repressor with CARD domain) (NOL3), mRNA)	1.98	0.020
A0A024R4E2	*TARDBP*	TAR DNA-binding protein 43	1.39	0.020
P78527	*PRKDC*	DNA-dependent protein kinase catalytic subunit (DNA-PK catalytic subunit) (DNA-PKcs) (EC 2.7.11.1) (DNPK1) (p460)	1.57	0.020
V9HWI6	*HEL-S-51*	Gc-globulin (Group-specific component) (Vitamin D-binding protein)	0.49	0.020
Q8NF17	*FLJ00385*	FLJ00385 protein (Fragment)	0.47	0.020
P0DMN0	*SULT1A4*	Sulfotransferase 1A4 (ST1A4) (EC 2.8.2.1) (Aryl sulfotransferase 1A3/1A4) (Sulfotransferase 1A3/1A4)	0.54	0.020
Q16363	*LAMA4*	Laminin subunit alpha-4 (Laminin-14 subunit alpha) (Laminin-8 subunit alpha) (Laminin-9 subunit alpha)	0.62	0.020
Q92614	*MYO18A*	Unconventional myosin-XVIIIa (Molecule associated with JAK3 N-terminus) (MAJN) (Myosin containing a PDZ domain) (Surfactant protein receptor SP-R210) (SP-R210)	1.42	0.021
Q6VFQ6	*HBB*	Hemoglobin beta chain (Fragment)	0.43	0.021
A0A1L1UHR1		Voltage-dependent anion-selective channel protein 1	1.55	0.021
Q9H078	*CLPB*	Caseinolytic peptidase B protein homolog (EC 3.6.1.-) (Suppressor of potassium transport defect 3)	2.15	0.021
F5H5P2		2-oxoisovalerate dehydrogenase subunit alpha (EC 1.2.4.4) (Branched-chain alpha-keto acid dehydrogenase E1 component alpha chain)	1.58	0.021
X5DR21	*IQCE*	IQ motif containing E isoform A (Fragment)	0.36	0.021

A0A140VJK7		5'-deoxynucleotidase HDDC2 (EC 3.1.3.89) (HD domain-containing protein 2)	0.39	0.022
Q9UNF1	*MAGED2*	Melanoma-associated antigen D2 (11B6) (Breast cancer-associated gene 1 protein) (BCG-1) (Hepatocellular carcinoma-associated protein JCL-1) (MAGE-D2 antigen)	1.48	0.022
P49207	*RPL34*	60S ribosomal protein L34 (Large ribosomal subunit protein eL34)	1.51	0.022
Q8NDI1	*EHBP1*	EH domain-binding protein 1	0.67	0.022
P07711	*CTSL*	Procathepsin L (EC 3.4.22.15) (Cathepsin L1) (Major excreted protein) (MEP) [Cleaved into: Cathepsin L; Cathepsin L heavy chain; Cathepsin L light chain]	0.37	0.022
Q6UVK1	*CSPG4*	Chondroitin sulfate proteoglycan 4 (Chondroitin sulfate proteoglycan NG2) (Melanoma chondroitin sulfate proteoglycan) (Melanoma-associated chondroitin sulfate proteoglycan)	0.64	0.022
P04083	*ANXA1*	Annexin A1 (Annexin I) (Annexin-1) (Calpactin II) (Calpactin-2) (Chromobindin-9) (Lipocortin I) (Phospholipase A2 inhibitory protein) (p35)	0.56	0.022
A0A0F7KYT8	*FXR1*	Fragile X mental retardation autosomal homolog variant p2K (Fragile X mental retardation, autosomal homolog 1, isoform CRA_g)	0.63	0.023
O75431	*MTX2*	Metaxin-2 (Mitochondrial outer membrane import complex protein 2)	1.69	0.023
K7ELL7	*PRKCSH*	Glucosidase 2 subunit beta (Glucosidase II subunit beta)	1.34	0.023
O00273	*DFFA*	DNA fragmentation factor subunit alpha (DNA fragmentation factor 45 kDa subunit) (DFF-45) (Inhibitor of CAD) (ICAD)	1.95	0.023
Q9ULA0	*DNPEP*	Aspartyl aminopeptidase (EC 3.4.11.21)	1.53	0.024
P02679	*FGG*	Fibrinogen gamma chain	0.49	0.024
Q96BR5	*COA7*	Cytochrome c oxidase assembly factor 7 (Beta-lactamase hcp-like protein) (Respiratory chain assembly factor 1) (Sel1 repeat-containing protein 1)	1.64	0.024
E9PGN7	*SERPING1*	Plasma protease C1 inhibitor	0.48	0.024
O75323	*NIPSNAP2*	Protein NipSnap homolog 2 (NipSnap2) (Glioblastoma-amplified sequence)	1.53	0.024
J3KNB4	*CAMP*	Antibacterial peptide FALL-39 (Antibacterial peptide LL-37) (Cathelicidin antimicrobial peptide) (FALL-39 peptide antibiotic)	0.23	0.024

A0N5G3	*V-lambda-3*	Rheumatoid factor G9 light chain (Fragment)	0.46	0.024
Q6ZP82	*CCDC141*	Coiled-coil domain-containing protein 141 (Coiled-coil protein associated with myosin II and DISC1)	1.48	0.024
Q02750	*MAP2K1*	Dual specificity mitogen-activated protein kinase kinase 1 (MAP kinase kinase 1) (MAPKK 1) (MKK1) (EC 2.7.12.2) (ERK activator kinase 1) (MAPK/ERK kinase 1) (MEK 1)	0.65	0.024
Q53H26		Beta-1 metal-binding globulin (Serotransferrin) (Siderophilin) (Fragment)	0.40	0.025
Q99541	*PLIN2*	Perilipin-2 (Adipophilin) (Adipose differentiation-related protein) (ADRP)	1.86	0.025
P38159	*RBMX*	RNA-binding motif protein, X chromosome (Glycoprotein p43) (Heterogeneous nuclear ribonucleoprotein G) (hnRNP G) [Cleaved into: RNA-binding motif protein, X chromosome, N-terminally processed]	0.64	0.025
Q8NCL6		cDNA FLJ90170 fis, clone MAMMA1000370, highly similar to Ig alpha-1 chain C region	0.37	0.025
P20774	*OGN*	Mimecan (Osteoglycin) (Osteoinductive factor) (OIF)	0.36	0.025
A0A024R6I7	*SERPINA1*	Alpha-1-antitrypsin (Serpin peptidase inhibitor, clade A (Alpha-1 antiproteinase, antitrypsin), member 1, isoform CRA_a)	0.47	0.025
Q99985	*SEMA3C*	Semaphorin-3C (Semaphorin-E) (Sema E)	0.45	0.026
Q9HD42	*CHMP1A*	Charged multivesicular body protein 1a (Chromatin-modifying protein 1a) (CHMP1a) (Vacuolar protein sorting-associated protein 46-1) (Vps46-1) (hVps46-1)	1.36	0.026
A0A5C2GRP4		IG c301_heavy_IGHV4-4_IGHD2-15_IGHJ6 (Fragment)	0.34	0.026
Q8IYB8	*SUPV3L1*	ATP-dependent RNA helicase SUPV3L1, mitochondrial (EC 3.6.4.13) (Suppressor of var1 3-like protein 1) (SUV3-like protein 1)	1.76	0.026
O14925	*TIMM23*	Mitochondrial import inner membrane translocase subunit Tim23	1.46	0.026
U3KX66	*ATP8*	ATP synthase protein 8	1.43	0.027
O95197	*RTN3*	Reticulon-3 (Homolog of ASY protein) (HAP) (Neuroendocrine-specific protein-like 2) (NSP-like protein 2) (Neuroendocrine-specific protein-like II) (NSP-like protein II) (NSPLII)	0.56	0.027
A0A5C2G0Y6		IGL c2921_light_IGLV1-44_IGLJ2 (Fragment)	0.41	0.027

Q9BSF0	*C2orf88*	Small membrane A-kinase anchor protein (Small membrane AKAP) (smAKAP)	1.94	0.027
B4E1Z4		C3/C5 convertase (EC 3.4.21.47) (Complement factor B) (Complement factor B Ba fragment) (Complement factor B Bb fragment)	0.58	0.027
O60662	*KLHL41*	Kelch-like protein 41 (Kel-like protein 23) (Kelch repeat and BTB domain-containing protein 10) (Kelch-related protein 1) (Sarcosin)	1.90	0.028
O94760	*DDAH1*	N(G),N(G)-dimethylarginine dimethylaminohydrolase 1 (DDAH-1) (Dimethylarginine dimethylaminohydrolase 1) (EC 3.5.3.18) (DDAHI) (Dimethylargininase-1)	1.65	0.028
A0A096WXL7	*ATP8*	ATP synthase protein 8	1.95	0.028
A0A024R9W8	*SLC30A9*	Solute carrier family 30 member 9 (Zinc transporter 9)	2.98	0.029
Q14161	*GIT2*	ARF GTPase-activating protein GIT2 (ARF GAP GIT2) (Cool-interacting tyrosine-phosphorylated protein 2) (CAT-2) (CAT2) (G protein-coupled receptor kinase-interactor 2) (GRK-interacting protein 2)	1.47	0.030
A0A0S2Z4T8	*UROS*	Hydroxymethylbilane hydrolyase [cyclizing] (EC 4.2.1.75) (Uroporphyrinogen-III cosynthase) (Fragment)	1.50	0.030
P08311	*CTSG*	Cathepsin G (CG) (EC 3.4.21.20)	0.37	0.030
P55196	*AFDN*	Afadin (ALL1-fused gene from chromosome 6 protein) (Protein AF-6) (Afadin adherens junction formation factor)	1.82	0.031
Q14515	*SPARCL1*	SPARC-like protein 1 (High endothelial venule protein) (Hevin) (MAST 9)	0.48	0.031
A0A140VK93	*AK2*	Adenylate kinase 2, mitochondrial (AK 2) (EC 2.7.4.3) (ATP-AMP transphosphorylase 2) (ATP:AMP phosphotransferase) (Adenylate monophosphate kinase)	1.30	0.031
P62750	*RPL23A*	60S ribosomal protein L23a (Large ribosomal subunit protein uL23)	1.39	0.031
P52907	*CAPZA1*	F-actin-capping protein subunit alpha-1 (CapZ alpha-1)	0.44	0.032
O95573	*ACSL3*	Long-chain-fatty-acid--CoA ligase 3 (EC 6.2.1.3) (Arachidonate--CoA ligase) (EC 6.2.1.15) (Long-chain acyl-CoA synthetase 3) (LACS 3)	1.89	0.032

Q4U2R6	*MRPL51*	39S ribosomal protein L51, mitochondrial (L51mt) (MRP-L51) (Mitochondrial large ribosomal subunit protein mL51) (bMRP-64) (bMRP64)	2.46	0.032
B7Z6P1		cDNA FLJ53662, highly similar to Actin, alpha skeletal muscle	1.96	0.032
S6C4S0		IgG H chain	0.49	0.033
A0A5C2FYZ3		IGL c875_light_IGKV3-20_IGKJ1 (Fragment)	0.50	0.033
P27169	*PON1*	Serum paraoxonase/arylesterase 1 (PON 1) (EC 3.1.1.2) (EC 3.1.1.81) (EC 3.1.8.1) (Aromatic esterase 1) (A-esterase 1) (K-45) (Serum aryldialkylphosphatase 1)	0.39	0.033
F8VQZ7	*METAP2*	Methionine aminopeptidase 2 (MAP 2) (MetAP 2) (EC 3.4.11.18) (Initiation factor 2-associated 67 kDa glycoprotein) (Peptidase M) (p67) (p67eIF2)	1.51	0.033
A0A384NL00		Glucose-6-phosphate 1-dehydrogenase (EC 1.1.1.49)	0.49	0.034
O60506	*SYNCRIP*	Heterogeneous nuclear ribonucleoprotein Q (hnRNP Q) (Glycine- and tyrosine-rich RNA-binding protein) (GRY-RBP) (NS1-associated protein 1) (Synaptotagmin-binding, cytoplasmic RNA-interacting protein)	0.68	0.034
A0A0S2Z421	*MYOC*	Myocilin (Myocilin 20 kDa N-terminal fragment) (Myocilin 35 kDa N-terminal fragment) (Myocilin, C-terminal fragment) (Myocilin, N-terminal fragment) (Trabecular meshwork-induced glucocorticoid response protein) (Fragment)	0.60	0.034
A0A096LPE2	*SAA2-SAA4*	SAA2-SAA4 readthrough	0.49	0.034
Q6IAT9	*PSMB6*	Proteasome subunit beta (EC 3.4.25.1)	2.34	0.034
Q01105	*SET*	Protein SET (HLA-DR-associated protein II) (Inhibitor of granzyme A-activated DNase) (IGAAD) (PHAPII) (Phosphatase 2A inhibitor I2PP2A) (I-2PP2A) (Template-activating factor I) (TAF-I)	2.14	0.035
Q15572	*TAF1C*	TATA box-binding protein-associated factor RNA polymerase I subunit C (RNA polymerase I-specific TBP-associated factor 110 kDa) (TAFI110) (TATA box-binding protein-associated factor 1C) (TBP-associated factor 1C) (Transcription initiation factor SL1/TIF-IB subunit C)	0.54	0.035
Q9UJ68	*MSRA*	Mitochondrial peptide methionine sulfoxide reductase (EC 1.8.4.11) (Peptide-methionine (S)-S-oxide reductase) (Peptide Met(O) reductase) (Protein-methionine-S-oxide reductase) (PMSR)	1.62	0.035

Q6N093	*DKFZp686I04196*	Uncharacterized protein DKFZp686I04196 (Fragment)	0.47	0.035
Q99417	*MYCBP*	c-Myc-binding protein (Associate of Myc 1) (AMY-1)	1.45	0.035
P36507	*MAP2K2*	Dual specificity mitogen-activated protein kinase kinase 2 (MAP kinase kinase 2) (MAPKK 2) (EC 2.7.12.2) (ERK activator kinase 2) (MAPK/ERK kinase 2) (MEK 2)	0.77	0.035
Q9BQ69	*MACROD1*	ADP-ribose glycohydrolase MACROD1 (MACRO domain-containing protein 1) (O-acetyl-ADP-ribose deacetylase MACROD1) (EC 3.1.1.106) (Protein LRP16) ([Protein ADP-ribosylaspartate] hydrolase MACROD1) (EC 3.2.2.-) ([Protein ADP-ribosylglutamate] hydrolase MACROD1) (EC 3.2.2.-)	3.12	0.036
P41208	*CETN2*	Centrin-2 (Caltractin isoform 1)	0.33	0.036
A0A0S2Z4V6	*WFS1*	Wolfram syndrome 1 isoform 1 (Fragment)	1.66	0.036
P09871	*C1S*	Complement C1s subcomponent (EC 3.4.21.42) (C1 esterase) (Complement component 1 subcomponent s) [Cleaved into: Complement C1s subcomponent heavy chain; Complement C1s subcomponent light chain]	0.51	0.036
P23193	*TCEA1*	Transcription elongation factor A protein 1 (Transcription elongation factor S-II protein 1) (Transcription elongation factor TFIIS.o)	2.19	0.036
Q63HR2	*TNS2*	Tensin-2 (EC 3.1.3.48) (C1 domain-containing phosphatase and tensin homolog) (C1-TEN) (Tensin-like C1 domain-containing phosphatase)	0.38	0.036
P62834	*RAP1A*	Ras-related protein Rap-1A (EC 3.6.5.2) (C21KG) (G-22K) (GTP-binding protein smg p21A) (Ras-related protein Krev-1)	0.70	0.037
P61163	*ACTR1A*	Alpha-centractin (Centractin) (ARP1) (Actin-RPV) (Centrosome-associated actin homolog)	1.45	0.038
Q6ZVM7	*TOM1L2*	TOM1-like protein 2 (Target of Myb-like protein 2)	1.49	0.038
Q9P015	*MRPL15*	39S ribosomal protein L15, mitochondrial (L15mt) (MRP-L15) (Mitochondrial large ribosomal subunit protein uL15m)	1.74	0.038
P02675	*FGB*	Fibrinogen beta chain [Cleaved into: Fibrinopeptide B; Fibrinogen beta chain]	0.49	0.038

P25787	*PSMA2*	Proteasome subunit alpha type-2 (Macropain subunit C3) (Multicatalytic endopeptidase complex subunit C3) (Proteasome component C3)	2.95	0.038
Q9BTE3	*MCMBP*	Mini-chromosome maintenance complex-binding protein (MCM-BP) (MCM-binding protein)	0.24	0.038
A0A024R713	*DLD*	Dihydrolipoyl dehydrogenase (EC 1.8.1.4)	1.46	0.038
A0A0M4G3H8	*AGL*	4-alpha-glucanotransferase (EC 2.4.1.25) (EC 3.2.1.33) (Amylo-alpha-1,6-glucosidase) (Dextrin 6-alpha-D-glucosidase) (Glycogen debrancher) (Glycogen debranching enzyme) (Oligo-1,4-1,4-glucantransferase)	1.53	0.038
P80188	*LCN2*	Neutrophil gelatinase-associated lipocalin (NGAL) (25 kDa alpha-2-microglobulin-related subunit of MMP-9) (Lipocalin-2) (Oncogene 24p3) (Siderocalin) (p25)	0.34	0.038
B7Z6G2		cDNA FLJ56152, highly similar to Rho guanine nucleotide exchange factor 7	1.37	0.039
P80511	*S100A12*	Protein S100-A12 (CGRP) (Calcium-binding protein in amniotic fluid 1) (CAAF1) (Calgranulin-C) (CAGC) (Extracellular newly identified RAGE-binding protein) (EN-RAGE) (Migration inhibitory factor-related protein 6) (MRP-6) (p6) (Neutrophil S100 protein) (S100 calcium-binding protein A12) [Cleaved into: Calcitermin]	0.30	0.039
P23141	*CES1*	Liver carboxylesterase 1 (Acyl-coenzyme A:cholesterol acyltransferase) (ACAT) (Brain carboxylesterase hBr1) (Carboxylesterase 1) (CE-1) (hCE-1) (EC 3.1.1.1) (Cholesteryl ester hydrolase) (CEH) (EC 3.1.1.13) (Cocaine carboxylesterase) (Egasyn) (HMSE) (Methylumbelliferyl-acetate deacetylase 1) (EC 3.1.1.56) (Monocyte/macrophage serine esterase) (Retinyl ester hydrolase) (REH) (Serine esterase 1) (Triacylglycerol hydrolase) (TGH)	0.58	0.040
Q6ZMU0		Delta-aminolevulinic acid dehydratase (EC 4.2.1.24)	0.62	0.040
P26447	*S100A4*	Protein S100-A4 (Calvasculin) (Metastasin) (Placental calcium-binding protein) (Protein Mts1) (S100 calcium-binding protein A4)	0.60	0.040
P12429	*ANXA3*	Annexin A3 (35-alpha calcimedin) (Annexin III) (Annexin-3) (Inositol 1,2-cyclic phosphate 2-phosphohydrolase) (Lipocortin III) (Placental anticoagulant protein III) (PAP-III)	0.74	0.040

O75746	*SLC25A12*	Calcium-binding mitochondrial carrier protein Aralar1 (Mitochondrial aspartate glutamate carrier 1) (Solute carrier family 25 member 12)	1.32	0.040
P02768	*ALB*	Albumin	0.54	0.040
Q6N092	*DKFZp686K18196*	Uncharacterized protein DKFZp686K18196 (Fragment)	0.50	0.040
A0A024R125	*PRKAG1*	5'-AMP-activated protein kinase subunit gamma-1	0.53	0.040
A0A140VJP2		Methionine adenosyltransferase 2 subunit beta (Methionine adenosyltransferase II beta)	1.57	0.041
A0A024R3T8	*PARP1*	Poly [ADP-ribose] polymerase (EC 2.4.2.30)	1.34	0.041
A0A5C2GTA0		IG c684_light_IGKV3-15_IGKJ1 (Fragment)	0.62	0.041
Q8N3L3	*TXLNB*	Beta-taxilin (Muscle-derived protein 77) (hMDP77)	1.67	0.041
Q9P0H9	*RER1*	Protein RER1	0.45	0.042
P19429	*TNNI3*	Troponin I, cardiac muscle (Cardiac troponin I)	1.62	0.042
B3KML9		Delta-tubulin (Tubulin delta chain)	1.32	0.042
Q8IW45	*NAXD*	ATP-dependent (S)-NAD(P)H-hydrate dehydratase (EC 4.2.1.93) (ATP-dependent NAD(P)HX dehydratase) (Carbohydrate kinase domain-containing protein) (NAD(P)HX dehydratase)	1.38	0.042
Q14894	*CRYM*	Ketimine reductase mu-crystallin (EC 1.5.1.25) (NADP-regulated thyroid-hormone-binding protein)	2.22	0.042
P13533	*MYH6*	Myosin-6 (Myosin heavy chain 6) (Myosin heavy chain, cardiac muscle alpha isoform) (MyHC-alpha)	1.82	0.042
B4DUQ1		Heterogeneous nuclear ribonucleoprotein K	0.75	0.042
B2R9E5		cDNA, FLJ94353, highly similar to Homo sapiens apolipoprotein L, 1 (APOL1), transcript variant 2, mRNA	0.53	0.042
P50895	*BCAM*	Basal cell adhesion molecule (Auberger B antigen) (B-CAM cell surface glycoprotein) (F8/G253 antigen) (Lutheran antigen) (Lutheran blood group glycoprotein) (CD antigen CD239)	0.66	0.043

O14949	*UQCRQ*	Cytochrome b-c1 complex subunit 8 (Complex III subunit 8) (Complex III subunit VIII) (Ubiquinol-cytochrome c reductase complex 9.5 kDa protein) (Ubiquinol-cytochrome c reductase complex ubiquinone-binding protein QP-C)	1.36	0.043
P05362	*ICAM1*	Intercellular adhesion molecule 1 (ICAM-1) (Major group rhinovirus receptor) (CD antigen CD54)	0.37	0.043
P02790	*HPX*	Hemopexin (Beta-1B-glycoprotein)	0.61	0.044
P61626	*LYZ*	Lysozyme C (EC 3.2.1.17) (1,4-beta-N-acetylmuramidase C)	0.28	0.044
Q9GZZ9	*UBA5*	Ubiquitin-like modifier-activating enzyme 5 (Ubiquitin-activating enzyme 5) (ThiFP1) (UFM1-activating enzyme) (Ubiquitin-activating enzyme E1 domain-containing protein 1)	3.86	0.044
Q13557	*CAMK2D*	Calcium/calmodulin-dependent protein kinase type II subunit delta (CaM kinase II subunit delta) (CaMK-II subunit delta) (EC 2.7.11.17)	1.60	0.044
Q02487	*DSC2*	Desmocollin-2 (Cadherin family member 2) (Desmocollin-3) (Desmosomal glycoprotein II) (Desmosomal glycoprotein III)	2.00	0.044
P28827	*PTPRM*	Receptor-type tyrosine-protein phosphatase mu (Protein-tyrosine phosphatase mu) (R-PTP-mu) (EC 3.1.3.48)	1.47	0.044
Q9UDW1	*UQCR10*	Cytochrome b-c1 complex subunit 9 (Complex III subunit 9) (Complex III subunit X) (Cytochrome c1 non-heme 7 kDa protein) (Ubiquinol-cytochrome c reductase complex 7.2 kDa protein)	1.55	0.044
Q13061	*TRDN*	Triadin	2.40	0.044
A0A0S2Z2Z6	*ANXA6*	Annexin (Fragment)	0.74	0.045
P50336	*PPOX*	Protoporphyrinogen oxidase (PPO) (EC 1.3.3.4)	1.55	0.045
P0DOX7		Immunoglobulin kappa light chain (Immunoglobulin kappa light chain EU)	0.44	0.045
L0R6Q1	*SLC35A4*	SLC35A4 upstream open reading frame protein	1.37	0.046
Q96A35	*MRPL24*	39S ribosomal protein L24, mitochondrial (L24mt) (MRP-L24) (Mitochondrial large ribosomal subunit protein uL24m)	1.87	0.046
P06737	*PYGL*	Glycogen phosphorylase, liver form (EC 2.4.1.1)	0.51	0.046
Q8N465	*D2HGDH*	D-2-hydroxyglutarate dehydrogenase, mitochondrial (EC 1.1.99.-)	1.65	0.046

Q9P2R7	*SUCLA2*	Succinate--CoA ligase [ADP-forming] subunit beta, mitochondrial (EC 6.2.1.5) (ATP-specific succinyl-CoA synthetase subunit beta) (A-SCS) (Succinyl-CoA synthetase beta-A chain) (SCS-betaA)	1.32	0.047
P04003	*C4BPA*	C4b-binding protein alpha chain (C4bp) (Proline-rich protein) (PRP)	0.41	0.047
Q08AM6	*VAC14*	Protein VAC14 homolog (Tax1-binding protein 2)	0.33	0.047
P08246	*ELANE*	Neutrophil elastase (EC 3.4.21.37) (Bone marrow serine protease) (Elastase-2) (Human leukocyte elastase) (HLE) (Medullasin) (PMN elastase)	0.18	0.048
A0A5C2G2W1		IGL c2806_light_IGLV3-1_IGLJ1 (Fragment)	0.64	0.048
Q9BYT8	*NLN*	Neurolysin, mitochondrial (EC 3.4.24.16) (Angiotensin-binding protein) (Microsomal endopeptidase) (MEP) (Mitochondrial oligopeptidase M) (Neurotensin endopeptidase)	0.62	0.048
P10515	*DLAT*	Dihydrolipoyllysine-residue acetyltransferase component of pyruvate dehydrogenase complex, mitochondrial (EC 2.3.1.12) (70 kDa mitochondrial autoantigen of primary biliary cirrhosis) (PBC) (Dihydrolipoamide acetyltransferase component of pyruvate dehydrogenase complex) (M2 antigen complex 70 kDa subunit) (Pyruvate dehydrogenase complex component E2) (PDC-E2) (PDCE2)	1.42	0.048
B4DPC0		cDNA FLJ52713, moderately similar to Mus musculus leucine rich repeat (in FLII) interacting protein 1 (Lrrfip1), mRNA	1.84	0.048
P50991	*CCT4*	T-complex protein 1 subunit delta (TCP-1-delta) (CCT-delta) (Stimulator of TAR RNA-binding)	1.30	0.048
A0A3B3IRT3	*TBC1D4*	TBC1 domain family member 4	0.55	0.048
P17813	*ENG*	Endoglin (CD antigen CD105)	0.76	0.049
A0A5C2GRK1		IG c114_light_IGKV3-20_IGKJ1 (Fragment)	0.37	0.049
Q9H4A4	*RNPEP*	Aminopeptidase B (AP-B) (EC 3.4.11.6) (Arginine aminopeptidase) (Arginyl aminopeptidase)	1.81	0.049
A0A024R7L8	*UPF1*	UPF1 regulator of nonsense transcripts homolog (Yeast), isoform CRA_a	1.43	0.049
V9HW83	*HEL-S-53e*	Aldehyde dehydrogenase 1 family, member A1, isoform CRA_a (Epididymis luminal protein 12) (Epididymis secretory sperm binding protein Li 53e)	0.54	0.049
P51884	*LUM*	Lumican (Keratan sulfate proteoglycan lumican) (KSPG lumican)	0.45	0.050

Proteins differentially (fold change >1.3 or <0.769) and significantly ($P<0.05$) expressed in AVS vs. CAD pre-ischaemic cardioplegic arrest RV samples. RV, right ventricle; AVS, aortic valve stenosis; CAD, coronary artery disease.

Table SX. Significantly enriched canonical pathways for the total protein analysis between AVS and CAD patients for pre-ischaemic cardioplegic arrest samples in the LV and RV.			
	Ingenuity canonical pathway	P-value of overlap	Molecules
LV	Phospholipase C Signaling	4.57E-05	ADCY9, AHNAK, ARHGEF17, CDC42, GNAQ, GNB1, GNB4, GPLD1, GRB2, HDAC1, ITGA1, ITGA5, ITGB1, MAP2K1, MAP2K2, MAPK3, MARCKS, NFKB1, RAP1A, RHOA, RHOC, RRAS
	Gαs Signaling	6.92E-05	ADCY9, ADD2, GNB1, GNB4, MAP2K1, MAP2K2, MAPK3, PRKAG1, PRKAR1A, RAP1A
	TREM1 Signaling	1.23E-04	GRB2, ITGA5, ITGB1, MAPK3, MPO, NFKB1, STAT5B
	Neuregulin Signaling	1.86E-04	BAD, CDKN1B, GRB2, HSP90AA1, ITGA1, ITGA5, ITGB1, MAP2K1, MAP2K2, MAPK3, RAP1A, RRAS, STAT5B
	Regulation of Cellular Mechanics by Calpain Protease	1.91E-04	CAPNS1, CAST, CDK4, CDKN1B, GRB2, ITGA1, ITGA5, ITGB1, MAPK3, RAP1A, RRAS
	Chronic Myeloid Leukemia Signaling	2.57E-04	BAD, CDK4, CDKN1B, GRB2, HDAC1, MAP2K1, MAP2K2, MAPK3, NFKB1, RAP1A, RRAS, STAT5B
	Actin Nucleation by ARP-WASP Complex	3.09E-04	ARPC2, CDC42, GRB2, ITGA1, ITGA5, ITGB1, NCK2, PPP1R12C, RAP1A, RHOA, RHOC, RRAS
	Prostate Cancer Signaling	3.47E-04	BAD, CDKN1B, GRB2, GSTP1, HSP90AA1, MAP2K1, MAP2K2, MAPK3, NFKB1, RAP1A, RRAS
	JAK/Stat Signaling	3.80E-04	GNAQ, GRB2, MAP2K1, MAP2K2, MAPK3, NFKB1, PTPN1, RAP1A, RRAS, STAT5B
	α-Adrenergic Signaling	5.13E-04	ADCY9, GNAQ, GNB1, GNB4, MAP2K1, MAP2K2, MAPK3, PHKG1, PRKAG1, PRKAR1A, RAP1A, RRAS
	PI3K/AKT Signaling	6.61E-04	BAD, CDKN1B, GRB2, HSP90AA1, ITGA1, ITGA5, ITGB1, MAP2K1, MAP2K2, MAPK3, NFKB1, PPP2R5A, RAP1A, RRAS, YWHAB, YWHAG
	P2Y Purigenic Receptor Signaling Pathway	6.76E-04	ADCY9, GNAQ, GNB1, GNB4, MAP2K1, MAP2K2, MAPK3, NFKB1, PDIA3, PRKAG1, PRKAR1A, RAP1A, RRAS
	Breast Cancer Regulation by Stathmin1	7.08E-04	ARHGEF17, CDK4, CDKN1B, GNAQ, GNB1, GNB4, GPR107, GRB2, MAP2K1, MAP2K2, MAPK3, NFKB1, PPP2R5A, PRKAG1, PRKAR1A, RAP1A, RHOA, RRAS, TUBA1C, TUBA8
	ErbB2-ErbB3 Signaling	7.59E-04	BAD, CDKN1B, GRB2, MAP2K1, MAP2K2, MAPK3, RAP1A, RRAS, STAT5B

	Colorectal Cancer Metastasis Signaling	8.51E-04	ADCY9, BAD, CDC42, GNB1, GNB4, GRB2, MAP2K1, MAP2K2, MAPK3, NFKB1, PRKAG1, PRKAR1A, RAP1A, RHOA, RHOC, RRAS
	Oncostatin M Signaling	9.12E-04	GRB2, MAP2K1, MAP2K2, MAPK3, RAP1A, RRAS, STAT5B
	Pancreatic Adenocarcinoma Signaling	1.00E-03	BAD, CDC42, CDK4, CDKN1B, GPLD1, GRB2, MAP2K1, MAP2K2, MAPK3, NFKB1
	GNRH Signaling	1.07E-03	ADCY9, CACNA2D1, CDC42, GNAQ, GNB1, GRB2, MAP2K1, MAP2K2, MAPK3, NFKB1, PRKAG1, PRKAR1A, RAP1A, RRAS
	BMP signaling pathway	1.38E-03	GRB2, MAP2K1, MAP2K2, MAPK3, NFKB1, PRKAG1, PRKAR1A, RAP1A, RRAS
	PPAR Signaling	1.38E-03	GRB2, HSP90AA1, MAP2K1, MAP2K2, MAPK3, NFKB1, RAP1A, RRAS, STAT5B
RV	Acute Phase Response Signaling	7.76E-10	A2M, AHSG, ALB, C1S, C2, C3, C4BPA, C5, C9, CP, FGB, FGG, GRB2, HNRNPK, HP, HPX, ITIH4, MAP2K1, MAP2K2, RAF1, RAP1A, SAA2-SAA4, SERPINA1, SERPIND1, SERPING1
	LXR/RXR Activation	6.17E-06	A1BG, AHSG, ALB, APOB, C3, C9, HADH, HPR, HPX, ITIH4, KNG1, LYZ, PON1, SERPINA1
	Granzyme B Signaling	1.51E-05	CASP3, CYCS, DFFA, LMNB1, PARP1, PRKDC
	Acetyl-CoA Biosynthesis I (Pyruvate Dehydrogenase Complex)	5.89E-05	DBT, DLAT, DLD, PDHB
	FXR/RXR Activation	1.00E-04	A1BG, AHSG, ALB, APOB, C3, C9, HPR, HPX, ITIH4, KNG1, PON1, SERPINA1
	Complement System	2.04E-04	C1QA, C1S, C2, C3, C4BPA, C5, C9, SERPING1
	Coagulation System	6.17E-04	A2M, F10, FGB, FGG, KNG1, SERPINA1, SERPIND1
	Tumoricidal Function of Hepatic Natural Killer Cells	1.32E-03	CASP3, CYCS, DFFA, ICAM1, LYVE1
	PPAR Signaling	2.09E-03	GRB2, INSR, MAP2K1, MAP2K2, RAF1, RAP1A, SRA1, STAT5B
	Oncostatin M Signaling	2.29E-03	GRB2, MAP2K1, MAP2K2, RAF1, RAP1A, STAT5B
	IL-2 Signaling	3.31E-03	CSNK2A2, GRB2, MAP2K1, MAP2K2, RAF1, RAP1A, STAT5B
	Xenobiotic Metabolism CAR Signaling Pathway	6.46E-03	ABCC1, ALDH16A1, ALDH1A1, ALDH1A2, GSTM1, MAP2K1, MAP2K2, PPP2CA, SRA1, SULT1A3/SULT1A4

	Sertoli Cell-Sertoli Cell Junction Signaling	8.32E-03	A2M, AFDN, EPN2, JAM3, MAP2K1, MAP2K2, PRKACA, PRKAG1, RAC1, RAF1, RAP1A, TJP1, TUBA4A
	BMP signaling pathway	8.51E-03	GRB2, MAP2K1, MAP2K2, PRKACA, PRKAG1, RAF1, RAP1A
	Heme Biosynthesis II	9.33E-03	ALAD, PPOX, UROS
	JAK/Stat Signaling	9.77E-03	GNAQ, GRB2, MAP2K1, MAP2K2, RAF1, RAP1A, STAT5B
	Melatonin Signaling	9.77E-03	CAMK2D, GNAQ, MAP2K1, MAP2K2, PRKACA, PRKAG1, RAF1
	Branched-chain α-keto acid Dehydrogenase Complex	1.02E-02	DBT, DLD
	Renin-Angiotensin Signaling	1.05E-02	GNAQ, GRB2, MAP2K1, MAP2K2, PRKACA, PRKAG1, RAC1, RAF1, RAP1A
	PFKFB4 Signaling Pathway	1.20E-02	MAP2K1, MAP2K2, PFKM, PRKACA, PRKAG1

The top 20 most significant pathways are shown for each ventricle. AVS, aortic valve stenosis; CAD, coronary artery disease; RV, right ventricle; LV, left ventricle.

Table SXI. Differentially expressed phosphoproteins from LV pre-ischaemic cardioplegic arrest samples between AVS and CAD patients.					
Accession no.	Gene name	Description	Phosphosite	Fold change	P-value
O14874	*BCKDK*	[3-methyl-2-oxobutanoate dehydrogenase [lipoamide]] kinase, mitochondrial (EC 2.7.11.4) (Branched-chain alpha-ketoacid dehydrogenase kinase) (BCKD-kinase) (BCKDHKIN)	S1(Phospho)	0.52	0.000
Q15019	*SEPTIN2*	Septin-2 (Neural precursor cell expressed developmentally down-regulated protein 5) (NEDD-5)	S9(Phospho)	0.64	0.000
Q9UKG1	*APPL1*	DCC-interacting protein 13-alpha (Dip13-alpha) (Adapter protein containing PH domain, PTB domain and leucine zipper motif 1)	S14(Phospho)	1.79	0.001
P02671	*FGA*	Fibrinogen alpha chain [Cleaved into: Fibrinopeptide A; Fibrinogen alpha chain]	S3(Phospho)	0.44	0.003
Q9H1E3	*NUCKS1*	Nuclear ubiquitous casein and cyclin-dependent kinase substrate 1 (P1)	S15(Phospho), S5(Phospho), T3(Phospho)	2.60	0.005
E9PAV3	*NACA*	Nascent polypeptide-associated complex subunit alpha, muscle-specific form (Alpha-NAC, muscle-specific form) (skNAC)	S4(Phospho)	0.55	0.006
P35749	*MYH11*	Myosin-11 (Myosin heavy chain 11) (Myosin heavy chain, smooth muscle isoform) (SMMHC)	S8(Phospho)	0.55	0.007
A0A024R1N1	*MYH9*	Myosin, heavy polypeptide 9, non-muscle, isoform CRA_a	S7(Phospho)	0.60	0.009
P06732	*CKM*	Creatine kinase M-type (EC 2.7.3.2) (Creatine kinase M chain) (Creatine phosphokinase M-type) (CPK-M) (M-CK)	S12(Phospho)	0.61	0.010
Q8TCJ2	*STT3B*	Dolichyl-diphosphooligosaccharide--protein glycosyltransferase subunit STT3B (Oligosaccharyl transferase subunit STT3B) (STT3-B) (EC 2.4.99.18) (Source of immunodominant MHC-associated peptides homolog)	S8(Phospho), S9(Phospho)	0.50	0.017

Q5VWP3	*MLIP*	Muscular LMNA-interacting protein (Cardiac Isl1-interacting protein) (CIP) (Muscular-enriched A-type laminin-interacting protein)	S9(Phospho)	0.56	0.020
B7ZA42		cDNA, FLJ79056 (Fragment)	S11(Phospho)	0.53	0.022
E7EW31	*PROB1*	Proline-rich basic protein 1	S3(Phospho)	0.72	0.023
O00505	*KPNA3*	Importin subunit alpha-4 (Importin alpha Q2) (Qip2) (Karyopherin subunit alpha-3) (SRP1-gamma)	S11(Phospho)	1.47	0.024
O14974	*PPP1R12A*	Protein phosphatase 1 regulatory subunit 12A (Myosin phosphatase-targeting subunit 1) (Myosin phosphatase target subunit 1) (Protein phosphatase myosin-binding subunit)	S20(Phospho)	0.66	0.026
B7Z2R7	*ACBD5*	Acyl-CoA-binding domain-containing protein 5	S10(Phospho), S6(Phospho)	2.68	0.026
Q9GZY8	*MFF*	Mitochondrial fission factor	S1(Phospho)	1.61	0.029
E7EUU4	*EIF4G1*	Eukaryotic translation initiation factor 4 gamma 1	S6(Phospho)	0.68	0.029
Q6FI27	*GSK3B*	Glycogen synthase kinase-3 beta (EC 2.7.11.26)	Y7(Phospho)	2.28	0.030
O00629	*KPNA4*	Importin subunit alpha-3 (Importin alpha Q1) (Qip1) (Karyopherin subunit alpha-4)	S11(Phospho)	0.47	0.035
Q96PE2	*ARHGEF17*	Rho guanine nucleotide exchange factor 17 (164 kDa Rho-specific guanine-nucleotide exchange factor) (p164-RhoGEF) (p164RhoGEF) (Tumor endothelial marker 4)	S3(Phospho)	1.63	0.036
A0A2R8Y4T1	*TNS1*	Tensin-1	S11(Phospho)	0.44	0.037
Q16851	*UGP2*	UTP--glucose-1-phosphate uridylyltransferase (EC 2.7.7.9) (UDP-glucose pyrophosphorylase) (UDPGP) (UGPase)	S3(Phospho)	0.57	0.042
Q14247	*CTTN*	Src substrate cortactin (Amplaxin) (Oncogene EMS1)	S9(Phospho), T5(Phospho)	0.55	0.045

Phosphoproteins differentially (fold change >1.3 or <0.769) and significantly ($P<0.05$) expressed in AVS vs. CAD pre surgery samples from the LV. AVS, aortic valve stenosis; CAD, coronary artery disease; LV, left ventricle.

Table SXII. Differentially expressed phosphoproteins from RV pre-ischaemic cardioplegic arrest samples between AVS and CAD patients.					
Accession no.	Gene name	Description	Phosphosite	Fold change	P-value
P08559	*PDHA1*	Pyruvate dehydrogenase E1 component subunit alpha, somatic form, mitochondrial (EC 1.2.4.1) (PDHE1-A type I)	Ambiguous,	1.89	0.001
P27824	*CANX*	Calnexin (IP90) (Major histocompatibility complex class I antigen-binding protein p88) (p90)	S3(Phospho)	0.46	0.005
C9JWC3	*SORBS2*	Sorbin and SH3 domain-containing protein 2 (Fragment)	S4(Phospho)	0.33	0.007
Q9UKG1	*APPL1*	DCC-interacting protein 13-alpha (Dip13-alpha) (Adapter protein containing PH domain, PTB domain and leucine zipper motif 1)	S14(Phospho)	1.60	0.010
Q15019	*SEPTIN2*	Septin-2 (Neural precursor cell expressed developmentally down-regulated protein 5) (NEDD-5)	S9(Phospho)	0.70	0.012
Q2M3C7	*SPHKAP*	A-kinase anchor protein SPHKAP (SPHK1-interactor and AKAP domain-containing protein) (Sphingosine kinase type 1-interacting protein)	S15(Phospho)	1.87	0.018
Q6FI27	*GSK3B*	Glycogen synthase kinase-3 beta (EC 2.7.11.26)	Y7(Phospho)	2.05	0.022
A0A1B0GTU4	*PXN*	Paxillin	S2(Phospho)	2.90	0.025
O60343	*TBC1D4*	TBC1 domain family member 4 (Akt substrate of 160 kDa) (AS160)	Ambiguous,	2.27	0.035
Q14247	*CTTN*	Src substrate cortactin (Amplaxin) (Oncogene EMS1)	S9(Phospho), T5(Phospho)	0.43	0.043
Q15772	*SPEG*	Striated muscle preferentially expressed protein kinase (EC 2.7.11.1) (Aortic preferentially expressed protein 1) (APEG-1)	S3(Phospho)	1.59	0.049
Phosphoproteins differentially (fold change >1.3 or <0.769) and significantly (P<0.05) expressed in AVS vs. CAD pre surgery samples from the RV. AVS, aortic valve stenosis; CAD, coronary artery disease; RV, right ventricle.					

Table SXIII. Significantly enriched canonical pathways for the relative phosphoprotein analysis between AVS and CAD patients for pre-ischaemic cardioplegic arrest samples in the LV and RV.

	Ingenuity canonical pathway	P-value of overlap	Molecules
LV	Cellular Effects of Sildenafil (Viagra)	1.26E-05	MYH11, MYH7, MYH9, PDE3A, PPP1R12A, PPP1R12B, PRKAR1A, PRKAR2A
	Protein Kinase A Signaling	3.24E-04	CAMK2B, FLNC, GSK3A, GSK3B, PDE3A, PHKB, PPP1R12A, PPP1R7, PRKAR1A, PRKAR2A, TTN
	Sonic Hedgehog Signaling	5.13E-04	GSK3B, PRKAR1A, PRKAR2A
	Semaphorin Neuronal Repulsive Signaling Pathway	5.75E-04	GSK3B, MAPT, PPP1R12A, PPP1R12B, PRKAR1A, PRKAR2A, VCAN
	ILK Signaling	6.61E-04	FLNC, GSK3A, GSK3B, MYH11, MYH7, MYH9, NACA, PPP1R12A
	Insulin Receptor Signaling	6.61E-04	GSK3A, GSK3B, PPP1R12A, PPP1R7, PRKAR1A, PRKAR2A, SLC2A4
	Tight Junction Signaling	1.26E-03	AFDN, MYH11, MYH7, MYH9, PRKAR1A, PRKAR2A, TJP2
	RAN Signaling	1.48E-03	KPNA3, KPNA4, RANBP2
	Actin Cytoskeleton Signaling	2.57E-03	FGF12, GIT1, MYH11, MYH7, MYH9, PPP1R12A, PPP1R12B, TTN
	Amyloid Processing	2.69E-03	GSK3B, MAPT, PRKAR1A, PRKAR2A
	Calcium Signaling	3.31E-03	CAMK2B, MYH11, MYH7, MYH9, PRKAR1A, PRKAR2A
	Dopamine Receptor Signaling	4.37E-03	PPP1R12A, PPP1R7, PRKAR1A, PRKAR2A
	CDK5 Signaling	4.57E-03	MAPT, PPP1R12A, PPP1R7, PRKAR1A, PRKAR2A
	Melatonin Signaling	4.79E-03	CAMK2B, PRKAR1A, PRKAR2A, SLC2A4
	Synaptic Long Term Potentiation	4.90E-03	CAMK2B, PPP1R12A, PPP1R7, PRKAR1A, PRKAR2A
	Sertoli Cell-Sertoli Cell Junction Signaling	7.94E-03	AFDN, GSK3A, GSK3B, PRKAR1A, PRKAR2A, TJP2
	Xenobiotic Metabolism PXR Signaling Pathway	8.91E-03	CAMK2B, PPP1R12A, PPP1R7, PRKAR1A, PRKAR2A
	Cardiac β-adrenergic Signaling	1.00E-02	PDE3A, PPP1R12A, PPP1R7, PRKAR1A, PRKAR2A
	Coronavirus Replication Pathway	1.02E-02	BAG3, GSK3A, GSK3B
	Synaptogenesis Signaling Pathway	1.26E-02	AFDN, CAMK2B, GSK3B, MAPT, MARCKS, PRKAR1A, PRKAR2A

RV	Leptin Signaling in Obesity	6.03E-04	MAPK1, PDE3A, PLCL2, PRKAR1A
	Role of PI3K/AKT Signaling in the Pathogenesis of Influenza	1.82E-03	GSK3B, KPNA3, MAPK1
	Protein Kinase A Signaling	2.51E-03	GSK3B, MAPK1, PDE3A, PLCL2, PPP1R7, PRKAR1A, PXN
	Sertoli Cell-Sertoli Cell Junction Signaling	3.47E-03	AFDN, EPN2, GSK3B, MAPK1, PRKAR1A
	Synaptic Long Term Potentiation	3.98E-03	MAPK1, PLCL2, PPP1R7, PRKAR1A
	Sonic Hedgehog Signaling	4.07E-03	GSK3B, PRKAR1A
	Amyloid Processing	4.79E-03	GSK3B, MAPK1, PRKAR1A
	Neuropathic Pain Signaling In Dorsal Horn Neurons	6.46E-03	MAPK1, PLCL2, PRKAR1A
	Agrin Interactions at Neuromuscular Junction	7.08E-03	CTTN, MAPK1, PXN
	Melatonin Signaling	7.59E-03	MAPK1, PLCL2, PRKAR1A
	RAN Signaling	8.13E-03	KPNA3, RANBP2
	IL-17A Signaling in Fibroblasts	9.33E-03	GSK3B, MAPK1
	Aldosterone Signaling in Epithelial Cells	1.00E-02	HSPA12B, HSPB6, MAPK1, PLCL2
	Insulin Receptor Signaling	1.10E-02	GSK3B, MAPK1, PPP1R7, PRKAR1A
	Leukocyte Extravasation Signaling	1.12E-02	AFDN, CTTN, MAPK1, PXN
	Germ Cell-Sertoli Cell Junction Signaling	1.26E-02	AFDN, EPN2, MAPK1, PXN
	Ovarian Cancer Signaling	1.38E-02	GSK3B, MAPK1, PRKAR1A
	Adrenomedullin signaling pathway	1.41E-02	GSK3B, MAPK1, PLCL2, PRKAR1A
	Colorectal Cancer Metastasis Signaling	1.45E-02	APPL1, GSK3B, MAPK1, PRKAR1A
	Endocannabinoid Neuronal Synapse Pathway	1.45E-02	MAPK1, PLCL2, PRKAR1A

The top 20 most significant pathways are shown for each ventricle. AVS, aortic valve stenosis; CAD, coronary artery disease; RV, right ventricle; LV, left ventricle.

Table SXIV. Differentially expressed proteins from LV post-reperfusion samples between AVS and CAD patients.				
Accession No.	Gene name	Description	Fold change	P-value
A0A0S2Z3Y7	*GALT*	Galactose-1-phosphate uridylyltransferase (EC 2.7.7.12) (Fragment)	0.65	0.000
Q7L0Y3	*TRMT10C*	tRNA methyltransferase 10 homolog C (HBV pre-S2 trans-regulated protein 2) (Mitochondrial ribonuclease P protein 1) (Mitochondrial RNase P protein 1) (RNA (guanine-9-)-methyltransferase domain-containing protein 1) (Renal carcinoma antigen NY-REN-49) (mRNA methyladenosine-N(1)-methyltransferase) (EC 2.1.1.-) (tRNA (adenine(9)-N(1))-methyltransferase) (EC 2.1.1.218) (tRNA (guanine(9)-N(1))-methyltransferase) (EC 2.1.1.221)	0.75	0.000
O14786	*NRP1*	Neuropilin-1 (Vascular endothelial cell growth factor 165 receptor) (CD antigen CD304)	1.44	0.001
Q0VAK6	*LMOD3*	Leiomodin-3 (Leiomodin, fetal form)	2.12	0.001
Q5JPE7	*NOMO2*	Nodal modulator 2 (pM5 protein 2)	0.60	0.001
M0QXB5	*ETHE1*	Persulfide dioxygenase ETHE1, mitochondrial	0.72	0.002
O95487	*SEC24B*	Protein transport protein Sec24B (SEC24-related protein B)	0.50	0.002
O00764	*PDXK*	Pyridoxal kinase (EC 2.7.1.35) (Pyridoxine kinase)	1.61	0.002
P30626	*SRI*	Sorcin (22 kDa protein) (CP-22) (CP22) (V19)	0.58	0.003
P49903	*SEPHS1*	Selenide, water dikinase 1 (EC 2.7.9.3) (Selenium donor protein 1) (Selenophosphate synthase 1)	0.65	0.003
P62993	*GRB2*	Growth factor receptor-bound protein 2 (Adapter protein GRB2) (Protein Ash) (SH2/SH3 adapter GRB2)	0.52	0.004
Q6IB54	*ATP5J*	ATP synthase-coupling factor 6, mitochondrial (ATPase subunit F6)	0.67	0.004
A0A3S6RG84	*HLA-C*	MHC class I antigen	0.58	0.004
O43815	*STRN*	Striatin	0.66	0.005
Q9BZL4	*PPP1R12C*	Protein phosphatase 1 regulatory subunit 12C (Protein phosphatase 1 myosin-binding subunit of 85 kDa) (Protein phosphatase 1 myosin-binding subunit p85)	0.54	0.005
A0A024R7U9	*ATP6V1H*	V-type proton ATPase subunit H	0.76	0.005
A0A0S2Z5D4	*DCTN4*	Dynactin 4 isoform 1 (Fragment)	0.61	0.005

O95671	*ASMTL*	Probable bifunctional dTTP/UTP pyrophosphatase/methyltransferase protein [Includes: dTTP/UTP pyrophosphatase (dTTPase/UTPase) (EC 3.6.1.9) (Nucleoside triphosphate pyrophosphatase) (Nucleotide pyrophosphatase) (Nucleotide PPase); N-acetylserotonin O-methyltransferase-like protein (ASMTL) (EC 2.1.1.-)]	0.50	0.005
A0A024RCX4	*MTCH1*	Mitochondrial carrier homolog 1 (C. elegans), isoform CRA_c	0.76	0.006
O75695	*RP2*	Protein XRP2	0.64	0.006
P05109	*S100A8*	Protein S100-A8 (Calgranulin-A) (Calprotectin L1L subunit) (Cystic fibrosis antigen) (CFAG) (Leukocyte L1 complex light chain) (Migration inhibitory factor-related protein 8) (MRP-8) (p8) (S100 calcium-binding protein A8) (Urinary stone protein band A)	0.62	0.006
A0A384NPH9		Glypican-1 (Secreted glypican-1)	0.67	0.007
Q96ER9	*CCDC51*	Mitochondrial potassium channel (MITOK) (Coiled-coil domain-containing protein 51)	0.68	0.007
A0A024R5K1	*CORO1B*	Coronin	1.81	0.008
P08648	*ITGA5*	Integrin alpha-5 (CD49 antigen-like family member E) (Fibronectin receptor subunit alpha) (Integrin alpha-F) (VLA-5) (CD antigen CD49e) [Cleaved into: Integrin alpha-5 heavy chain; Integrin alpha-5 light chain]	0.59	0.008
Q9H008	*LHPP*	Phospholysine phosphohistidine inorganic pyrophosphate phosphatase (hLHPP) (EC 3.1.3.-) (EC 3.6.1.1)	0.36	0.008
D3DNI2	*PFN2*	Profilin (Fragment)	0.57	0.008
Q9Y6I9	*TEX264*	Testis-expressed protein 264 (Putative secreted protein Zsig11)	0.65	0.009
P10643	*C7*	Complement component C7	1.90	0.009
O95881	*TXNDC12*	Thioredoxin domain-containing protein 12 (EC 1.8.4.2) (Endoplasmic reticulum resident protein 18) (ER protein 18) (ERp18) (Endoplasmic reticulum resident protein 19) (ER protein 19) (ERp19) (Thioredoxin-like protein p19) (hTLP19)	0.75	0.009
P05230	*FGF1*	Fibroblast growth factor 1 (FGF-1) (Acidic fibroblast growth factor) (aFGF) (Endothelial cell growth factor) (ECGF) (Heparin-binding growth factor 1) (HBGF-1)	0.61	0.009
A0A0F7KYT8	*FXR1*	Fragile X mental retardation autosomal homolog variant p2K (Fragile X mental retardation, autosomal homolog 1, isoform CRA_g)	0.67	0.010

A0A140VJK7		5'-deoxynucleotidase HDDC2 (EC 3.1.3.89) (HD domain-containing protein 2)	0.39	0.010
P00367	*GLUD1*	Glutamate dehydrogenase 1, mitochondrial (GDH 1) (EC 1.4.1.3)	0.70	0.012
G0Z349	*IRF6*	Interferon regulatory factor 6	0.70	0.012
Q5QPK2	*DPM1*	Dolichol-phosphate mannosyltransferase subunit 1 (EC 2.4.1.83)	0.50	0.013
P45984	*MAPK9*	Mitogen-activated protein kinase 9 (MAP kinase 9) (MAPK 9) (EC 2.7.11.24) (JNK-55) (Stress-activated protein kinase 1a) (SAPK1a) (Stress-activated protein kinase JNK2) (c-Jun N-terminal kinase 2)	1.44	0.013
P23368	*ME2*	NAD-dependent malic enzyme, mitochondrial (NAD-ME) (EC 1.1.1.38) (Malic enzyme 2)	0.61	0.013
Q6Y1H2	*HACD2*	Very-long-chain (3R)-3-hydroxyacyl-CoA dehydratase 2 (EC 4.2.1.134) (3-hydroxyacyl-CoA dehydratase 2) (HACD2) (Protein-tyrosine phosphatase-like member B)	0.62	0.013
V9HW88	*HEL-S-99n*	Calreticulin	0.76	0.014
Q13522	*PPP1R1A*	Protein phosphatase 1 regulatory subunit 1A (Protein phosphatase inhibitor 1) (I-1) (IPP-1)	0.65	0.014
Q4U2R6	*MRPL51*	39S ribosomal protein L51, mitochondrial (L51mt) (MRP-L51) (Mitochondrial large ribosomal subunit protein mL51) (bMRP-64) (bMRP64)	1.85	0.014
A0A024RCI6	*ARMCX1*	Armadillo repeat containing, X-linked 1, isoform CRA_a	0.64	0.014
Q9P299	*COPZ2*	Coatomer subunit zeta-2 (Zeta-2-coat protein) (Zeta-2 COP)	0.63	0.015
Q8TAE8	*GADD45GIP1*	Growth arrest and DNA damage-inducible proteins-interacting protein 1 (39S ribosomal protein L59, mitochondrial) (MRP-L59) (CKII beta-associating protein) (CR6-interacting factor 1) (CRIF1) (Mitochondrial large ribosomal subunit protein mL64) (Papillomavirus L2-interacting nuclear protein 1) (PLINP) (PLINP-1) (p53-responsive gene 6 protein)	0.56	0.016
P02794	*FTH1*	Ferritin heavy chain (Ferritin H subunit) (EC 1.16.3.1) (Cell proliferation-inducing gene 15 protein) [Cleaved into: Ferritin heavy chain, N-terminally processed]	1.91	0.017
Q9C0C2	*TNKS1BP1*	182 kDa tankyrase-1-binding protein	1.47	0.017
Q9P0H9	*RER1*	Protein RER1	0.63	0.017
A0A494C1K3	*GTF2I*	General transcription factor II-I	1.49	0.018
O60669	*SLC16A7*	Monocarboxylate transporter 2 (MCT 2) (Solute carrier family 16 member 7)	0.73	0.018

P43121	*MCAM*	Cell surface glycoprotein MUC18 (Cell surface glycoprotein P1H12) (Melanoma cell adhesion molecule) (Melanoma-associated antigen A32) (Melanoma-associated antigen MUC18) (S-endo 1 endothelial-associated antigen) (CD antigen CD146)	0.66	0.018
P27144	*AK4*	Adenylate kinase 4, mitochondrial (AK 4) (EC 2.7.4.10) (EC 2.7.4.6) (Adenylate kinase 3-like) (GTP:AMP phosphotransferase AK4)	0.72	0.018
H7BZJ3	*PDIA3*	Protein disulfide-isomerase A3 (Fragment)	0.67	0.019
O60488	*ACSL4*	Long-chain-fatty-acid--CoA ligase 4 (EC 6.2.1.3) (Arachidonate--CoA ligase) (EC 6.2.1.15) (Long-chain acyl-CoA synthetase 4) (LACS 4)	0.58	0.019
Q99747	*NAPG*	Gamma-soluble NSF attachment protein (SNAP-gamma) (N-ethylmaleimide-sensitive factor attachment protein gamma)	0.74	0.020
Q15599	*SLC9A3R2*	Na(+)/H(+) exchange regulatory cofactor NHE-RF2 (NHERF-2) (NHE3 kinase A regulatory protein E3KARP) (SRY-interacting protein 1) (SIP-1) (Sodium-hydrogen exchanger regulatory factor 2) (Solute carrier family 9 isoform A3 regulatory factor 2) (Tyrosine kinase activator protein 1) (TKA-1)	0.56	0.020
Q5TC12	*ATPAF1*	ATP synthase mitochondrial F1 complex assembly factor 1 (ATP11 homolog)	1.41	0.022
Q86Y82	*STX12*	Syntaxin-12	0.75	0.022
Q5VUU6	*MNDA*	Epididymis secretory sperm binding protein (Myeloid cell nuclear differentiation antigen, isoform CRA_a) (cDNA FLJ78191, highly similar to Homo sapiens myeloid cell nuclear differentiation antigen, mRNA)	0.38	0.022
Q8N5G0	*SMIM20*	Small integral membrane protein 20 (Mitochondrial translation regulation assembly intermediate of cytochrome c oxidase protein of 7 kDa) (MITRAC7) [Cleaved into: Phoenixin-14 (PNX-14); Phoenixin-20 (PNX-20)]	0.59	0.022
Q9NX62	*BPNT2*	Golgi-resident adenosine 3',5'-bisphosphate 3'-phosphatase (Golgi-resident PAP phosphatase) (gPAPP) (EC 3.1.3.7) (3'(2'), 5'-bisphosphate nucleotidase 2) (Inositol monophosphatase domain-containing protein 1) (Myo-inositol monophosphatase A3) (Phosphoadenosine phosphate 3'-nucleotidase)	0.55	0.022
P04350	*TUBB4A*	Tubulin beta-4A chain (Tubulin 5 beta) (Tubulin beta-4 chain)	0.69	0.023
A0A024R9G4	*FAM49B*	Family with sequence similarity 49, member B, isoform CRA_a	0.63	0.023

P62861	*FAU*	40S ribosomal protein S30 (Small ribosomal subunit protein eS30)	2.70	0.023
P48739	*PITPNB*	Phosphatidylinositol transfer protein beta isoform (PI-TP-beta) (PtdIns transfer protein beta) (PtdInsTP beta)	0.66	0.024
P0DMN0	*SULT1A4*	Sulfotransferase 1A4 (ST1A4) (EC 2.8.2.1) (Aryl sulfotransferase 1A3/1A4) (Sulfotransferase 1A3/1A4)	0.65	0.024
Q9BW92	*TARS2*	Threonine--tRNA ligase, mitochondrial (EC 6.1.1.3) (Threonyl-tRNA synthetase) (ThrRS) (Threonyl-tRNA synthetase-like 1)	0.54	0.025
Q9UPQ0	*LIMCH1*	LIM and calponin homology domains-containing protein 1	1.31	0.025
Q9UBF2	*COPG2*	Coatomer subunit gamma-2 (Gamma-2-coat protein) (Gamma-2-COP)	0.66	0.025
Q99766	*DMAC2L*	ATP synthase subunit s, mitochondrial (ATP synthase-coupling factor B) (FB) (Distal membrane arm assembly complex 2-like protein) (Mitochondrial ATP synthase regulatory component factor B)	0.67	0.026
Q9Y608	*LRRFIP2*	Leucine-rich repeat flightless-interacting protein 2 (LRR FLII-interacting protein 2)	1.40	0.026
Q8NDI1	*EHBP1*	EH domain-binding protein 1	0.74	0.027
Q58WW2	*DCAF6*	DDB1- and CUL4-associated factor 6 (Androgen receptor complex-associated protein) (ARCAP) (IQ motif and WD repeat-containing protein 1) (Nuclear receptor interaction protein) (NRIP)	1.55	0.028
Q99541	*PLIN2*	Perilipin-2 (Adipophilin) (Adipose differentiation-related protein) (ADRP)	1.82	0.028
Q96AQ8	*MCUR1*	Mitochondrial calcium uniporter regulator 1 (MCU regulator 1) (Coiled-coil domain-containing protein 90A, mitochondrial)	0.66	0.028
P50897	*PPT1*	Palmitoyl-protein thioesterase 1 (PPT-1) (EC 3.1.2.22) (Palmitoyl-protein hydrolase 1)	0.55	0.028
P13796	*LCP1*	Plastin-2 (L-plastin) (LC64P) (Lymphocyte cytosolic protein 1) (LCP-1)	0.58	0.028
P48729	*CSNK1A1*	Casein kinase I isoform alpha (CKI-alpha) (EC 2.7.11.1) (CK1)	0.64	0.028
O95376	*ARIH2*	E3 ubiquitin-protein ligase ARIH2 (ARI-2) (Protein ariadne-2 homolog) (EC 2.3.2.31) (RING-type E3 ubiquitin transferase ARIH2) (Triad1 protein)	0.60	0.029
Q9P015	*MRPL15*	39S ribosomal protein L15, mitochondrial (L15mt) (MRP-L15) (Mitochondrial large ribosomal subunit protein uL15m)	1.69	0.029
B4DRE5		cDNA FLJ55391	0.48	0.029

P05164	*MPO*	Myeloperoxidase (MPO) (EC 1.11.2.2) [Cleaved into: Myeloperoxidase; 89 kDa myeloperoxidase; 84 kDa myeloperoxidase; Myeloperoxidase light chain; Myeloperoxidase heavy chain]	0.41	0.029
Q06124	*PTPN11*	Tyrosine-protein phosphatase non-receptor type 11 (EC 3.1.3.48) (Protein-tyrosine phosphatase 1D) (PTP-1D) (Protein-tyrosine phosphatase 2C) (PTP-2C) (SH-PTP2) (SHP-2) (Shp2) (SH-PTP3)	0.76	0.030
Q5HYK3	*COQ5*	2-methoxy-6-polyprenyl-1,4-benzoquinol methylase, mitochondrial (EC 2.1.1.201) (Ubiquinone biosynthesis methyltransferase COQ5)	0.70	0.030
Q7L3T8	*PARS2*	Probable proline--tRNA ligase, mitochondrial (EC 6.1.1.15) (Prolyl-tRNA synthetase) (ProRS)	0.72	0.030
Q53FA7	*TP53I3*	Quinone oxidoreductase PIG3 (EC 1.-.-.-) (Tumor protein p53-inducible protein 3) (p53-induced gene 3 protein)	0.57	0.030
O60237	*PPP1R12B*	Protein phosphatase 1 regulatory subunit 12B (Myosin phosphatase-targeting subunit 2) (Myosin phosphatase target subunit 2)	0.72	0.032
Q9Y3C8	*UFC1*	Ubiquitin-fold modifier-conjugating enzyme 1 (Ufm1-conjugating enzyme 1)	0.66	0.032
V9HW41	*HEL-S-71*	Epididymis secretory protein Li 71 (Ubiquitin-conjugating enzyme E2N (UBC13 homolog, yeast), isoform CRA_a)	0.76	0.032
V9HW01	*HEL-S-310*	Epididymis secretory protein Li 310	1.63	0.032
Q13131	*PRKAA1*	5'-AMP-activated protein kinase catalytic subunit alpha-1 (AMPK subunit alpha-1) (EC 2.7.11.1) (Acetyl-CoA carboxylase kinase) (ACACA kinase) (EC 2.7.11.27) (Hydroxymethylglutaryl-CoA reductase kinase) (HMGCR kinase) (EC 2.7.11.31) (Tau-protein kinase PRKAA1) (EC 2.7.11.26)	0.75	0.033
O75396	*SEC22B*	Vesicle-trafficking protein SEC22b (ER-Golgi SNARE of 24 kDa) (ERS-24) (ERS24) (SEC22 vesicle-trafficking protein homolog B) (SEC22 vesicle-trafficking protein-like 1)	0.69	0.033
P54289	*CACNA2D1*	Voltage-dependent calcium channel subunit alpha-2/delta-1 (Voltage-gated calcium channel subunit alpha-2/delta-1) [Cleaved into: Voltage-dependent calcium channel subunit alpha-2-1; Voltage-dependent calcium channel subunit delta-1]	0.73	0.033
U3KX66	*ATP8*	ATP synthase protein 8	1.50	0.033
Q14896	*MYBPC3*	Myosin-binding protein C, cardiac-type (Cardiac MyBP-C) (C-protein, cardiac muscle isoform)	1.97	0.034

P27361	*MAPK3*	Mitogen-activated protein kinase 3 (MAP kinase 3) (MAPK 3) (EC 2.7.11.24) (ERT2) (Extracellular signal-regulated kinase 1) (ERK-1) (Insulin-stimulated MAP2 kinase) (MAP kinase isoform p44) (p44-MAPK) (Microtubule-associated protein 2 kinase) (p44-ERK1)	0.69	0.034
P68400	*CSNK2A1*	Casein kinase II subunit alpha (CK II alpha) (EC 2.7.11.1)	0.69	0.034
Q9BUT1	*BDH2*	3-hydroxybutyrate dehydrogenase type 2 (EC 1.1.1.-) (EC 1.1.1.30) (Dehydrogenase/reductase SDR family member 6) (Oxidoreductase UCPA) (R-beta-hydroxybutyrate dehydrogenase) (Short chain dehydrogenase/reductase family 15C member 1)	0.62	0.034
P28906	*CD34*	Hematopoietic progenitor cell antigen CD34 (CD antigen CD34)	0.68	0.034
Q5BKX8	*CAVIN4*	Caveolae-associated protein 4 (Muscle-related coiled-coil protein) (Muscle-restricted coiled-coil protein)	0.66	0.035
Q00765	*REEP5*	Receptor expression-enhancing protein 5 (Polyposis locus protein 1) (Protein TB2)	0.71	0.035
Q567U6	*CCDC93*	Coiled-coil domain-containing protein 93	0.67	0.035
Q14520	*HABP2*	Hyaluronan-binding protein 2 (EC 3.4.21.-) (Factor VII-activating protease) (Factor seven-activating protease) (FSAP) (Hepatocyte growth factor activator-like protein) (Plasma hyaluronan-binding protein) [Cleaved into: Hyaluronan-binding protein 2 50 kDa heavy chain; Hyaluronan-binding protein 2 50 kDa heavy chain alternate form; Hyaluronan-binding protein 2 27 kDa light chain; Hyaluronan-binding protein 2 27 kDa light chain alternate form]	2.05	0.035
B4DR48		Arginyl-tRNA--protein transferase 1 (Arginyltransferase 1) (R-transferase 1) (EC 2.3.2.8) (Arginine-tRNA--protein transferase 1)	1.72	0.036
Q92504	*SLC39A7*	Zinc transporter SLC39A7 (Histidine-rich membrane protein Ke4) (Really interesting new gene 5 protein) (Solute carrier family 39 member 7) (Zrt-, Irt-like protein 7) (ZIP7)	0.71	0.037
E7ERI8	*CLASP2*	CLIP-associating protein 2	0.65	0.037
Q6FIC5	*CLIC4*	Chloride intracellular channel protein	1.60	0.037
Q92688	*ANP32B*	Acidic leucine-rich nuclear phosphoprotein 32 family member B (Acidic protein rich in leucines) (Putative HLA-DR-associated protein I-2) (PHAPI2) (Silver-stainable protein SSP29)	0.60	0.037
Q969V3	*NCLN*	Nicalin (Nicastrin-like protein)	1.78	0.038

P52948	*NUP98*	Nuclear pore complex protein Nup98-Nup96 (EC 3.4.21.-) [Cleaved into: Nuclear pore complex protein Nup98 (98 kDa nucleoporin) (Nucleoporin Nup98) (Nup98); Nuclear pore complex protein Nup96 (96 kDa nucleoporin) (Nucleoporin Nup96) (Nup96)]	0.77	0.038
P46379	*BAG6*	Large proline-rich protein BAG6 (BAG family molecular chaperone regulator 6) (BCL2-associated athanogene 6) (BAG-6) (HLA-B-associated transcript 3) (Protein G3) (Protein Scythe)	1.62	0.038
W8QEY1		Lactotransferrin	0.48	0.038
Q9H3U1	*UNC45A*	Protein unc-45 homolog A (Unc-45A) (GCUNC-45) (Smooth muscle cell-associated protein 1) (SMAP-1)	0.44	0.038
P11169	*SLC2A3*	Solute carrier family 2, facilitated glucose transporter member 3 (Glucose transporter type 3, brain) (GLUT-3)	0.12	0.039
P13647	*KRT5*	Keratin, type II cytoskeletal 5 (58 kDa cytokeratin) (Cytokeratin-5) (CK-5) (Keratin-5) (K5) (Type-II keratin Kb5)	1.85	0.040
O60506	*SYNCRIP*	Heterogeneous nuclear ribonucleoprotein Q (hnRNP Q) (Glycine- and tyrosine-rich RNA-binding protein) (GRY-RBP) (NS1-associated protein 1) (Synaptotagmin-binding, cytoplasmic RNA-interacting protein)	0.59	0.040
P61011	*SRP54*	Signal recognition particle 54 kDa protein (SRP54) (EC 3.6.5.-)	0.61	0.040
Q9H910	*JPT2*	Jupiter microtubule associated homolog 2 (Hematological and neurological expressed 1-like protein) (HN1-like protein)	0.68	0.040
P25705	*ATP5F1A*	ATP synthase subunit alpha, mitochondrial (ATP synthase F1 subunit alpha)	0.76	0.040
V9HVY3	*HEL-S-269*	Protein disulfide-isomerase (EC 5.3.4.1)	0.73	0.042
Q9Y3P9	*RABGAP1*	Rab GTPase-activating protein 1 (GAP and centrosome-associated protein) (Rab6 GTPase-activating protein GAPCenA)	0.63	0.042
A6N930	*MYH7*	Mutant cardiac beta myosin heavy chain (Fragment)	1.59	0.042
Q96I23	*PYURF*	Protein preY, mitochondrial (PIGY upstream reading frame protein)	0.75	0.042
O15247	*CLIC2*	Chloride intracellular channel protein 2 (XAP121)	1.84	0.043
Q8IUX1	*TMEM126B*	Complex I assembly factor TMEM126B, mitochondrial (Transmembrane protein 126B)	0.68	0.044
P12829	*MYL4*	Myosin light chain 4 (Myosin light chain 1, embryonic muscle/atrial isoform) (Myosin light chain alkali GT-1 isoform)	3.32	0.046

A0A024QZM6	*SEC24C*	SEC24 related gene family, member C (S. cerevisiae), isoform CRA_a	1.57	0.046
B4E106		Monocarboxylate transporter 1 (Solute carrier family 16 member 1)	0.76	0.046
P62072	*TIMM10*	Mitochondrial import inner membrane translocase subunit Tim10	0.74	0.047
P12821	*ACE*	Angiotensin-converting enzyme (ACE) (EC 3.2.1.-) (EC 3.4.15.1) (Dipeptidyl carboxypeptidase I) (Kininase II) (CD antigen CD143) [Cleaved into: Angiotensin-converting enzyme, soluble form]	1.95	0.047
Q9NZJ9	*NUDT4*	Diphosphoinositol polyphosphate phosphohydrolase 2 (DIPP-2) (EC 3.6.1.52) (Diadenosine 5',5'''-P1,P6-hexaphosphate hydrolase 2) (Nucleoside diphosphate-linked moiety X motif 4) (Nudix motif 4)	2.06	0.048
P19838	*NFKB1*	Nuclear factor NF-kappa-B p105 subunit (DNA-binding factor KBF1) (EBP-1) (Nuclear factor of kappa light polypeptide gene enhancer in B-cells 1) [Cleaved into: Nuclear factor NF-kappa-B p50 subunit]	1.65	0.048
Q9UM22	*EPDR1*	Mammalian ependymin-related protein 1 (MERP-1) (Upregulated in colorectal cancer gene 1 protein)	1.56	0.049
P61254	*RPL26*	60S ribosomal protein L26 (Large ribosomal subunit protein uL24)	1.46	0.049
P49721	*PSMB2*	Proteasome subunit beta type-2 (EC 3.4.25.1) (Macropain subunit C7-I) (Multicatalytic endopeptidase complex subunit C7-I) (Proteasome component C7-I)	3.78	0.050
Proteins differentially (fold change >1.3 or <0.769) and significantly (P<0.05) expressed in AVS vs. CAD post-reperfusion LV samples. AVS, aortic valve stenosis; CAD, coronary artery disease; LV, light ventricle.				

Table SXV. Differentially expressed proteins from RV post-reperfusion samples between AVS and CAD patients.				
Accession no.	Gene name	Description	Fold change	p-value
Q14896	*MYBPC3*	Myosin-binding protein C, cardiac-type (Cardiac MyBP-C) (C-protein, cardiac muscle isoform)	3.76	0.000
B7Z6P1		cDNA FLJ53662, highly similar to Actin, alpha skeletal muscle	3.08	0.000
Q86Y39	*NDUFA11*	NADH dehydrogenase [ubiquinone] 1 alpha subcomplex subunit 11 (Complex I-B14.7) (CI-B14.7) (NADH-ubiquinone oxidoreductase subunit B14.7)	0.59	0.001
P12883	*MYH7*	Myosin-7 (Myosin heavy chain 7) (Myosin heavy chain slow isoform) (MyHC-slow) (Myosin heavy chain, cardiac muscle beta isoform) (MyHC-beta)	4.33	0.001
P0C0L5	*C4B; C4B_2*	Complement C4-B (Basic complement C4) (C3 and PZP-like alpha-2-macroglobulin domain-containing protein 3) [Cleaved into: Complement C4 beta chain; Complement C4-B alpha chain; C4a anaphylatoxin; C4b-B; C4d-B; Complement C4 gamma chain]	0.33	0.001
P15954	*COX7C*	Cytochrome c oxidase subunit 7C, mitochondrial (Cytochrome c oxidase polypeptide VIIc)	0.50	0.001
P02794	*FTH1*	Ferritin heavy chain (Ferritin H subunit) (EC 1.16.3.1) (Cell proliferation-inducing gene 15 protein) [Cleaved into: Ferritin heavy chain, N-terminally processed]	3.00	0.001
A0A024R2Q5	*MYL3*	Myosin, light polypeptide 3, alkali ventricular, skeletal, slow, isoform CRA_a	3.21	0.001
P55039	*DRG2*	Developmentally-regulated GTP-binding protein 2 (DRG-2) (Translation factor GTPase DRG2) (TRAFAC GTPase DRG2) (EC 3.6.5.-)	1.36	0.001
P62861	*FAU*	40S ribosomal protein S30 (Small ribosomal subunit protein eS30)	4.51	0.001
P19429	*TNNI3*	Troponin I, cardiac muscle (Cardiac troponin I)	1.73	0.001
Q86TI0	*TBC1D1*	TBC1 domain family member 1	0.23	0.001
P50895	*BCAM*	Basal cell adhesion molecule (Auberger B antigen) (B-CAM cell surface glycoprotein) (F8/G253 antigen) (Lutheran antigen) (Lutheran blood group glycoprotein) (CD antigen CD239)	0.59	0.001
P61626	*LYZ*	Lysozyme C (EC 3.2.1.17) (1,4-beta-N-acetylmuramidase C)	0.26	0.001
P02748	*C9*	Complement component C9 [Cleaved into: Complement component C9a; Complement component C9b]	0.39	0.001

P43307	*SSR1*	Translocon-associated protein subunit alpha (TRAP-alpha) (Signal sequence receptor subunit alpha) (SSR-alpha)	0.60	0.001
P60033	*CD81*	CD81 antigen (26 kDa cell surface protein TAPA-1) (Target of the antiproliferative antibody 1) (Tetraspanin-28) (Tspan-28) (CD antigen CD81)	0.47	0.002
A4D2P0	*RAC1*	Ras-related C3 botulinum toxin substrate 1 (Rho family, small GTP binding protein Rac1) (Ras-related C3 botulinum toxin substrate 1 (Rho family, small GTP binding protein Rac1), isoform CRA_e) (cDNA FLJ77333, highly similar to Homo sapiens ras-related C3 botulinum toxin substrate 1 (rho family, small GTP binding protein Rac1) (RAC1), transcript variant Rac1b, mRNA)	0.70	0.002
Q9UNF1	*MAGED2*	Melanoma-associated antigen D2 (11B6) (Breast cancer-associated gene 1 protein) (BCG-1) (Hepatocellular carcinoma-associated protein JCL-1) (MAGE-D2 antigen)	1.37	0.002
P30626	*SRI*	Sorcin (22 kDa protein) (CP-22) (CP22) (V19)	0.45	0.002
P13533	*MYH6*	Myosin-6 (Myosin heavy chain 6) (Myosin heavy chain, cardiac muscle alpha isoform) (MyHC-alpha)	3.66	0.003
P02786	*TFRC*	Transferrin receptor protein 1 (TR) (TfR) (TfR1) (Trfr) (T9) (p90) (CD antigen CD71) [Cleaved into: Transferrin receptor protein 1, serum form (sTfR)]	0.67	0.003
D3DRP5	*C9orf19*	Chromosome 9 open reading frame 19, isoform CRA_a (Fragment)	0.55	0.003
Q96ER9	*CCDC51*	Mitochondrial potassium channel (MITOK) (Coiled-coil domain-containing protein 51)	0.66	0.004
P12829	*MYL4*	Myosin light chain 4 (Myosin light chain 1, embryonic muscle/atrial isoform) (Myosin light chain alkali GT-1 isoform)	15.89	0.004
B4DUI8		cDNA FLJ52761, highly similar to Actin, aortic smooth muscle	3.24	0.004
P00367	*GLUD1*	Glutamate dehydrogenase 1, mitochondrial (GDH 1) (EC 1.4.1.3)	0.74	0.004
P01031	*C5*	Complement C5 (C3 and PZP-like alpha-2-macroglobulin domain-containing protein 4) [Cleaved into: Complement C5 beta chain; Complement C5 alpha chain; C5a anaphylatoxin; Complement C5 alpha' chain]	0.63	0.004
Q9P0L0	*VAPA*	Vesicle-associated membrane protein-associated protein A (VAMP-A) (VAMP-associated protein A) (VAP-A) (33 kDa VAMP-associated protein) (VAP-33)	0.75	0.004

P10515	*DLAT*	Dihydrolipoyllysine-residue acetyltransferase component of pyruvate dehydrogenase complex, mitochondrial (EC 2.3.1.12) (70 kDa mitochondrial autoantigen of primary biliary cirrhosis) (PBC) (Dihydrolipoamide acetyltransferase component of pyruvate dehydrogenase complex) (M2 antigen complex 70 kDa subunit) (Pyruvate dehydrogenase complex component E2) (PDC-E2) (PDCE2)	1.32	0.004
Q6PKG0	*LARP1*	La-related protein 1 (La ribonucleoprotein domain family member 1)	1.57	0.004
P08574	*CYC1*	Cytochrome c1, heme protein, mitochondrial (EC 7.1.1.8) (Complex III subunit 4) (Complex III subunit IV) (Cytochrome b-c1 complex subunit 4) (Ubiquinol-cytochrome-c reductase complex cytochrome c1 subunit) (Cytochrome c-1)	0.73	0.005
Q16795	*NDUFA9*	NADH dehydrogenase [ubiquinone] 1 alpha subcomplex subunit 9, mitochondrial (Complex I-39kD) (CI-39kD) (NADH-ubiquinone oxidoreductase 39 kDa subunit)	0.66	0.005
P00734	*F2*	Prothrombin (EC 3.4.21.5) (Coagulation factor II) [Cleaved into: Activation peptide fragment 1; Activation peptide fragment 2; Thrombin light chain; Thrombin heavy chain]	0.42	0.005
A0A2R8Y6G6	*ENO1*	2-phospho-D-glycerate hydro-lyase (EC 4.2.1.11)	0.76	0.005
A0A5C2GXF4		IG c1927_light_IGKV3-20_IGKJ4 (Fragment)	0.43	0.005
Q7Z4H8	*POGLUT3*	Protein O-glucosyltransferase 3 (EC 2.4.1.-) (KDEL motif-containing protein 2) (Protein O-xylosyltransferase POGLUT3) (EC 2.4.2.-)	0.51	0.005
P07225	*PROS1*	Vitamin K-dependent protein S	0.66	0.005
Q7KYR7	*BTN2A1*	Butyrophilin subfamily 2 member A1	0.50	0.006
O60783	*MRPS14*	28S ribosomal protein S14, mitochondrial (MRP-S14) (S14mt) (Mitochondrial small ribosomal subunit protein uS14m)	0.25	0.006
P08603	*CFH*	Complement factor H (H factor 1)	0.44	0.006
P47756	*CAPZB*	F-actin-capping protein subunit beta (CapZ beta)	1.62	0.006
B1AK88	*CAPZB*	F-actin-capping protein subunit beta	1.55	0.006
Q9UBY9	*HSPB7*	Heat shock protein beta-7 (HspB7) (Cardiovascular heat shock protein) (cvHsp)	1.53	0.006

P52948	*NUP98*	Nuclear pore complex protein Nup98-Nup96 (EC 3.4.21.-) [Cleaved into: Nuclear pore complex protein Nup98 (98 kDa nucleoporin) (Nucleoporin Nup98) (Nup98); Nuclear pore complex protein Nup96 (96 kDa nucleoporin) (Nucleoporin Nup96) (Nup96)]	0.63	0.006
A0A140VK00		Testicular tissue protein Li 227	2.19	0.006
P38159	*RBMX*	RNA-binding motif protein, X chromosome (Glycoprotein p43) (Heterogeneous nuclear ribonucleoprotein G) (hnRNP G) [Cleaved into: RNA-binding motif protein, X chromosome, N-terminally processed]	0.59	0.006
X5DR21	*IQCE*	IQ motif containing E isoform A (Fragment)	0.38	0.007
Q96BW5	*PTER*	Phosphotriesterase-related protein (EC 3.1.-.-) (Parathion hydrolase-related protein) (hPHRP)	0.65	0.007
P98095	*FBLN2*	Fibulin-2 (FIBL-2)	0.45	0.007
O00217	*NDUFS8*	NADH dehydrogenase [ubiquinone] iron-sulfur protein 8, mitochondrial (EC 7.1.1.2) (Complex I-23kD) (CI-23kD) (NADH-ubiquinone oxidoreductase 23 kDa subunit) (TYKY subunit)	0.75	0.007
A0A0S2Z2Z6	*ANXA6*	Annexin (Fragment)	0.62	0.007
O00231	*PSMD11*	26S proteasome non-ATPase regulatory subunit 11 (26S proteasome regulatory subunit RPN6) (26S proteasome regulatory subunit S9) (26S proteasome regulatory subunit p44.5)	0.73	0.007
Q13522	*PPP1R1A*	Protein phosphatase 1 regulatory subunit 1A (Protein phosphatase inhibitor 1) (I-1) (IPP-1)	0.45	0.007
O15327	*INPP4B*	Inositol polyphosphate 4-phosphatase type II (Type II inositol 3,4-bisphosphate 4-phosphatase) (EC 3.1.3.66)	2.22	0.007
Q5JPE7	*NOMO2*	Nodal modulator 2 (pM5 protein 2)	0.50	0.007
A0A5C2GW15		IG c1457_light_IGKV3-20_IGKJ1 (Fragment)	0.60	0.008
Q702N8	*XIRP1*	Xin actin-binding repeat-containing protein 1 (Cardiomyopathy-associated protein 1)	1.91	0.008
A4D1U3	*SSBP1*	Single-stranded DNA-binding protein, mitochondrial	0.60	0.008
Q6QEF8	*CORO6*	Coronin-6 (Coronin-like protein E) (Clipin-E)	1.67	0.008
Q5U058	*GAP43*	Axonal membrane protein GAP-43 (Growth-associated protein 43) (Neuromodulin)	0.34	0.008

Q6Y1H2	*HACD2*	Very-long-chain (3R)-3-hydroxyacyl-CoA dehydratase 2 (EC 4.2.1.134) (3-hydroxyacyl-CoA dehydratase 2) (HACD2) (Protein-tyrosine phosphatase-like member B)	0.37	0.008
P03952	*KLKB1*	Plasma kallikrein (EC 3.4.21.34) (Fletcher factor) (Kininogenin) (Plasma prekallikrein) (PKK) [Cleaved into: Plasma kallikrein heavy chain; Plasma kallikrein light chain]	0.53	0.009
P05164	*MPO*	Myeloperoxidase (MPO) (EC 1.11.2.2) [Cleaved into: Myeloperoxidase; 89 kDa myeloperoxidase; 84 kDa myeloperoxidase; Myeloperoxidase light chain; Myeloperoxidase heavy chain]	0.32	0.009
P04003	*C4BPA*	C4b-binding protein alpha chain (C4bp) (Proline-rich protein) (PRP)	0.29	0.009
Q13442	*PDAP1*	28 kDa heat- and acid-stable phosphoprotein (PDGF-associated protein) (PAP) (PDGFA-associated protein 1) (PAP1)	1.36	0.009
Q5T013	*HYI*	Putative hydroxypyruvate isomerase (EC 5.3.1.22) (Endothelial cell apoptosis protein E-CE1)	4.36	0.009
Q8NBU5	*ATAD1*	ATPase family AAA domain-containing protein 1 (EC 3.6.1.-) (Thorase)	0.65	0.009
F6S8M0	*GNS*	N-acetylglucosamine-6-sulfatase	0.66	0.009
Q6L8Q7	*PDE12*	2',5'-phosphodiesterase 12 (2'-PDE) (2-PDE) (EC 3.1.4.-) (Mitochondrial deadenylase) (EC 3.1.13.4)	1.86	0.009
P23786	*CPT2*	Carnitine O-palmitoyltransferase 2, mitochondrial (EC 2.3.1.21) (Carnitine palmitoyltransferase II) (CPT II)	0.72	0.010
P12429	*ANXA3*	Annexin A3 (35-alpha calcimedin) (Annexin III) (Annexin-3) (Inositol 1,2-cyclic phosphate 2-phosphohydrolase) (Lipocortin III) (Placental anticoagulant protein III) (PAP-III)	0.75	0.010
P13796	*LCP1*	Plastin-2 (L-plastin) (LC64P) (Lymphocyte cytosolic protein 1) (LCP-1)	0.47	0.010
Q9ULA0	*DNPEP*	Aspartyl aminopeptidase (EC 3.4.11.21)	1.65	0.010
Q7L0Y3	*TRMT10C*	tRNA methyltransferase 10 homolog C (HBV pre-S2 trans-regulated protein 2) (Mitochondrial ribonuclease P protein 1) (Mitochondrial RNase P protein 1) (RNA (guanine-9-)-methyltransferase domain-containing protein 1) (Renal carcinoma antigen NY-REN-49) (mRNA methyladenosine-N(1)-methyltransferase) (EC 2.1.1.-) (tRNA (adenine(9)-N(1))-methyltransferase) (EC 2.1.1.218) (tRNA (guanine(9)-N(1))-methyltransferase) (EC 2.1.1.221)	0.66	0.010
A0A2P9DU05	*ROCK2*	Rho-associated protein kinase (EC 2.7.11.1)	1.55	0.010

Q6FGI7	*COX7A1*	COX7A1 protein (Cytochrome c oxidase subunit VIIa polypeptide 1 (Muscle)) (cDNA, FLJ92372, Homo sapiens cytochrome c oxidase subunit VIIa polypeptide 1(muscle) (COX7A1), mRNA)	0.62	0.010
Q86Y82	*STX12*	Syntaxin-12	0.57	0.010
P53367	*ARFIP1*	Arfaptin-1 (ADP-ribosylation factor-interacting protein 1)	0.27	0.011
Q53H26		Beta-1 metal-binding globulin (Serotransferrin) (Siderophilin) (Fragment)	0.37	0.011
O75298	*RTN2*	Reticulon-2 (Neuroendocrine-specific protein-like 1) (NSP-like protein 1) (Neuroendocrine-specific protein-like I) (NSP-like protein I) (NSPLI)	0.60	0.011
P28906	*CD34*	Hematopoietic progenitor cell antigen CD34 (CD antigen CD34)	0.58	0.011
A0A5C2GXS3		IG c510_light_IGKV3-11_IGKJ5 (Fragment)	0.51	0.011
P04083	*ANXA1*	Annexin A1 (Annexin I) (Annexin-1) (Calpactin II) (Calpactin-2) (Chromobindin-9) (Lipocortin I) (Phospholipase A2 inhibitory protein) (p35)	0.62	0.011
P0DMN0	*SULT1A4*	Sulfotransferase 1A4 (ST1A4) (EC 2.8.2.1) (Aryl sulfotransferase 1A3/1A4) (Sulfotransferase 1A3/1A4)	0.63	0.012
H0YK48	*TPM1*	Tropomyosin alpha-1 chain	2.11	0.012
B7SBB1	*AIP*	Peptidylprolyl isomerase (EC 5.2.1.8)	1.44	0.012
V9HW01	*HEL-S-310*	Epididymis secretory protein Li 310	1.44	0.012
P60953	*CDC42*	Cell division control protein 42 homolog (EC 3.6.5.2) (G25K GTP-binding protein)	0.73	0.012
Q9H2G2	*SLK*	STE20-like serine/threonine-protein kinase (STE20-like kinase) (hSLK) (EC 2.7.11.1) (CTCL tumor antigen se20-9) (STE20-related serine/threonine-protein kinase) (STE20-related kinase) (Serine/threonine-protein kinase 2)	1.50	0.012
A0A0A6YYH1	*C15orf38-AP3S2*	Arpin	0.69	0.012
P49454	*CENPF*	Centromere protein F (CENP-F) (AH antigen) (Kinetochore protein CENPF) (Mitosin)	5.18	0.012
Q6FGH9	*DNCL1*	Dynein light chain	0.41	0.012
O95881	*TXNDC12*	Thioredoxin domain-containing protein 12 (EC 1.8.4.2) (Endoplasmic reticulum resident protein 18) (ER protein 18) (ERp18) (Endoplasmic reticulum resident protein 19) (ER protein 19) (ERp19) (Thioredoxin-like protein p19) (hTLP19)	0.55	0.013

O75891	*ALDH1L1*	Cytosolic 10-formyltetrahydrofolate dehydrogenase (10-FTHFDH) (FDH) (EC 1.5.1.6) (Aldehyde dehydrogenase family 1 member L1)	1.85	0.013
Q8NBS9	*TXNDC5*	Thioredoxin domain-containing protein 5 (Endoplasmic reticulum resident protein 46) (ER protein 46) (ERp46) (Thioredoxin-like protein p46)	0.71	0.013
O60669	*SLC16A7*	Monocarboxylate transporter 2 (MCT 2) (Solute carrier family 16 member 7)	0.68	0.013
Q9H300	*PARL*	Presenilins-associated rhomboid-like protein, mitochondrial (EC 3.4.21.105) (Mitochondrial intramembrane cleaving protease PARL) [Cleaved into: P-beta (Pbeta)]	0.70	0.013
P05026	*ATP1B1*	Sodium/potassium-transporting ATPase subunit beta-1 (Sodium/potassium-dependent ATPase subunit beta-1)	0.54	0.014
A0A384MEF1		Actin-depolymerizing factor (Brevin) (Gelsolin)	2.01	0.014
O00764	*PDXK*	Pyridoxal kinase (EC 2.7.1.35) (Pyridoxine kinase)	1.40	0.014
P50995	*ANXA11*	Annexin A11 (56 kDa autoantigen) (Annexin XI) (Annexin-11) (Calcyclin-associated annexin 50) (CAP-50)	0.69	0.014
Q9HAV0	*GNB4*	Guanine nucleotide-binding protein subunit beta-4 (Transducin beta chain 4)	0.47	0.014
P45379	*TNNT2*	Troponin T, cardiac muscle (TnTc) (Cardiac muscle troponin T) (cTnT)	1.63	0.014
P08648	*ITGA5*	Integrin alpha-5 (CD49 antigen-like family member E) (Fibronectin receptor subunit alpha) (Integrin alpha-F) (VLA-5) (CD antigen CD49e) [Cleaved into: Integrin alpha-5 heavy chain; Integrin alpha-5 light chain]	0.58	0.014
A0A0S2Z3G3	*SLC25A10*	Solute carrier family 25 (Mitochondrial carrier dicarboxylate transporter), member 10, isoform CRA_a (Solute carrier family 25 member 10 isoform 1) (Fragment)	0.74	0.015
P19367	*HK1*	Hexokinase-1 (EC 2.7.1.1) (Brain form hexokinase) (Hexokinase type I) (HK I) (Hexokinase-A)	0.75	0.015
P27361	*MAPK3*	Mitogen-activated protein kinase 3 (MAP kinase 3) (MAPK 3) (EC 2.7.11.24) (ERT2) (Extracellular signal-regulated kinase 1) (ERK-1) (Insulin-stimulated MAP2 kinase) (MAP kinase isoform p44) (p44-MAPK) (Microtubule-associated protein 2 kinase) (p44-ERK1)	0.72	0.015
Q9UMS6	*SYNPO2*	Synaptopodin-2 (Genethonin-2) (Myopodin)	0.69	0.015

P17612	*PRKACA*	cAMP-dependent protein kinase catalytic subunit alpha (PKA C-alpha) (EC 2.7.11.11)	1.47	0.015
O95139	*NDUFB6*	NADH dehydrogenase [ubiquinone] 1 beta subcomplex subunit 6 (Complex I-B17) (CI-B17) (NADH-ubiquinone oxidoreductase B17 subunit)	0.63	0.015
Q9BYT8	*NLN*	Neurolysin, mitochondrial (EC 3.4.24.16) (Angiotensin-binding protein) (Microsomal endopeptidase) (MEP) (Mitochondrial oligopeptidase M) (Neurotensin endopeptidase)	0.50	0.015
Q96PE7	*MCEE*	Methylmalonyl-CoA epimerase, mitochondrial (EC 5.1.99.1) (DL-methylmalonyl-CoA racemase)	0.67	0.016
Q9UGM5	*FETUB*	Fetuin-B (16G2) (Fetuin-like protein IRL685) (Gugu)	0.18	0.016
P00736	*C1R*	Complement C1r subcomponent (EC 3.4.21.41) (Complement component 1 subcomponent r) [Cleaved into: Complement C1r subcomponent heavy chain; Complement C1r subcomponent light chain]	0.44	0.016
P13797	*PLS3*	Plastin-3 (T-plastin)	0.61	0.017
P49903	*SEPHS1*	Selenide, water dikinase 1 (EC 2.7.9.3) (Selenium donor protein 1) (Selenophosphate synthase 1)	0.73	0.017
Q02978	*SLC25A11*	Mitochondrial 2-oxoglutarate/malate carrier protein (OGCP) (Solute carrier family 25 member 11)	1.49	0.017
Q14011	*CIRBP*	Cold-inducible RNA-binding protein (A18 hnRNP) (Glycine-rich RNA-binding protein CIRP)	0.45	0.017
Q8NC56	*LEMD2*	LEM domain-containing protein 2 (hLEM2)	0.68	0.018
B4E1Z4		C3/C5 convertase (EC 3.4.21.47) (Complement factor B) (Complement factor B Ba fragment) (Complement factor B Bb fragment)	0.49	0.018
Q92890	*UFD1*	Ubiquitin recognition factor in ER-associated degradation protein 1 (Ubiquitin fusion degradation protein 1) (UB fusion protein 1)	1.51	0.018
Q5TCU3	*TPM2*	Tropomyosin beta chain	2.03	0.018
P08727	*KRT19*	Keratin, type I cytoskeletal 19 (Cytokeratin-19) (CK-19) (Keratin-19) (K19)	0.30	0.018
Q13685	*AAMP*	Angio-associated migratory cell protein	1.94	0.019
Q9P0J0	*NDUFA13*	NADH dehydrogenase [ubiquinone] 1 alpha subcomplex subunit 13 (Cell death regulatory protein GRIM-19) (Complex I-B16.6) (CI-B16.6) (Gene associated with retinoic and interferon-induced mortality 19 protein) (GRIM-19) (Gene associated with retinoic and IFN-induced mortality 19 protein) (NADH-ubiquinone oxidoreductase B16.6 subunit)	0.67	0.019

Q9UJU6	*DBNL*	Drebrin-like protein (Cervical SH3P7) (Cervical mucin-associated protein) (Drebrin-F) (HPK1-interacting protein of 55 kDa) (HIP-55) (SH3 domain-containing protein 7)	0.59	0.019
Q2M2I8	*AAK1*	AP2-associated protein kinase 1 (EC 2.7.11.1) (Adaptor-associated kinase 1)	1.71	0.019
P02776	*PF4*	Platelet factor 4 (PF-4) (C-X-C motif chemokine 4) (Iroplact) (Oncostatin-A) [Cleaved into: Platelet factor 4, short form (Endothelial cell growth inhibitor)]	0.09	0.020
Q02952	*AKAP12*	A-kinase anchor protein 12 (AKAP-12) (A-kinase anchor protein 250 kDa) (AKAP 250) (Gravin) (Myasthenia gravis autoantigen)	0.74	0.020
Q14894	*CRYM*	Ketimine reductase mu-crystallin (EC 1.5.1.25) (NADP-regulated thyroid-hormone-binding protein)	3.14	0.020
P30533	*LRPAP1*	Alpha-2-macroglobulin receptor-associated protein (Alpha-2-MRAP) (Low density lipoprotein receptor-related protein-associated protein 1) (RAP)	1.34	0.020
A0A024R9B7	*COX6C*	Cytochrome c oxidase polypeptide VIc (Cytochrome c oxidase subunit 6C)	0.60	0.020
P23368	*ME2*	NAD-dependent malic enzyme, mitochondrial (NAD-ME) (EC 1.1.1.38) (Malic enzyme 2)	0.57	0.020
A0A024R5X7	*CLPX*	ClpX caseinolytic peptidase X homolog (E. coli), isoform CRA_a	0.60	0.021
Q7Z460	*CLASP1*	CLIP-associating protein 1 (Cytoplasmic linker-associated protein 1) (Multiple asters homolog 1) (Protein Orbit homolog 1) (hOrbit1)	1.95	0.021
P04899	*GNAI2*	Guanine nucleotide-binding protein G(i) subunit alpha-2 (Adenylate cyclase-inhibiting G alpha protein)	0.70	0.021
P27144	*AK4*	Adenylate kinase 4, mitochondrial (AK 4) (EC 2.7.4.10) (EC 2.7.4.6) (Adenylate kinase 3-like) (GTP:AMP phosphotransferase AK4)	0.73	0.021
Q5TC12	*ATPAF1*	ATP synthase mitochondrial F1 complex assembly factor 1 (ATP11 homolog)	1.60	0.021
B4DW52		cDNA FLJ55253, highly similar to Actin, cytoplasmic 1	2.15	0.021
Q9BVC6	*TMEM109*	Transmembrane protein 109 (Mitsugumin-23) (Mg23)	0.63	0.021
Q6IN99	*IGL@*	IGL@ protein	0.51	0.021
Q8N0X4	*CLYBL*	Citramalyl-CoA lyase, mitochondrial (EC 4.1.3.25) ((3S)-malyl-CoA thioesterase) (EC 3.1.2.30) (Beta-methylmalate synthase) (EC 2.3.3.-) (Citrate lyase subunit beta-like protein) (Citrate lyase beta-like) (Malate synthase) (EC 2.3.3.9)	0.42	0.021

Q9BW92	*TARS2*	Threonine--tRNA ligase, mitochondrial (EC 6.1.1.3) (Threonyl-tRNA synthetase) (ThrRS) (Threonyl-tRNA synthetase-like 1)	0.50	0.021
A0A1B0GV45	*MYO18A*	Unconventional myosin-XVIIIa (Fragment)	3.12	0.021
V9HWI6	*HEL-S-51*	Gc-globulin (Group-specific component) (Vitamin D-binding protein)	0.61	0.021
P39059	*COL15A1*	Collagen alpha-1(XV) chain [Cleaved into: Restin (Endostatin-XV) (Related to endostatin) (Restin-I); Restin-2 (Restin-II); Restin-3 (Restin-III); Restin-4 (Restin-IV)]	0.50	0.021
Q13217	*DNAJC3*	DnaJ homolog subfamily C member 3 (Endoplasmic reticulum DNA J domain-containing protein 6) (ER-resident protein ERdj6) (ERdj6) (Interferon-induced, double-stranded RNA-activated protein kinase inhibitor) (Protein kinase inhibitor of 58 kDa) (Protein kinase inhibitor p58)	0.66	0.022
Q9P0P8	*MTRES1*	Mitochondrial transcription rescue factor 1	0.33	0.022
P62263	*RPS14*	40S ribosomal protein S14 (Small ribosomal subunit protein uS11)	1.81	0.022
A0A494C1K3	*GTF2I*	General transcription factor II-I	1.47	0.022
A0A384NL93		Cytochrome c oxidase polypeptide Vb (Cytochrome c oxidase subunit 5B, mitochondrial)	0.76	0.022
A0A5C2GXC2		IG c1247_heavy_IGHV1-69_IGHD1-1_IGHJ6 (Fragment)	0.49	0.022
A0A5C2GIP7		IG c88_light_IGLV3-25_IGLJ2 (Fragment)	0.52	0.022
Q8TE73	*DNAH5*	Dynein heavy chain 5, axonemal (Axonemal beta dynein heavy chain 5) (Ciliary dynein heavy chain 5)	9.64	0.023
Q9BYV7	*BCO2*	Beta,beta-carotene 9',10'-oxygenase (EC 1.13.11.71) (B-diox-II) (Beta-carotene dioxygenase 2)	0.49	0.023
P09871	*C1S*	Complement C1s subcomponent (EC 3.4.21.42) (C1 esterase) (Complement component 1 subcomponent s) [Cleaved into: Complement C1s subcomponent heavy chain; Complement C1s subcomponent light chain]	0.59	0.023
B3KMX3		cDNA FLJ12857 fis, clone NT2RP2003513, highly similar to Homo sapiens paralemmin (PALM), transcript variant 2, mRNA	0.40	0.024
Q9BQ95	*ECSIT*	Evolutionarily conserved signaling intermediate in Toll pathway, mitochondrial (Protein SITPEC)	0.65	0.024
A0A024R1N1	*MYH9*	Myosin, heavy polypeptide 9, non-muscle, isoform CRA_a	1.44	0.024

P16070	*CD44*	CD44 antigen (CDw44) (Epican) (Extracellular matrix receptor III) (ECMR-III) (GP90 lymphocyte homing/adhesion receptor) (HUTCH-I) (Heparan sulfate proteoglycan) (Hermes antigen) (Hyaluronate receptor) (Phagocytic glycoprotein 1) (PGP-1) (Phagocytic glycoprotein I) (PGP-I) (CD antigen CD44)	0.43	0.024
Q5QPK2	*DPM1*	Dolichol-phosphate mannosyltransferase subunit 1 (EC 2.4.1.83)	0.42	0.025
Q12904	*AIMP1*	Aminoacyl tRNA synthase complex-interacting multifunctional protein 1 (Multisynthase complex auxiliary component p43) [Cleaved into: Endothelial monocyte-activating polypeptide 2 (EMAP-2) (Endothelial monocyte-activating polypeptide II) (EMAP-II) (Small inducible cytokine subfamily E member 1)]	0.70	0.025
Q9UFG5	*C19orf25*	UPF0449 protein C19orf25	2.16	0.025
Q6ZN40	*TPM1*	Tropomyosin 1 (Alpha), isoform CRA_f (Tropomyosin alpha-1 chain) (cDNA FLJ16459 fis, clone BRCAN2002473, moderately similar to Tropomyosin, fibroblast isoform 2)	1.55	0.025
P05023	*ATP1A1*	Sodium/potassium-transporting ATPase subunit alpha-1 (Na(+)/K(+) ATPase alpha-1 subunit) (EC 7.2.2.13) (Sodium pump subunit alpha-1)	0.68	0.026
Q9BW91	*NUDT9*	ADP-ribose pyrophosphatase, mitochondrial (EC 3.6.1.13) (ADP-ribose diphosphatase) (ADP-ribose phosphohydrolase) (Adenosine diphosphoribose pyrophosphatase) (ADPR-PPase) (Nucleoside diphosphate-linked moiety X motif 9) (Nudix motif 9)	0.70	0.026
P54886	*ALDH18A1*	Delta-1-pyrroline-5-carboxylate synthase (P5CS) (Aldehyde dehydrogenase family 18 member A1) [Includes: Glutamate 5-kinase (GK) (EC 2.7.2.11) (Gamma-glutamyl kinase); Gamma-glutamyl phosphate reductase (GPR) (EC 1.2.1.41) (Glutamate-5-semialdehyde dehydrogenase) (Glutamyl-gamma-semialdehyde dehydrogenase)]	0.18	0.026
C9JWC3	*SORBS2*	Sorbin and SH3 domain-containing protein 2 (Fragment)	1.45	0.026
O95819	*MAP4K4*	Mitogen-activated protein kinase kinase kinase kinase 4 (EC 2.7.11.1) (HPK/GCK-like kinase HGK) (MAPK/ERK kinase kinase kinase 4) (MEK kinase kinase 4) (MEKKK 4) (Nck-interacting kinase)	1.77	0.026
P37108	*SRP14*	Signal recognition particle 14 kDa protein (SRP14) (18 kDa Alu RNA-binding protein)	0.58	0.026

P24158	*PRTN3*	Myeloblastin (EC 3.4.21.76) (AGP7) (C-ANCA antigen) (Leukocyte proteinase 3) (PR-3) (PR3) (Neutrophil proteinase 4) (NP-4) (P29) (Wegener autoantigen)	0.17	0.026
B2R4R0	*HIST1H4J*	Histone H4	0.39	0.027
P62851	*RPS25*	40S ribosomal protein S25 (Small ribosomal subunit protein eS25)	0.55	0.027
P49821	*NDUFV1*	NADH dehydrogenase [ubiquinone] flavoprotein 1, mitochondrial (EC 7.1.1.2) (Complex I-51kD) (CI-51kD) (NADH dehydrogenase flavoprotein 1) (NADH-ubiquinone oxidoreductase 51 kDa subunit)	0.61	0.027
Q58WW2	*DCAF6*	DDB1- and CUL4-associated factor 6 (Androgen receptor complex-associated protein) (ARCAP) (IQ motif and WD repeat-containing protein 1) (Nuclear receptor interaction protein) (NRIP)	1.71	0.027
B7Z4K1		cDNA FLJ50104, highly similar to Alpha-actinin-2	0.27	0.027
Q96L96	*ALPK3*	Alpha-protein kinase 3 (EC 2.7.11.1) (Muscle alpha-protein kinase)	1.46	0.027
Q8IXI2	*RHOT1*	Mitochondrial Rho GTPase 1 (MIRO-1) (hMiro-1) (EC 3.6.5.-) (Rac-GTP-binding protein-like protein) (Ras homolog gene family member T1)	0.77	0.028
Q96PC5	*MIA2*	Melanoma inhibitory activity protein 2 (MIA protein 2) (CTAGE family member 5 ER export factor) (Cutaneous T-cell lymphoma-associated antigen 5) (Meningioma-expressed antigen 6/11)	0.56	0.028
B7Z539		cDNA FLJ56954, highly similar to Inter-alpha-trypsin inhibitor heavy chain H1	0.57	0.028
Q15056	*EIF4H*	Eukaryotic translation initiation factor 4H (eIF-4H) (Williams-Beuren syndrome chromosomal region 1 protein)	0.58	0.028
P11233	*RALA*	Ras-related protein Ral-A (EC 3.6.5.2)	0.67	0.028
P02760	*AMBP*	Protein AMBP [Cleaved into: Alpha-1-microglobulin (Protein HC) (Alpha-1 microglycoprotein) (Complex-forming glycoprotein heterogeneous in charge); Inter-alpha-trypsin inhibitor light chain (ITI-LC) (Bikunin) (EDC1) (HI-30) (Uronic-acid-rich protein); Trypstatin]	0.38	0.028
P0DOX7		Immunoglobulin kappa light chain (Immunoglobulin kappa light chain EU)	0.43	0.028
A0A140VJG8		Catechol O-methyltransferase (EC 2.1.1.6)	1.75	0.028
A0A024R7V6	*RAB2*	RAB2, member RAS oncogene family, isoform CRA_a	1.36	0.028
Q53FE8		NSFL1 cofactor p47 (p97 cofactor p47) (Fragment)	1.33	0.028

Q13508	*ART3*	Ecto-ADP-ribosyltransferase 3 (EC 2.4.2.31) (ADP-ribosyltransferase C2 and C3 toxin-like 3) (ARTC3) (Mono(ADP-ribosyl)transferase 3) (NAD(P)(+)--arginine ADP-ribosyltransferase 3)	0.48	0.028
A0A3S6RG84	*HLA-C*	MHC class I antigen	0.51	0.029
P08294	*SOD3*	Extracellular superoxide dismutase [Cu-Zn] (EC-SOD) (EC 1.15.1.1)	0.42	0.029
P13010	*XRCC5*	X-ray repair cross-complementing protein 5 (EC 3.6.4.-) (86 kDa subunit of Ku antigen) (ATP-dependent DNA helicase 2 subunit 2) (ATP-dependent DNA helicase II 80 kDa subunit) (CTC box-binding factor 85 kDa subunit) (CTC85) (CTCBF) (DNA repair protein XRCC5) (Ku80) (Ku86) (Lupus Ku autoantigen protein p86) (Nuclear factor IV) (Thyroid-lupus autoantigen) (TLAA) (X-ray repair complementing defective repair in Chinese hamster cells 5 (double-strand-break rejoining))	0.71	0.029
Q9H3U1	*UNC45A*	Protein unc-45 homolog A (Unc-45A) (GCUNC-45) (Smooth muscle cell-associated protein 1) (SMAP-1)	0.38	0.029
Q5R372	*RABGAP1L*	Rab GTPase-activating protein 1-like	1.69	0.029
A0A024R648	*TIMM9*	Translocase of inner mitochondrial membrane 9 homolog (Yeast), isoform CRA_a	0.56	0.029
A0A5C2G0Y6		IGL c2921_light_IGLV1-44_IGLJ2 (Fragment)	0.39	0.029
P59666	*DEFA3*	Neutrophil defensin 3 (Defensin, alpha 3) (HNP-3) (HP-3) (HP3) [Cleaved into: HP 3-56; Neutrophil defensin 2 (HNP-2) (HP-2) (HP2)]	0.17	0.030
S6C4S0		IgG H chain	0.71	0.030
P62750	*RPL23A*	60S ribosomal protein L23a (Large ribosomal subunit protein uL23)	1.45	0.030
Q8IXI1	*RHOT2*	Mitochondrial Rho GTPase 2 (MIRO-2) (hMiro-2) (EC 3.6.5.-) (Ras homolog gene family member T2)	0.73	0.030
Q9HBU9	*POPDC2*	Popeye domain-containing protein 2 (Popeye protein 2)	2.41	0.031
Q9BZV1	*UBXN6*	UBX domain-containing protein 6 (UBX domain-containing protein 1)	1.83	0.031
A0A5C2FX10		IGL c95_light_IGLV4-69_IGLJ2 (Fragment)	0.55	0.031
Q8TEC5	*SH3RF2*	E3 ubiquitin-protein ligase SH3RF2 (EC 2.3.2.27) (Heart protein phosphatase 1-binding protein) (HEPP1) (POSH-eliminating RING protein) (Protein phosphatase 1 regulatory subunit 39) (RING finger	7.44	0.031

		protein 158) (RING-type E3 ubiquitin transferase SH3RF2) (SH3 domain-containing RING finger protein 2)		
P27635	*RPL10*	60S ribosomal protein L10 (Laminin receptor homolog) (Large ribosomal subunit protein uL16) (Protein QM) (Ribosomal protein L10) (Tumor suppressor QM)	0.72	0.031
E9PEW8	*HBD*	Hemoglobin subunit delta (Fragment)	0.22	0.031
P05783	*KRT18*	Keratin, type I cytoskeletal 18 (Cell proliferation-inducing gene 46 protein) (Cytokeratin-18) (CK-18) (Keratin-18) (K18)	0.40	0.031
P07711	*CTSL*	Procathepsin L (EC 3.4.22.15) (Cathepsin L1) (Major excreted protein) (MEP) [Cleaved into: Cathepsin L; Cathepsin L heavy chain; Cathepsin L light chain]	0.31	0.032
Q15113	*PCOLCE*	Procollagen C-endopeptidase enhancer 1 (Procollagen COOH-terminal proteinase enhancer 1) (PCPE-1) (Procollagen C-proteinase enhancer 1) (Type 1 procollagen C-proteinase enhancer protein) (Type I procollagen COOH-terminal proteinase enhancer)	0.20	0.032
P26368	*U2AF2*	Splicing factor U2AF 65 kDa subunit (U2 auxiliary factor 65 kDa subunit) (hU2AF(65)) (hU2AF65) (U2 snRNP auxiliary factor large subunit)	0.47	0.032
V9HW34	*HEL-213*	Epididymis luminal protein 213	0.57	0.032
A0A024R324	*RHOA*	Epididymis secretory sperm binding protein (Ras homolog gene family, member A, isoform CRA_a)	0.48	0.032
A0A0G2JPR0	*C4A*	C4a anaphylatoxin (Complement C4 gamma chain)	0.21	0.032
Q5T619	*ZNF648*	Zinc finger protein 648	0.42	0.033
A8K032		Translocating chain-associated membrane protein	1.37	0.033
A0A5C2GB45		IGH + IGL c305_light_IGKV2-28_IGKJ1 (Fragment)	0.50	0.033
O75947	*ATP5PD*	ATP synthase subunit d, mitochondrial (ATPase subunit d) (ATP synthase peripheral stalk subunit d)	0.73	0.033
O14672	*ADAM10*	Disintegrin and metalloproteinase domain-containing protein 10 (ADAM 10) (EC 3.4.24.81) (CDw156) (Kuzbanian protein homolog) (Mammalian disintegrin-metalloprotease) (CD antigen CD156c)	0.59	0.033
Q9BYD3	*MRPL4*	39S ribosomal protein L4, mitochondrial (L4mt) (MRP-L4) (Mitochondrial large ribosomal subunit protein uL4m)	0.66	0.033

P62328	*TMSB4X*	Thymosin beta-4 (T beta-4) (Fx) [Cleaved into: Hematopoietic system regulatory peptide (Seraspenide)]	0.52	0.034
A0A384NPU5		Epididymis secretory sperm binding protein	0.76	0.034
A0A024RDF4	*HNRPD*	Heterogeneous nuclear ribonucleoprotein D (AU-rich element RNA binding protein 1, 37kDa), isoform CRA_e	0.51	0.034
Q7Z406	*MYH14*	Myosin-14 (Myosin heavy chain 14) (Myosin heavy chain, non-muscle IIc) (Non-muscle myosin heavy chain IIc) (NMHC II-C)	1.32	0.034
P22792	*CPN2*	Carboxypeptidase N subunit 2 (Carboxypeptidase N 83 kDa chain) (Carboxypeptidase N large subunit) (Carboxypeptidase N polypeptide 2) (Carboxypeptidase N regulatory subunit)	0.41	0.034
Q59ER5		WD repeat-containing protein 1 isoform 1 variant (Fragment)	1.35	0.034
Q8IVH4	*MMAA*	Methylmalonic aciduria type A protein, mitochondrial (EC 3.6.-.-)	0.67	0.034
P13073	*COX4I1*	Cytochrome c oxidase subunit 4 isoform 1, mitochondrial (Cytochrome c oxidase polypeptide IV) (Cytochrome c oxidase subunit IV isoform 1) (COX IV-1)	0.73	0.034
Q5VZM2	*RRAGB*	Ras-related GTP-binding protein B (Rag B) (RagB) (EC 3.6.5.-)	0.35	0.034
A0A140VK84	*FN3KRP*	Protein-ribulosamine 3-kinase (EC 2.7.1.172)	0.68	0.034
A0A1B1HY05	*MYH6*	Myosin 6 (Fragment)	2.02	0.034
P78527	*PRKDC*	DNA-dependent protein kinase catalytic subunit (DNA-PK catalytic subunit) (DNA-PKcs) (EC 2.7.11.1) (DNPK1) (p460)	1.48	0.034
A0A024R702	*CGI-38*	Tubulin polymerization-promoting protein family member 3	0.72	0.035
P16402	*H1-3*	Histone H1.3 (Histone H1c) (Histone H1s-2)	0.58	0.035
P29558	*RBMS1*	RNA-binding motif, single-stranded-interacting protein 1 (Single-stranded DNA-binding protein MSSP-1) (Suppressor of CDC2 with RNA-binding motif 2)	0.33	0.035
Q8N5N7	*MRPL50*	39S ribosomal protein L50, mitochondrial (L50mt) (MRP-L50) (Mitochondrial large ribosomal subunit protein mL50)	2.51	0.036
V9HW37	*HEL-S-69*	Epididymis secretory protein Li 69	0.77	0.036
P28289	*TMOD1*	Tropomodulin-1 (Erythrocyte tropomodulin) (E-Tmod)	2.94	0.036

O43896	*KIF1C*	Kinesin-like protein KIF1C	1.46	0.036
Q9Y3Z3	*SAMHD1*	Deoxynucleoside triphosphate triphosphohydrolase SAMHD1 (dNTPase) (EC 3.1.5.-) (Dendritic cell-derived IFNG-induced protein) (DCIP) (Monocyte protein 5) (MOP-5) (SAM domain and HD domain-containing protein 1) (hSAMHD1)	0.68	0.036
Q9UBV2	*SEL1L*	Protein sel-1 homolog 1 (Suppressor of lin-12-like protein 1) (Sel-1L)	1.65	0.036
Q9HD42	*CHMP1A*	Charged multivesicular body protein 1a (Chromatin-modifying protein 1a) (CHMP1a) (Vacuolar protein sorting-associated protein 46-1) (Vps46-1) (hVps46-1)	1.30	0.037
A0A024RBH7	*TMPO*	Thymopoietin, isoform CRA_a	0.56	0.037
Q9BTW9	*TBCD*	Tubulin-specific chaperone D (Beta-tubulin cofactor D) (tfcD) (SSD-1) (Tubulin-folding cofactor D)	0.40	0.037
V9HW88	*HEL-S-99n*	Calreticulin	0.69	0.037
Q9BQ69	*MACROD1*	ADP-ribose glycohydrolase MACROD1 (MACRO domain-containing protein 1) (O-acetyl-ADP-ribose deacetylase MACROD1) (EC 3.1.1.106) (Protein LRP16) ([Protein ADP-ribosylaspartate] hydrolase MACROD1) (EC 3.2.2.-) ([Protein ADP-ribosylglutamate] hydrolase MACROD1) (EC 3.2.2.-)	2.37	0.038
Q92597	*NDRG1*	Protein NDRG1 (Differentiation-related gene 1 protein) (DRG-1) (N-myc downstream-regulated gene 1 protein) (Nickel-specific induction protein Cap43) (Reducing agents and tunicamycin-responsive protein) (RTP) (Rit42)	0.65	0.038
Q86VP6	*CAND1*	Cullin-associated NEDD8-dissociated protein 1 (Cullin-associated and neddylation-dissociated protein 1) (TBP-interacting protein of 120 kDa A) (TBP-interacting protein 120A) (p120 CAND1)	0.64	0.038
Q8WUD1	*RAB2B*	Ras-related protein Rab-2B	0.56	0.038
P00747	*PLG*	Plasminogen (EC 3.4.21.7) [Cleaved into: Plasmin heavy chain A; Activation peptide; Angiostatin; Plasmin heavy chain A, short form; Plasmin light chain B]	0.55	0.038
Q01449	*MYL7*	Myosin regulatory light chain 2, atrial isoform (MLC-2a) (MLC2a) (Myosin light chain 2a) (Myosin regulatory light chain 7)	0.43	0.038
O95299	*NDUFA10*	NADH dehydrogenase [ubiquinone] 1 alpha subcomplex subunit 10, mitochondrial (Complex I-42kD) (CI-42kD) (NADH-ubiquinone oxidoreductase 42 kDa subunit)	0.71	0.038

Q07954	*LRP1*	Prolow-density lipoprotein receptor-related protein 1 (LRP-1) (Alpha-2-macroglobulin receptor) (A2MR) (Apolipoprotein E receptor) (APOER) (CD antigen CD91) [Cleaved into: Low-density lipoprotein receptor-related protein 1 85 kDa subunit (LRP-85); Low-density lipoprotein receptor-related protein 1 515 kDa subunit (LRP-515); Low-density lipoprotein receptor-related protein 1 intracellular domain (LRPICD)]	0.61	0.038
O14925	*TIMM23*	Mitochondrial import inner membrane translocase subunit Tim23	1.47	0.038
Q9BWU5	*HBB*	Mutant hemoglobin beta chain (Fragment)	0.29	0.039
A0A140VJK7		5'-deoxynucleotidase HDDC2 (EC 3.1.3.89) (HD domain-containing protein 2)	0.35	0.039
Q53ZR1	*SLC12A2*	Bumetanide-sensitive Na-K-Cl cotransporter (Solute carrier family 12 (Sodium/potassium/chloride transporters), member 2, isoform CRA_b)	0.68	0.039
P05230	*FGF1*	Fibroblast growth factor 1 (FGF-1) (Acidic fibroblast growth factor) (aFGF) (Endothelial cell growth factor) (ECGF) (Heparin-binding growth factor 1) (HBGF-1)	0.49	0.039
A0A5C2GM29		IG c132_light_IGLV1-44_IGLJ2 (Fragment)	0.29	0.039
Q9NRG7	*SDR39U1*	Epimerase family protein SDR39U1 (EC 1.1.1.-) (Short-chain dehydrogenase/reductase family 39U member 1)	1.40	0.040
Q5VYK3	*ECPAS*	Proteasome adapter and scaffold protein ECM29 (Ecm29 proteasome adapter and scaffold) (Proteasome-associated protein ECM29 homolog)	1.38	0.041
Q8NCL6		cDNA FLJ90170 fis, clone MAMMA1000370, highly similar to Ig alpha-1 chain C region	0.45	0.041
P25685	*DNAJB1*	DnaJ homolog subfamily B member 1 (DnaJ protein homolog 1) (Heat shock 40 kDa protein 1) (HSP40) (Heat shock protein 40) (Human DnaJ protein 1) (hDj-1)	0.74	0.041
Q96AG3	*SLC25A46*	Solute carrier family 25 member 46	1.55	0.041
Q6ZVM7	*TOM1L2*	TOM1-like protein 2 (Target of Myb-like protein 2)	1.47	0.041
Q92504	*SLC39A7*	Zinc transporter SLC39A7 (Histidine-rich membrane protein Ke4) (Really interesting new gene 5 protein) (Solute carrier family 39 member 7) (Zrt-, Irt-like protein 7) (ZIP7)	0.55	0.041

O43678	*NDUFA2*	NADH dehydrogenase [ubiquinone] 1 alpha subcomplex subunit 2 (Complex I-B8) (CI-B8) (NADH-ubiquinone oxidoreductase B8 subunit)	0.51	0.041
A0A024R897	*ARPC5L*	Actin-related protein 2/3 complex subunit 5	0.43	0.041
P05413	*FABP3*	Fatty acid-binding protein, heart (Fatty acid-binding protein 3) (Heart-type fatty acid-binding protein) (H-FABP) (Mammary-derived growth inhibitor) (MDGI) (Muscle fatty acid-binding protein) (M-FABP)	1.37	0.042
B0I1S9	*MYO1B*	MYO1B variant protein (Myosin IB, isoform CRA_a)	2.29	0.042
P35659	*DEK*	Protein DEK	1.59	0.042
Q9BRX8	*PRXL2A*	Peroxiredoxin-like 2A (Peroxiredoxin-like 2 activated in M-CSF stimulated monocytes) (Protein PAMM) (Redox-regulatory protein FAM213A)	0.56	0.043
Q7L9L4	*MOB1B*	MOB kinase activator 1B (Mob1 homolog 1A) (Mob1A) (Mob1B) (Mps one binder kinase activator-like 1A)	0.63	0.043
A0A2R8Y504	*SGCE*	Epsilon-sarcoglycan	0.54	0.044
A0A0A0MSU4	*ABCA8*	ATP-binding cassette sub-family A member 8	0.57	0.044
F6IR49	*HLA-A*	MHC class I antigen (Fragment)	0.44	0.044
Q15599	*SLC9A3R2*	Na(+)/H(+) exchange regulatory cofactor NHE-RF2 (NHERF-2) (NHE3 kinase A regulatory protein E3KARP) (SRY-interacting protein 1) (SIP-1) (Sodium-hydrogen exchanger regulatory factor 2) (Solute carrier family 9 isoform A3 regulatory factor 2) (Tyrosine kinase activator protein 1) (TKA-1)	0.61	0.044
E9PFZ2	*CP*	Ceruloplasmin	0.52	0.044
Q5TZN6	*NOL3*	Nucleolar protein 3 (Apoptosis repressor with CARD domain) (Nucleolar protein 3 (Apoptosis repressor with CARD domain), isoform CRA_b) (cDNA, FLJ95803, Homo sapiens nucleolar protein 3 (apoptosis repressor with CARD domain) (NOL3), mRNA)	1.78	0.045
B0YIW2	*APOC3*	Apolipoprotein C-III (Apolipoprotein C3)	0.46	0.045
P62829	*RPL23*	60S ribosomal protein L23 (60S ribosomal protein L17) (Large ribosomal subunit protein uL14)	1.32	0.045
J3KSZ7	*SEPTIN4*	Septin-4	0.71	0.045

P51648	*ALDH3A2*	Aldehyde dehydrogenase family 3 member A2 (EC 1.2.1.3) (EC 1.2.1.94) (Aldehyde dehydrogenase 10) (Fatty aldehyde dehydrogenase) (Microsomal aldehyde dehydrogenase)	1.37	0.045
P28827	*PTPRM*	Receptor-type tyrosine-protein phosphatase mu (Protein-tyrosine phosphatase mu) (R-PTP-mu) (EC 3.1.3.48)	1.48	0.045
M0QXB5	*ETHE1*	Persulfide dioxygenase ETHE1, mitochondrial	0.53	0.045
A0A140VK07		Testicular secretory protein Li 7	0.63	0.045
Q9Y3B7	*MRPL11*	39S ribosomal protein L11, mitochondrial (L11mt) (MRP-L11) (Mitochondrial large ribosomal subunit protein uL11m)	0.67	0.045
Q96EI5	*TCEAL4*	Transcription elongation factor A protein-like 4 (TCEA-like protein 4) (Transcription elongation factor S-II protein-like 4)	0.53	0.045
P25787	*PSMA2*	Proteasome subunit alpha type-2 (Macropain subunit C3) (Multicatalytic endopeptidase complex subunit C3) (Proteasome component C3)	3.44	0.046
Q96DV4	*MRPL38*	39S ribosomal protein L38, mitochondrial (L38mt) (MRP-L38) (Mitochondrial large ribosomal subunit protein mL38)	0.62	0.046
Q6N095	*DKFZp686K03196*	Uncharacterized protein	0.76	0.046
P54646	*PRKAA2*	5'-AMP-activated protein kinase catalytic subunit alpha-2 (AMPK subunit alpha-2) (EC 2.7.11.1) (Acetyl-CoA carboxylase kinase) (ACACA kinase) (EC 2.7.11.27) (Hydroxymethylglutaryl-CoA reductase kinase) (HMGCR kinase) (EC 2.7.11.31)	1.60	0.047
Q8WV74	*NUDT8*	Nucleoside diphosphate-linked moiety X motif 8 (Nudix motif 8) (EC 3.6.1.-)	0.44	0.047
A0A097Q0T5	*COX2*	Cytochrome c oxidase subunit 2	0.68	0.047
O43716	*GATC*	Glutamyl-tRNA(Gln) amidotransferase subunit C, mitochondrial (Glu-AdT subunit C) (EC 6.3.5.-) (Protein 15E1.2)	0.55	0.047
Q9BX40	*LSM14B*	Protein LSM14 homolog B (RNA-associated protein 55B) (hRAP55B)	4.63	0.047
P00488	*F13A1*	Coagulation factor XIII A chain (Coagulation factor XIIIa) (EC 2.3.2.13) (Protein-glutamine gamma-glutamyltransferase A chain) (Transglutaminase A chain)	0.55	0.047

P60866	*RPS20*	40S ribosomal protein S20 (Small ribosomal subunit protein uS10)	1.44	0.047
P07737	*PFN1*	Profilin-1 (Epididymis tissue protein Li 184a) (Profilin I)	0.68	0.048
A5YM51	*MYH7*	MYH7 protein (Fragment)	6.37	0.048
A0A024R4N0		HCG1640809, isoform CRA_b	0.24	0.048
P43652	*AFM*	Afamin (Alpha-albumin) (Alpha-Alb)	0.60	0.048
Q9NR12	*PDLIM7*	PDZ and LIM domain protein 7 (LIM mineralization protein) (LMP) (Protein enigma)	0.37	0.048
Q9HA65	*TBC1D17*	TBC1 domain family member 17	1.62	0.048
D6R934	*C1QB*	Complement C1q subcomponent subunit B	1.88	0.049
A4D1B1	*CD36*	Glycoprotein IIIb (PAS IV) (PAS-4) (Platelet glycoprotein 4) (Platelet glycoprotein IV)	0.63	0.049
P32455	*GBP1*	Guanylate-binding protein 1 (EC 3.6.5.-) (GTP-binding protein 1) (GBP-1) (HuGBP-1) (Guanine nucleotide-binding protein 1) (Interferon-induced guanylate-binding protein 1)	0.55	0.049
Q9NQE9	*HINT3*	Histidine triad nucleotide-binding protein 3 (HINT-3) (EC 3.-.-.-)	0.59	0.049
P20774	*OGN*	Mimecan (Osteoglycin) (Osteoinductive factor) (OIF)	0.40	0.049
Q8N5J2	*MINDY1*	Ubiquitin carboxyl-terminal hydrolase MINDY-1 (EC 3.4.19.12) (Deubiquitinating enzyme MINDY-1) (Protein FAM63A)	2.40	0.049
P23327	*HRC*	Sarcoplasmic reticulum histidine-rich calcium-binding protein	1.72	0.049
Q12849	*GRSF1*	G-rich sequence factor 1 (GRSF-1)	0.60	0.049
Q9NR50	*EIF2B3*	Translation initiation factor eIF-2B subunit gamma (eIF-2B GDP-GTP exchange factor subunit gamma)	0.66	0.050
Q99685	*MGLL*	Monoglyceride lipase (MGL) (EC 3.1.1.23) (HU-K5) (Lysophospholipase homolog) (Lysophospholipase-like) (Monoacylglycerol lipase) (MAGL)	0.59	0.050
Q16647	*PTGIS*	Prostacyclin synthase (EC 5.3.99.4) (Hydroperoxy icosatetraenoate dehydratase) (EC 4.2.1.152) (Prostaglandin I2 synthase)	0.31	0.050
Proteins differentially (fold change >1.3 or <0.769) and significantly (P<0.05) expressed in AVS vs. CAD post-reperfusion RV samples. AVS, aortic valve stenosis; CAD, coronary artery disease; RV, right ventricle.				

Table SXVI. Significantly enriched canonical pathways for the relative protein analysis between AVS and CAD patients for post-reperfusion samples in the LV and RV.			
	Ingenuity canonical pathway	P-value of overlap	Molecules
LV	TREM1 Signaling	1.41E-03	GRB2, ITGA5, MAPK3, MPO, NFKB1
	PI3K/AKT Signaling	2.09E-03	GRB2, HSP90AA1, ITGA5, ITGB2, ITGB5, MAPK3, NFKB1, PIK3R1, RAF1, THEM4, YWHAB, YWHAG
	HGF Signaling	2.45E-03	GRB2, ITGA5, ITGB2, ITGB5, MAPK3, MAPK9, PIK3R1, PTPN11, RAF1
	Neuregulin Signaling	2.45E-03	GRB2, HSP90AA1, ITGA5, ITGB2, ITGB5, MAPK3, PIK3R1, PTPN11, RAF1
	Tumor Microenvironment Pathway	2.75E-03	FGF1, HLA-B, HLA-C, ITGA5, MAPK3, NFKB1, PIK3R1, RAF1, SLC2A3
	Regulation of Actin-based Motility by Rho	3.09E-03	GSN, ITGA5, ITGB2, ITGB5, MPRIP, MYL4, PFN2, PPP1R12B, RHOC
	Systemic Lupus Erythematosus Signaling	3.39E-03	C7, GRB2, HLA-B, HLA-C, LSM12, LSM14A, MAPK3, PIK3R1, PRPF19, PRPF6, PRPF8
	Actin Cytoskeleton Signaling	4.07E-03	FGF1, GRB2, GSN, ITGA5, ITGB2, ITGB5, MAPK3, MPRIP, MYH4, MYH7, MYL4, PFN2, PIK3R1, PPP1R12B, RAF1
	IL-6 Signaling	4.27E-03	CRP, CSNK2A1, GRB2, MAPK3, MAPK9, NFKB1, PIK3R1, PTPN11, RAF1
	Cdc42 Signaling	5.13E-03	HLA-B, HLA-C, ITGA5, ITGB2, ITGB5, MAPK9, MPRIP, MYL4, PPP1R12B, RAF1
	IL-2 Signaling	5.62E-03	CSNK2A1, GRB2, MAPK3, PIK3R1, PTPN11, RAF1
	PAK Signaling	5.75E-03	GRB2, ITGA5, ITGB2, ITGB5, MAPK3, MAPK9, MYL4, PIK3R1, RAF1
	Pancreatic Adenocarcinoma Signaling	6.76E-03	GPLD1, GRB2, MAPK3, MAPK9, NFKB1, PIK3R1, RAF1
	Lysine Degradation II	7.08E-03	AASDHPPT, AASS
	Caveolar-mediated Endocytosis Signaling	7.59E-03	COPG1, COPG2, HLA-B, HLA-C, ITGA5, ITGB2, ITGB5

	Prostate Cancer Signaling	8.51E-03	GRB2, GSTP1, HSP90AA1, MAPK3, NFKB1, PIK3R1, RAF1
	Phospholipase C Signaling	8.71E-03	GPLD1, GRB2, HDAC1, ITGA5, ITGB2, ITGB5, MAPK3, MPRIP, MYL4, NFKB1, PPP1R12B, RAF1, RHOC
	FGF Signaling	1.00E-02	FGF1, GRB2, MAPK3, PIK3R1, PTPN11, RAF1
	Th2 Pathway	1.10E-02	GRB2, HLA-B, ITGB2, NFKB1, PIK3R1
	Iron homeostasis signaling pathway	1.15E-02	ATP6V0D1, ATP6V1H, CIAO1, CIAO3, FTH1, HSPA9, MAPK3, NUBP2
RV	Epithelial Adherens Junction Signaling	8.91E-06	ARPC4, ARPC5L, CDC42, FGF1, MYH1, MYH14, MYH7, MYH9, MYL2, MYL3, MYL4, MYL7, PTPRM, RAC1, RALA, RHOA, YES1
	Regulation of Actin-based Motility by Rho	3.63E-05	ARPC4, ARPC5L, CDC42, GSN, MYL2, MYL3, MYL4, MYL7, PFN1, RAC1, RHOA, RHOT1, RHOT2
	Agranulocyte Adhesion and Diapedesis	3.98E-05	C5, CD34, GNAI2, MYH1, MYH14, MYH7, MYH9, MYL2, MYL3, MYL4, MYL7, PF4
	Oxidative Phosphorylation	4.79E-05	ATP5PD, ATPAF1, COX4I1, COX5B, COX6C, COX7A1, CYC1, MT-CO2, NDUFA10, NDUFA11, NDUFA13, NDUFA2, NDUFA9, NDUFB6, NDUFS8, NDUFV1
	Actin Cytoskeleton Signaling	4.90E-05	ARPC4, ARPC5L, CDC42, F2, FGF1, GSN, MYH1, MYH14, MYH7, MYH9, MYL2, MYL3, MYL4, MYL7, PFN1, PIK3R1, RAC1, RALA, RHOA, ROCK2, TMSB10/TMSB4X
	Complement System	1.58E-04	C1QA, C1QB, C1R, C1S, C4BPA, C5, C9, CFH
	Mitochondrial Dysfunction	2.19E-04	ACO2, ATP5PD, ATPAF1, COX4I1, COX5B, COX6C, COX7A1, CYC1, GPD2, MT-CO2, NDUFA10, NDUFA11, NDUFA13, NDUFA2, NDUFA9, NDUFB6, NDUFS8, NDUFV1, RHOT2
	Glioma Invasiveness Signaling	3.39E-04	CD44, CDC42, PIK3R1, PLG, RAC1, RALA, RHOA, RHOT1, RHOT2
	Calcium Signaling	3.72E-04	ATP2B4, CALR, MYH1, MYH14, MYH7, MYH9, MYL2, MYL3, MYL4, MYL7, PRKACA, TNNI3, TNNT2, TPM2
	CXCR4 Signaling	6.61E-04	CDC42, GNAI2, GNB4, MYL2, MYL3, MYL4, MYL7, PIK3R1, RAC1, RALA, RHOA, RHOT1, RHOT2, ROCK2
	ILK Signaling	7.76E-04	CDC42, KRT18, MYH1, MYH14, MYH7, MYH9, MYL2, MYL3, MYL4, MYL7, PIK3R1, RAC1, RHOA, RHOT1, RHOT2, TMSB10/TMSB4X

	IL-8 Signaling	7.76E-04	CDC42, DEFA1 (includes others), GNAI2, GNB4, MAP4K4, MPO, MYL2, MYL7, PIK3R1, RAC1, RALA, RHOA, RHOT1, RHOT2, ROCK2
	Intrinsic Prothrombin Activation Pathway	8.71E-04	COL2A1, F10, F13A1, F2, KLKB1, PROS1
	Hepatic Fibrosis / Hepatic Stellate Cell Activation	1.02E-03	COL15A1, COL2A1, FGF1, MYH1, MYH14, MYH7, MYH9, MYL2, MYL3, MYL4, MYL7
	RhoGDI Signaling	1.26E-03	ARPC4, ARPC5L, CD44, CDC42, GNAI2, GNB4, MYL2, MYL3, MYL4, MYL7, RAC1, RHOA, RHOT1, RHOT2, ROCK2
	Ephrin A Signaling	1.48E-03	ADAM10, CDC42, PIK3R1, RAC1, RHOA, ROCK2
	Cardiac Hypertrophy Signaling	1.78E-03	CDC42, EIF2B3, GNAI2, GNB4, MYL2, MYL3, MYL4, MYL7, PIK3R1, PRKACA, RAC1, RALA, RHOA, RHOT1, RHOT2, ROCK2
	Thrombin Signaling	2.00E-03	CDC42, F2, GNAI2, GNB4, MYL2, MYL3, MYL4, MYL7, PIK3R1, RAC1, RALA, RHOA, RHOT1, RHOT2, ROCK2
	Gα12/13 Signaling	2.51E-03	CDC42, F2, MYL2, MYL3, MYL4, MYL7, PIK3R1, RALA, RHOA, ROCK2
	Semaphorin Signaling in Neurons	2.69E-03	CDC42, DPYSL3, RAC1, RHOA, RHOT1, RHOT2, ROCK2

The top 20 most significant pathways are shown for each ventricle. AVS, aortic valve stenosis; CAD, coronary artery disease; RV, right ventricle; LV, left ventricle.

Table SXVII. Differentially expressed phosphoproteins from LV post-reperfusion samples between AVS and CAD patients.					
Accession No.	Gene name	Description	Phosphosite	Fold change	P-value
Q5VWP3	*MLIP*	Muscular LMNA-interacting protein (Cardiac Isl1-interacting protein) (CIP) (Muscular-enriched A-type laminin-interacting protein)	S9(Phospho)	0.45	0.001
E9PAV3	*NACA*	Nascent polypeptide-associated complex subunit alpha, muscle-specific form (Alpha-NAC, muscle-specific form) (skNAC)	S4(Phospho)	0.37	0.004
Q8NE71	*ABCF1*	ATP-binding cassette sub-family F member 1 (ATP-binding cassette 50) (TNF-alpha-stimulated ABC protein)	S5(Phospho)	3.34	0.004
A0A140VK83	*PPP1R7*	Protein phosphatase 1, regulatory subunit 7, isoform CRA_b (Testis secretory sperm-binding protein Li 210a)	S7(Phospho)	0.35	0.004
A0A024R1N1	*MYH9*	Myosin, heavy polypeptide 9, non-muscle, isoform CRA_a	S7(Phospho)	0.53	0.005
E9PAV3	*NACA*	Nascent polypeptide-associated complex subunit alpha, muscle-specific form (Alpha-NAC, muscle-specific form) (skNAC)	Ambiguous,	0.50	0.008
O14974	*PPP1R12A*	Protein phosphatase 1 regulatory subunit 12A (Myosin phosphatase-targeting subunit 1) (Myosin phosphatase target subunit 1) (Protein phosphatase myosin-binding subunit)	S20(Phospho)	0.60	0.010
E9PAV3	*NACA*	Nascent polypeptide-associated complex subunit alpha, muscle-specific form (Alpha-NAC, muscle-specific form) (skNAC)	S12(Phospho)	0.71	0.017
O00505	*KPNA3*	Importin subunit alpha-4 (Importin alpha Q2) (Qip2) (Karyopherin subunit alpha-3) (SRP1-gamma)	S11(Phospho)	1.78	0.020
A0A024R152		HCG28765, isoform CRA_b	S8(Phospho)	0.60	0.021
Q9GZY8	*MFF*	Mitochondrial fission factor	S1(Phospho)	1.66	0.023
O00629	*KPNA4*	Importin subunit alpha-3 (Importin alpha Q1) (Qip1) (Karyopherin subunit alpha-4)	S11(Phospho)	0.52	0.024
P0DMV9	*HSPA1B*	Heat shock 70 kDa protein 1B (Heat shock 70 kDa protein 2) (HSP70-2) (HSP70.2)	T8(Phospho)	0.52	0.032

E9PAV3	*NACA*	Nascent polypeptide-associated complex subunit alpha, muscle-specific form (Alpha-NAC, muscle-specific form) (skNAC)	S12(Phospho)	0.58	0.038
A0A024RD15	*MAPK14*	Mitogen-activated protein kinase 14 (EC 2.7.11.24) (Mitogen-activated protein kinase p38 alpha)	T7(Phospho), Y9(Phospho)	0.61	0.040
P35749	*MYH11*	Myosin-11 (Myosin heavy chain 11) (Myosin heavy chain, smooth muscle isoform) (SMMHC)	S8(Phospho)	0.64	0.044
O14874	*BCKDK*	[3-methyl-2-oxobutanoate dehydrogenase [lipoamide]] kinase, mitochondrial (EC 2.7.11.4) (Branched-chain alpha-ketoacid dehydrogenase kinase) (BCKD-kinase) (BCKDHKIN)	S1(Phospho)	0.49	0.046
D3DUZ3	*IFI16*	Interferon, gamma-inducible protein 16, isoform CRA_a (Interferon, gamma-inducible protein 16, isoform CRA_b)	S10(Phospho)	1.79	0.047
Q16851	*UGP2*	UTP--glucose-1-phosphate uridylyltransferase (EC 2.7.7.9) (UDP-glucose pyrophosphorylase) (UDPGP) (UGPase)	S3(Phospho)	0.70	0.049
Phosphoproteins differentially (fold change >1.3 or <0.769) and significantly (P<0.05) expressed in AVS vs. CAD post surgery samples from the LV. AVS, aortic valve stenosis; CAD, coronary artery disease; LV, left ventricle					

Table SXVIII. Differentially expressed phosphoproteins from RV post-reperfusion samples between AVS and CAD patients.					
Accession no.	Gene name	Description	Phosphosite	Fold change	P-value
P54105	*CLNS1A*	Methylosome subunit pICln (Chloride channel, nucleotide sensitive 1A) (Chloride conductance regulatory protein ICln) (I(Cln)) (Chloride ion current inducer protein) (ClCI) (Reticulocyte pICln)	S17(Phospho)	4.42	0.001
P04792	*HSPB1*	Heat shock protein beta-1 (HspB1) (28 kDa heat shock protein) (Estrogen-regulated 24 kDa protein) (Heat shock 27 kDa protein) (HSP 27) (Stress-responsive protein 27) (SRP27)	S3(Phospho)	0.69	0.003
E9PAV3	*NACA*	Nascent polypeptide-associated complex subunit alpha, muscle-specific form (Alpha-NAC, muscle-specific form) (skNAC)	S4(Phospho)	0.25	0.005
A0A024R152		HCG28765, isoform CRA_b	S8(Phospho)	0.73	0.005
A4D177	*CBX3*	Chromobox homolog 3 (HP1 gamma homolog, Drosophila) (Chromobox homolog 3 (HP1 gamma homolog, Drosophila), isoform CRA_a) (Coiled-coil domain containing 32, isoform CRA_c)	Ambiguous,	3.86	0.008
O94826	*TOMM70*	Mitochondrial import receptor subunit TOM70 (Mitochondrial precursor proteins import receptor) (Translocase of outer membrane 70 kDa subunit) (Translocase of outer mitochondrial membrane protein 70)	S2(Phospho)	2.87	0.014
Q13424	*SNTA1*	Alpha-1-syntrophin (59 kDa dystrophin-associated protein A1 acidic component 1) (Pro-TGF-alpha cytoplasmic domain-interacting protein 1) (TACIP1) (Syntrophin-1)	S3(Phospho), S4(Phospho)	0.64	0.016
C9JWC3	*SORBS2*	Sorbin and SH3 domain-containing protein 2 (Fragment)	S4(Phospho)	0.44	0.020
E9PAV3	*NACA*	Nascent polypeptide-associated complex subunit alpha, muscle-specific form (Alpha-NAC, muscle-specific form) (skNAC)	S12(Phospho)	0.42	0.022
Q6PKG0	*LARP1*	La-related protein 1 (La ribonucleoprotein domain family member 1)	S9(Phospho)	0.76	0.023

Q9BR39	*JPH2*	Junctophilin-2 (JP-2) (Junctophilin type 2) [Cleaved into: Junctophilin-2 N-terminal fragment (JP2NT)]	T15(Phospho), T2(Phospho)	1.36	0.024
Q2M3C7	*SPHKAP*	A-kinase anchor protein SPHKAP (SPHK1-interactor and AKAP domain-containing protein) (Sphingosine kinase type 1-interacting protein)	S15(Phospho)	1.84	0.032
A0A384MQX1		Epididymis secretory sperm binding protein	S2(Phospho), S3(Phospho)	0.48	0.032
O00505	*KPNA3*	Importin subunit alpha-4 (Importin alpha Q2) (Qip2) (Karyopherin subunit alpha-3) (SRP1-gamma)	S11(Phospho)	2.14	0.033
O60343	*TBC1D4*	TBC1 domain family member 4 (Akt substrate of 160 kDa) (AS160)	Ambiguous,	2.33	0.037
P10636	*MAPT*	Microtubule-associated protein tau (Neurofibrillary tangle protein) (Paired helical filament-tau) (PHF-tau)	S9(Phospho)	0.32	0.043
Q702N8	*XIRP1*	Xin actin-binding repeat-containing protein 1 (Cardiomyopathy-associated protein 1)	S15(Phospho)	0.35	0.044
Q9BR39	*JPH2*	Junctophilin-2 (JP-2) (Junctophilin type 2) [Cleaved into: Junctophilin-2 N-terminal fragment (JP2NT)]	S11(Phospho), T15(Phospho)	1.44	0.044
Q86TC9	*MYPN*	Myopalladin (145 kDa sarcomeric protein)	S6(Phospho)	1.48	0.044
Q15019	*SEPTIN2*	Septin-2 (Neural precursor cell expressed developmentally down-regulated protein 5) (NEDD-5)	S9(Phospho)	0.65	0.045

Phosphoproteins differentially (fold change >1.3 or <0.769) and significantly (P<0.05) expressed in AVS vs CAD post surgery samples from the RV. AVS, aortic valve stenosis; CAD, coronary artery disease; RV, right ventricle.

Table XIX. Significantly enriched canonical pathways for the relative phosphoprotein analysis between AVS and CAD patients for post-reperfusion samples in the LV and RV.			
	Ingenuity canonical pathway	P-value of overlap	Molecules
LV	Cellular Effects of Sildenafil (Viagra)	5.75E-06	MYH11, MYH7, MYH9, PDE3A, PLCL1, PLCL2, PPP1R12A, PRKAR2A
	Synaptic Long Term Potentiation	5.75E-06	CAMK2B, MAPK1, PLCL1, PLCL2, PPP1R12A, PPP1R7, PRKAR2A, RAF1
	Melatonin Signaling	3.02E-05	CAMK2B, MAPK1, PLCL1, PLCL2, PRKAR2A, RAF1
	Neuropathic Pain Signaling In Dorsal Horn Neurons	2.75E-04	CAMK2B, MAPK1, PLCL1, PLCL2, PRKAR2A
	Factors Promoting Cardiogenesis in Vertebrates	3.09E-04	CAMK2B, MAPK14, MYH7, PLCL1, PLCL2
	Leptin Signaling in Obesity	3.09E-04	MAPK1, PDE3A, PLCL1, PLCL2, PRKAR2A
	CDK5 Signaling	4.17E-04	MAPK1, MAPK14, PPP1R12A, PPP1R7, PRKAR2A, RAF1
	Endocannabinoid Cancer Inhibition Pathway	4.47E-04	EIF2A, MAPK1, MAPK14, PRKAR2A, RAF1, VIM
	Insulin Secretion Signaling Pathway	4.57E-04	CAMK2B, EIF2A, MAPK1, MAPK14, PDHA1, PLCL1, PLCL2, PRKAR2A
	Protein Kinase A Signaling	5.50E-04	CAMK2B, MAPK1, PDE3A, PLCL1, PLCL2, PPP1R12A, PPP1R7, PRKAR2A, RAF1, TTN
	Adrenomedullin signaling pathway	5.50E-04	MAPK1, MAPK14, PLCL1, PLCL2, PRKAR2A, RAF1, TTN
	Chemokine Signaling	5.50E-04	CAMK2B, MAPK1, MAPK14, PPP1R12A, RAF1
	Endocannabinoid Neuronal Synapse Pathway	1.07E-03	MAPK1, MAPK14, PLCL1, PLCL2, PRKAR2A
	Role of NFAT in Cardiac Hypertrophy	1.17E-03	CAMK2B, MAPK1, MAPK14, PLCL1, PLCL2, PRKAR2A, RAF1
	Actin Cytoskeleton Signaling	1.32E-03	KNG1, MAPK1, MYH11, MYH7, MYH9, PPP1R12A, RAF1, TTN
	Thrombin Signaling	1.62E-03	CAMK2B, MAPK1, MAPK14, PLCL1, PLCL2, PPP1R12A, RAF1
	ILK Signaling	1.70E-03	MAPK1, MYH11, MYH7, MYH9, NACA, PPP1R12A, VIM
	cAMP-mediated signaling	1.91E-03	CAMK2B, MAPK1, PDE3A, PRKAR2A, RAF1
	Calcium Signaling	1.95E-03	CAMK2B, MAPK1, MYH11, MYH7, MYH9, PRKAR2A

	PPARα/RXRα Activation	2.29E-03	MAPK1, MAPK14, PLCL1, PLCL2, PRKAR2A, RAF1
RV	p38 MAPK Signaling	1.10E-02	HSPB1,MAPT
	Netrin Signaling	1.45E-02	ABLIM1,ENAH
	ATM Signaling	2.24E-02	CBX3,USP7
	Death Receptor Signaling	2.45E-02	HSPB1,LMNA
	Cardiomyocyte Differentiation via BMP Receptors	2.95E-02	MYH7
	Prostanoid Biosynthesis	2.95E-02	PTGES3
	ILK Signaling	3.02E-02	MYH7,NACA,VIM
	GDP-glucose Biosynthesis	4.07E-02	PGM5
	Glucose and Glucose-1-phosphate Degradation	4.68E-02	PGM5

The top 20 most significant pathways are shown for the LV, while all significant pathways are shown for the RV. AVS, aortic valve stenosis; CAD, coronary artery disease; RV, right ventricle; LV, light ventricle.
